# The dynamic nature of netrin-1 and the structural basis for glycosaminoglycan fragment-induced filament formation

**DOI:** 10.1038/s41467-023-36692-w

**Published:** 2023-03-03

**Authors:** Markus Meier, Monika Gupta, Serife Akgül, Matthew McDougall, Thomas Imhof, Denise Nikodemus, Raphael Reuten, Aniel Moya-Torres, Vu To, Fraser Ferens, Fabian Heide, Gay Pauline Padilla-Meier, Philipp Kukura, Wenming Huang, Birgit Gerisch, Matthias Mörgelin, Kate Poole, Adam Antebi, Manuel Koch, Jörg Stetefeld

**Affiliations:** 1 Department of Chemistry, University of Manitoba, Winnipeg, Canada; 2 Center for Biochemistry II, Faculty of Medicine and University Hospital Cologne, University of Cologne, 50931 Cologne, Germany; 3 Max Planck Institute for Biology of Ageing, Cologne, Germany; 4 Faculty of Biology, Institute of Biology II, Albert Ludwigs University of Freiburg, Freiburg, Germany; 5 Institute of Experimental and Clinical Pharmacology and Toxicology, Medical Faculty, University of Freiburg, Freiburg, Germany; 6 Department of Obsterics and Gynecology, Medical Center, University of Freiburg, Freiburg, Germany; 7 Physical and Theoretical Chemistry Laboratory, Department of Chemistry, University of Oxford, South Parks Road, Oxford, OX1 3QZ UK; 8 Colzyx AB, Lund, Sweden; 9 Max Delbrück Center for Molecular Medicine, Robert Roessle Str 10, Berlin-Buch, Germany; 10 Cologne Excellence Cluster on Cellular Stress Responses in Aging Associated Diseases, University of Cologne, Cologne, 50931 Germany; 11 Institute for Dental Research and Oral Musculoskeletal Biology, Faculty of Medicine and University Hospital Cologne, University of Cologne, 50931 Cologne, Germany; 12 Center for Molecular Medicine Cologne, Faculty of Medicine and University Hospital Cologne, University of Cologne, 50931 Cologne, Germany; 13 Present Address: EMBL Australia Node in Single Molecule Science, School of Medical Sciences, Faculty of Medicine, University of New South Wales, Sydney, NSW Australia

**Keywords:** X-ray crystallography, Biophysical chemistry, Intracellular signalling peptides and proteins, Caenorhabditis elegans

## Abstract

Netrin-1 is a bifunctional chemotropic guidance cue that plays key roles in diverse cellular processes including axon pathfinding, cell migration, adhesion, differentiation, and survival. Here, we present a molecular understanding of netrin-1 mediated interactions with glycosaminoglycan chains of diverse heparan sulfate proteoglycans (HSPGs) and short heparin oligosaccharides. Whereas interactions with HSPGs act as platform to co-localise netrin-1 close to the cell surface, heparin oligosaccharides have a significant impact on the highly dynamic behaviour of netrin-1. Remarkably, the monomer-dimer equilibrium of netrin-1 in solution is abolished in the presence of heparin oligosaccharides and replaced with highly hierarchical and distinct super assemblies leading to unique, yet unknown netrin-1 filament formation. In our integrated approach we provide a molecular mechanism for the filament assembly which opens fresh paths towards a molecular understanding of netrin-1 functions.

## Abstract (web summary)

In this work, the authors report that heparin oligosaccharides have a significant impact on the highly dynamic behaviour of netrin-1 by inducing hierarchical and distinct super assemblies leading to unique, yet unknown netrin-1 filament formation.

## Introduction

Netrin-1 (NET1) is a chemotropic guidance cue pivotal to vertebrate development, where it plays a key role in attraction and repulsion of axons, cell migration, regulation of cell survival, and cellular differentiation^1,2^. It is secreted by axonal target cells and functions as both a short and long-range bifunctional guidance protein for axonal growth cones^3^. The dependence receptors deleted in colorectal cancer (DCC) and its paralogue neogenin (NEO1) are crucial for NET1-mediated axon attraction, cell-cell adhesion, and tissue organisation. In contrast, binding to UNC-5 triggers a chemorepellent response^4^. Disruption of the NET1 binding to its dependence receptors induces apoptosis (“dependence receptor hypothesis”)^5,6^.

The functionality of NET1 is driven by the formation of a chemotropic gradient, allowing fine control of its various signalling processes. It has been shown that NET1 diffuses in the extracellular matrix and creates a gradient that causes attraction of axons^7^. NET1 has been suggested to act as a chemotactic diffusible guidance factor, and as a haptotactic cue that promotes mechanotransduction^8^. However, the mechanism of the growth cone-NET1 interaction and its impact on attractive and repulsive responses based on the concentration gradient remains elusive.

Heparan sulphate proteoglycans (HSPGs) such as glypican are cell surface receptors that modulate NET1 signalling by acting as attachment points and modulators to fine-tune axonal responses^9^. It is known that the expression of HSPGs in commissural neurons is required for NET1-DCC mediated axon guidance^10^. It has been suggested that the HSPGs provide higher selectivity and reduce nonspecific interactions between NET1 and its receptors^11^. HSPGs contain extended glycosaminoglycan (GAG) chains that are highly acidic and form essential components of these receptors. However, what specific role these GAG chains play in the formation of NET1-mediated protein-protein networks has not been demonstrated. GAGs are involved in diseases such as inflammation, fibrinogenesis, wound healing, metastasis, blood coagulation and neurodegeneration^12^, and are known to have pro-tumorigenic properties and promote all aspects of tumour development and chemoresistance^13^. The enzyme heparanase, which is responsible for cleaving GAGs into shorter chains, is expressed by cells in the tumour microenvironment and is a dominant factor of the aggressive phenotype of cancer^14,15^. NET1 is known to specifically interact with heparin/heparan sulphate chains, while binding to neither chondroitin sulphate nor keratan sulphate could be detected^10,16,17^. Short heparin oligosaccharides dp2 to dp12 (dp - from degree of polymerisation) are widely distributed throughout the brain, and reduction of their levels lead to pathfinding errors^18^. In addition, it has been shown that heparin dodecasaccharides have several modulatory effects on metastasis and the growth of breast cancer cells^19^.

In this work, we examine the dynamics of an hierarchical NET1 oligomerization which is induced by short heparin oligosaccharides and leads to the formation of NET1 filaments. Interestingly, our studies highlight that this observed NET1 oligomerization does not necessitate the C-terminal NTR domain associated with heparin binding, confirmed by the use of a truncated NET1ΔC version. Further, we characterise the heparin/heparan sulphate binding surface within the LE-2 subdomain of NET1ΔC consisting of four highly charged amino acid patches.

Through binding assays on truncated NET1ΔC bearing mutations on the LE-2 heparin binding site, we show that partial mutation of this region is sufficient to diminish heparin and glypican-3 binding in vitro. We demonstrate by using neurite outgrow assays that disruption of the GAG-interaction leads to an inhibition of both branching initiation and neurite outgrowth. Further, our in vivo studies in *C. elegans* indicate that the heparin binding region within the LE-2 subdomain of NET1 is necessary for the proper migration of hermaphrodite gonads arms and interferes with the NET1 signalling pathway. Finally, we show that the heparan sulphate proteoglycan glypican regulates the assembly of NET1 multimer structures in *C. elegans*.

## Results

To probe the precise nature of NET1-HSPG and NET1-GAG interactions, we performed experiments at both the cellular and molecular level, using a NET1 version which is composed of domains LN (a.k.a. VI) and LE (a.k.a. V) which we designated NET1ΔC in this study (see also Methods section and Supplementary Fig. 1).

**Fig. 1:**
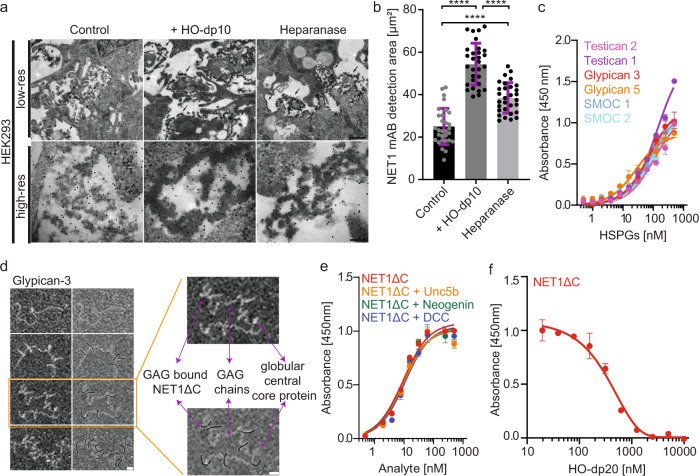
Localisation of NET1ΔC on the cell surface and different patterns of NET1ΔC-HSPG binding vs NET1ΔC-GAG mediated interactions. **a** Transmission immune electron micrographs of ultrathin sections of HEK-293 cells overexpressing NET1ΔC. A control image (left) is compared to addition of exogenous HO-dp10 (middle) and HEK-293 cells overexpressing of heparanase (right). The top and bottom row compare low- and high-resolution images. Black dots represent immune-gold labelled anti-NET1 mAB molecules depicting NET1ΔC deposits. The scale bars represent 200 nm (upper) and 20 nm (lower). **b** Statistical analysis of the appearance of immuno-gold labelled anti-NET1 mAb on the HEK-293 cell surface were analysed by counting the gold particles/μm^2^ on cellular profiles (*N* = 30). The results of an ordinary one-way ANOVA Tukey multiple comparisons test are indicated: ****, *P* < 0.0001. Mean and standard deviation (SD) are indicated. **c** ELISA style binding studies show protein-protein interactions of the heparan sulphate proteoglycans (HSPGs) glypican-3/5, SMOC-1/2, and testican-1/2 to immobilised NET1ΔC protein. NET1ΔC was coated and incubated with serial dilutions of strep-tagged HSPGs (0.5–500 nM). Binding was detected using a strep-HRP antibody. Error bars indicate the standard deviation from the mean of *N* = 3 technical repeats. **d** Negatively stained transmission electron micrographs of NET1ΔC-glypican-3 interactions. Glypican-3 exhibits a small globular central core protein to which three extended GAG chains are attached. Variable numbers of NET1ΔC molecules are bound to different locations along the GAG chains. The scale bar represents 20 nm. **e** ELISA style binding studies of immobilised glypican-3 to soluble NET1ΔC alone and together with ectodomains of DCC, neogenin and UNC5B, demonstrate NET1ΔC-mediated dependence receptor complex formation. NET1ΔC and receptor ectodomains were used in serial dilution from 0.5 to 500 nM (1:1 ratio of ligand to receptor). Binding was detected with a polyclonal, lab-made NET1 antibody. Error bars indicate the standard deviation from the mean of *N* = 3 technical repeats. **f** Competitive assays underline that a single-chain GAG competes with NET1ΔC-glypican-3 complex binding. Here, NET1ΔC (500 nM) was pre-incubated with HO-dp20 (1–1000 nM) and binding was detected against immobilised glypican-3 using NET1 antibody. Error bars indicate the standard deviation from the mean of *N* = 3 technical repeats.

First, we studied the localisation of NET1ΔC on the cell surface. Immuno-Gold labelled electron micrographs revealed a distinct pattern of NET1ΔC molecules on HEK-293 cell surfaces (Fig. 1a, b). Images of non-treated cells overexpressing NET1ΔC showed an evenly distributed lawn of NET1ΔC molecules which were linked to the plasma membrane. In contrast, cells incubated with HO-dp10 revealed a significantly increased concentration of NET1ΔC close to the cell surface. More strikingly, highly NET1ΔC-dense super-complexes of different length become visible. HEK-293 cells overexpressing human heparanase significantly diminished cell attachment of NET1ΔC molecules, yet the dense NET1ΔC assemblies persisted. Heparanase cleaves the GAG chains from HSPG, e.g., glypicans and syndecans, and hence releases the NET1ΔC bound to the HSPGs from the cell surface. At the same time, the GAG chains are being cleaved and short heparan sulphate fragments are formed and induce the dense NET1ΔC deposition. This switch between amorphous to a denser deposition of NET1ΔC led us to speculate that short HOs might induce the formation of distinct higher molecular NET1 aggregates.

We next screened a variety of different HSPGs to study the interaction with NET1ΔC (Fig. 1c). In addition to glypicans, we found that SMOCS, testicans and syndecans also formed high-affinity complexes with NET1ΔC. Our findings indicate that glypican-3 and syndecans-2 and −3 anchor NET1ΔC via their long GAG chains (Fig. 1d, Supplementary Fig. 1g).

More interestingly, while NET1ΔC could bind simultaneously to glypican-3 and the individual dependence receptors DCC, neogenin and UNC5B ectodomains (Fig. 1e), the dependence receptors themselves were not able to bind to glypican-3, with the exception of UNC5B (Supplementary Fig. 1c)^20^. While NET1ΔC bound HSPGs in the nM range (Fig. 1c), single-chain GAGs outcompeted NET1ΔC-glypican heterocomplexes (Fig. 1f). This demonstrates that the NET1ΔC-HSPG interaction is GAG-driven, and that the GAG polymer of cell surface HSPGs provides a ligand attachment site to colocalize NET1 in close proximity to the cell surface.

To identify the specific role of the GAG interactions, we further measured the binding profiles of NET1ΔC to its respective dependence receptor in the absence and presence of short (dp2-dp12) and medium (dp14-dp20) length HOs (Supplementary Fig. 1d). We observed that these GAG units had almost no impact on NET1ΔC-dependence receptor complex formation, suggesting that the binding epitopes on NET1 for GAGs and receptors are distinct and non-overlapping.

**Fig. 2:**
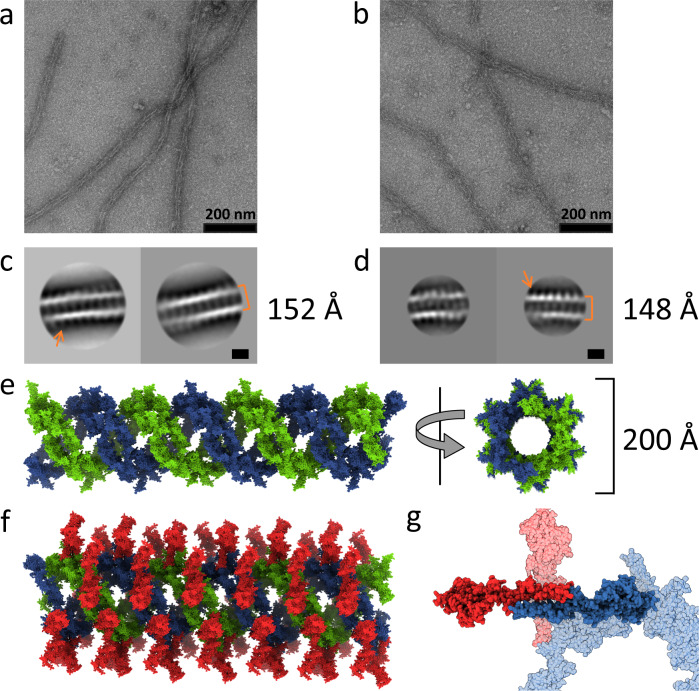
NET1 filament formation in the presence of heparin oligosaccharides. Representative negative stain images of NET1ΔC (**a**) in comparison to full-length NET1 (**b**) upon the addition of HO-dp10. Images show the formation of extended, filament-like structures (scale bar 200 nm). 2D class averages of NET1ΔC filaments (**c**) from ~3500 picked particles and class averages of full-length NET1 (**d**) from ~1500 particles. These class averages indicate a tubular assembly with approximate outer diameters of 152 Å (NET1ΔC) and 148 Å (full-length NET1), and repeating units projecting from the outside of the tube walls (arrows). Scale bars are 100 Å. **e** Double-helical model for NET1ΔC filament assembly as revealed in a low-resolution X-ray structure, 7LER. Crystal packing yields a tube-like double helical structure similar to that observed by negative stain. The outer diameter of the coil is 200 Å. **f** Additional NET1ΔC units (red), also present in the crystal structure, associate with the coil via an extensive and previously observed LE-LE interface of the SOS antiparallel dimer, 7LRF. **g** These units would generate the projections observed in the 2D class averages.

To gain molecular insight into the structure of the high-density HO-induced NET1 assemblies, we combined negative stain electron microscopy with X-ray crystallography (Fig. 2). Negative stain electron micrographs of NET1ΔC and full-length NET1 with short HOs (HO-dp10 and HO-dp12) revealed long, filament-like distinct structures of up to 5 μm in length and showed that NET1 can form a clustered meshwork (Fig. 2a, b, Supplementary Fig. 2). 2D class averages of sections of these tubular filaments showed a continuous, repeating arrangement with comparable outer diameters for both NET1ΔC and full-length NET1. This indicates that the positively charged C-terminal domain is not involved in heparin-induced filament formation. Repeating NET1 units project from the outer wall into solution (Fig. 2c, d). For both proteins, the layer separation along the length of the filament was ~54 Å. Crystallisation of the NET1ΔC filaments revealed many features reminiscent of the filaments observed by negative stain electron microscopy. The asymmetric unit of the X-ray structure was composed of 8 NET1ΔC molecules, which formed with symmetry translations a double helical core 200 Å across (Fig. 2e and Supplementary Fig. 3). A previously observed 623 Å^2^ buried interface was displayed along the length of the LE-2 and LE-3 subdomains, 4OVE, yielding NET1ΔC projections out from the core of the helix, similar to those in the 2D class averages of the electron micrographs (Fig. 2f, g).

Taken together, NET1 uses HSPGs as scaffold to co-localise at cell surfaces in close spatial vicinity to its transmembrane dependence receptors, an essential requirement for both outgrowth-promoting and chemotrophic or haptotactic effects^18^. Our results demonstrate that the GAG chains of HSPGs are binding partners of NET1, but do not directly mediate or interfere with dependence receptor complex formation. While NET1 establishes ternary complexes between glypican and its individual dependence receptors, single-chain short GAG’s do not act as coreceptors. Instead, in the presence of exogenous HO-dp10 and overexpressing heparanase that produces short-chain HOs, we observe a compacted more organised distribution of NET1 molecules and the induction of NET1 filament formation while the dependence-receptor binding properties remain intact.

**Fig. 3:**
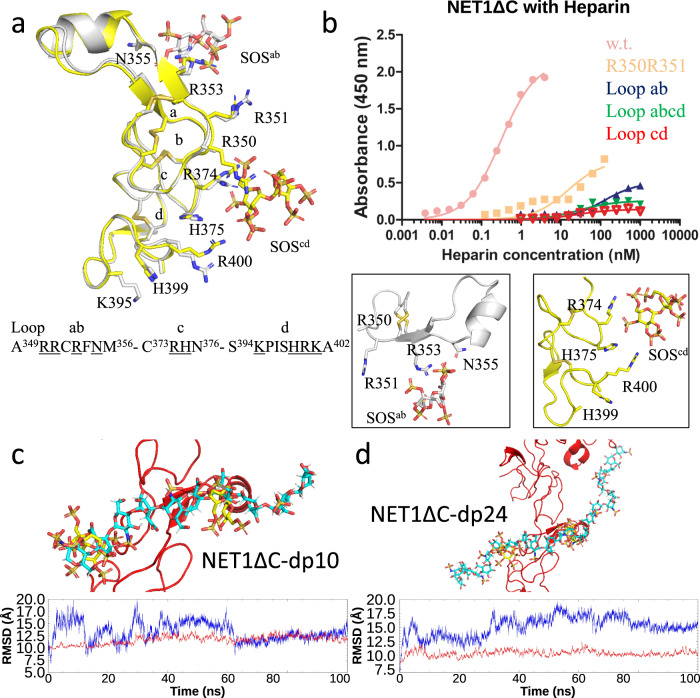
Molecular details of the NET1ΔC - SOS interaction. **a** SOS^ab^ (silver) and SOS^cd^ (yellow) binding motifs from our X-ray crystal structure. Individual loop segments are labelled **a**–**d**, accordingly. Amino acid residues studied in mutagenesis experiments are underlined and depicted as loop ab (RRXRXN), loop cd (RH-KHRK) mutant versions. **b** ELISA-based GAG-binding studies of wild type NET1ΔC and mutant versions of NET1ΔC. The coordinates of the mutated residues at the GAG binding sites are shown at the bottom of the panel. **c** Molecular dynamics cluster analysis of NET1ΔC-dp10 and (**d**) NET1ΔC-dp24 with SOS moieties shown in yellow. RMSD analysis shows a minimal change in the NET1-HO conformation (red) throughout the simulation, whereas the conformations of both HO-dp10 and HO-dp24 (blue) experienced some degree of flexibility. This is expected due to the flexible nature of the GAG molecule.

To investigate the structural basis of GAG recognition by NET1ΔC, we performed crystallographic studies in combination with site-directed mutagenesis and MD simulations (Fig. 3, Supplementary Table 1). Our crystal structure reveal an antiparallel NET1ΔC dimer in the asymmetric unit in complex with one sucrose-octasulfate molecule bound per monomer (Supplementary Fig. 4). Our findings assign the LE-2 subdomain of NET1 as the major GAG binding epitope. As shown previously, the laminin-type EGF-like fold of NET1 (LE-2) is highly electropositive in nature (IEP: 9.64), and is characterised by a cluster of arginine and lysine side chains that spans along all four loop segments (Fig. 3a, Supplementary Fig. 5)^21,22^. The GAG-binding epitope forms a linear extended structure which includes the Cardin-Weintraub motif RRXR (amino acid residues 350 − 352) from the loop ab segment, and the R374 - H375, H399 - R400 tandem of the loop cd pocket (Supplementary Fig. 6)^23^. Overall, the positively charged recognition surface area covers over 1085 Å^2^, offering extended recognition sites for acidic ligands. Remarkably, both SOS moieties, named SOS^ab^ and SOS^cd^, showed interaction plasticity and span a distance of ~26 Å across NET1ΔC. Comparisons with GAG structures in solution suggest that HO units with a degree of polymerisation of 8 or 10 (dp8 - dp10) adopt a helical pitch, which allows for maximum extensions of 23 − 30 Å^24,25^. In contrast to other protein-HO complexes, neither zinc nor calcium ions contribute to the positively charged character of the GAG binding site^26^. Both GAG-binding sites are defined by basic amino-acid residues interacting with negatively charged groups of the SOS moieties and additional binding energy is variably contributed by hydrogen bonding and van der Waal’s contacts via Y325, N355, H375 and H399.

To confirm our structural interpretation and to identify which individual amino acid residues are responsible for GAG binding, we produced a series of NET1ΔC mutations for functional assays, including alterations in the RRXR (loop ab) and the RH-HR (loop cd) motifs (Fig. 3b). Both sites are highly conserved in NET1 and in netrin-3, which is primarily expressed in sensory ganglia, suggesting a common GAG binding mode^27^. We observed that wild type NET1ΔC bound strongly to porcine heparin, whereas the RR-double mutant as well as the loop ab and the loop cd mutants showed reduced binding. The combined loop abcd mutant did not bind at all, validating the observed NET1ΔC-GAG interface. During molecular dynamics simulations of NET1ΔC in complex with HO-dp10 and HO-dp24, only modest overall displacement was observed for the individual GAG chains (Fig. 3c, d, Supplementary Fig. 7). Noticeably, a pivotal role for the RRXR and the RH-HR tandem is underlined by distance matrices and hydrogen bond lifetime occupations (watch Supplementary Movies 1, 2).

In summary, NET1 provides a remarkably extended GAG recognition surface by combining relatively short, widely spaced basic amino acid residue fragments to generate an extended high-affinity GAG-binding site. Both the Cardin-Weintraub motif and the RH-HR tandem motif contribute to NET1-mediated GAG interactions. Interaction affinities of NET1ΔC to glypican were in a similar range (apparent dissociation constant (*K*_*d*_) of 10–20 nM) as those determined for a single chain GAG units itself (*K*_*d*_ of 1–5 nM). The GAG-binding epitope of NET1 forms an extended linear array of ~1400 Å^2^ accessible surface area and the distinct SOS^ab^ and SOS^cd^ binding sites suggest that GAG chains of different length could associate with NET1.

**Fig. 4:**
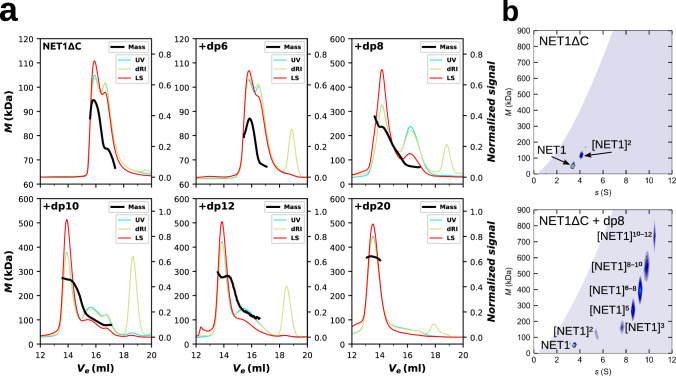
Dynamic nature of NET1ΔC - SEC-MALS and analytical ultracentrifugation. **a** SEC-MALS analysis of NET1ΔC in absence of HOs shows an equilibrium between monomeric and dimeric NET1ΔC. Upon the addition of HO-dp8, HO-dp10 and HO-dp12 we see a step-wise increase of mass with growing HO length. In the presence of HO-dp20, NET1ΔC forms a higher-order oligomer with a calculated mass of 346 kDa. Black lines show the mass distributions across the elution peaks which were traced by UV (*A*_280 nm_, cyan), differential refractive index (*dRI*_*658 nm*_, gold) and light scattering at 90° angle (665 nm, red). **b** *c(s, M)* distribution obtained from sedimentation velocity at 13 µM concentration without the addition of HOs reveal a monomer-dimer equilibrium (top). In the presence of HO-dp8 (1:1 and 1:2 molar ratios), NET1ΔC forms populations of particles with a stepwise increasing mass of ~160 kDa that form a ladder with an upper mass limit of 729 ± 49 kDa. Exact oligomer assignment is ambiguous for the larger species (Supplementary Tables 3 and 4).

**Fig. 5:**
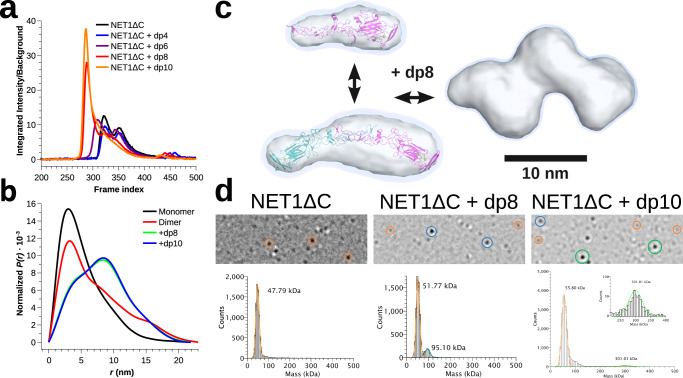
Dynamic nature of NET1ΔC - SEC-SAXS and mass photometry. **a** SEC-SAXS X-ray scattering trace of NET1ΔC while eluting from the SEC column. Pure NET1ΔC exists as a monomer-dimer equilibrium that assembles into a series of high-molecular species upon addition of HO-dp8 or HO-dp10, culminating in a sharp peak at the upper size limit. The mass determined from averaged 10–12 adjacent frames at the peak position yields a plausible range of 231–391 kDa (95% confidence interval, Supplementary Table 5c, d) which agrees with the SEC-MALS measurements of 235 kDa (Supplementary Table 2, Fig. 4a). **b** *P(r)* distributions calculated from the scattering profiles. **c** ED reconstruction from the scattering profiles of monomeric and dimeric NET1ΔC and upon addition of HO-dp8. The blue-shaded regions encompass the support volumes reported by DENSS. The surface representations are rendered at an ED level that encloses the particle volume reported by DAMMIN (see Supplementary Table 5). NET1ΔC structures were fit into the ED maps and are shown in cartoon representation. **d** Mass photometry of NET1ΔC in the nM concentration regime reveals a peak at 49 kDa without addition of HO. An additional peak appears at 95 kDa upon addition of HO-dp8 and a higher order oligomer peak at 300 kDa on addition of HO-dp10. Within the error of the measurement, we attribute these species to monomers (orange circle), dimers (blue), and hexamers (green).

At low μM concentration ranges, NET1ΔC exists in a dynamic equilibrium between its monomeric and dimeric state in solution (see Figs. 4, 5). The ability to self-associate differs markedly from its highly homologous counterpart, the soluble netrin-4, which is primarily monomeric in solution^28,29^. To study the effect of defined GAG species with distinct sizes (HO-dp6 to HO-dp20) on NET1ΔC oligomerization, we performed size exclusion chromatography coupled with multiangle light scattering (Fig. 4a). Incubation of NET1ΔC with HOs led to the formation of species of distinctly larger mass than monomers and dimers, confirming that NET1ΔC is associating with the HO chains. With increasing length of the HOs, we observed a gradual increase in mass and a shift from a heterogeneous to a homogeneous size distribution, including the disappearance of all monomeric/dimeric NET1ΔC species (Supplementary Fig. 8 and Supplementary Table 2).

The clustering behaviour was also measured by analytical ultracentrifugation and validated in-depth for the NET1ΔC-dp8 complex (Fig. 4b and Supplementary Fig. 9). Sedimentation velocity experiments revealed that in the absence of HOs, NET1ΔC exists in a monomer-dimer equilibrium in solution. Addition of an equimolar or twofold molar excess of HO-dp8 (~2.4 kDa) resulted in an increase in the sedimentation coefficient from 7.9 S to 10.2 S. Five clearly resolved populations were observed, representing discrete species of GAG-induced NET1ΔC complexes equivalent to trimers (~163 ± 26 kDa), pentamers (273 ± 48 kDa), and larger species with masses of 399 ± 65 kDa, 543 ± 52 kDa and 729 ± 49 kDa (Supplementary Tables 3 and 4). The larger species are expected to contain an unknown amount of dp8 chains which also contribute to the mass and make the exact oligomeric state determination ambiguous.

The monomer-dimer equilibrium of NET1ΔC in the absence of HOs was also observed by SEC-SAXS measurements. The presence of two overlapping elution peaks and single value decomposition indicate the existence of at least two major scattering components (Fig. 5a, Supplementary Figs. 10 and 11). Differences in volume, radius of gyration (*R*_g_), longest dimensions (*D*_max_) and estimated mass allow for an unambiguous assignment of the distinct species (Supplementary Table 5a, b). We generated ab initio electron density maps using DENSS^30^ from the deconvoluted signal of each scattering component. Rigid body fitting of a monomeric subunit of the NET1ΔC crystal structure into the map of the late eluting component and the non-crystallographic dimer into the map of the early eluting component confirmed their assignment to monomers and dimers, respectively (Fig. 5b, c, Supplementary Figs. 12–15). Addition of HO-dp8 or HO-dp10 caused a significant shift towards a single high-molecular weight peak accompanied with a significant increase in total envelope volume (Fig. 5a–c, Supplementary Table 5c, d; Supplementary Figs. 16–19). Bayesian mass estimates from the SEC-SAXS data suggest the presence of at least five individual NET1ΔC molecules^31^. The GAG-induced NET1ΔC oligomer can be defined as seed for the high-molecular weight assembly of NET1ΔC (see Supplementary Movie 3).

Finally, to validate our findings at more physiological concentrations, we performed single-molecule mass photometry measurements at 50–100 nM of NET1ΔC (Fig. 5d)^32^. At these concentrations, NET1ΔC is primarily monomeric, however, upon addition of HO-dp8, a larger dimeric population appeared which transitions to a higher-order hexameric NET1ΔC assembly upon addition of HO-dp10.

Our data provide compelling evidence that short-chain GAG binding is sufficient to induce NET1ΔC-clustering to form large super-complexes. The gradual transition from an existing monomer-dimer equilibrium points towards a mechanism in which higher-order NET1 assembly formation is the first step for building a heparin/heparan sulphate-induced concentration gradient. Short HOs not only impact the monomer-dimer equilibrium of NET1ΔC, but also induce high-molecular weight assemblies of NET1ΔC in a highly hierarchical manner.

**Fig. 6:**
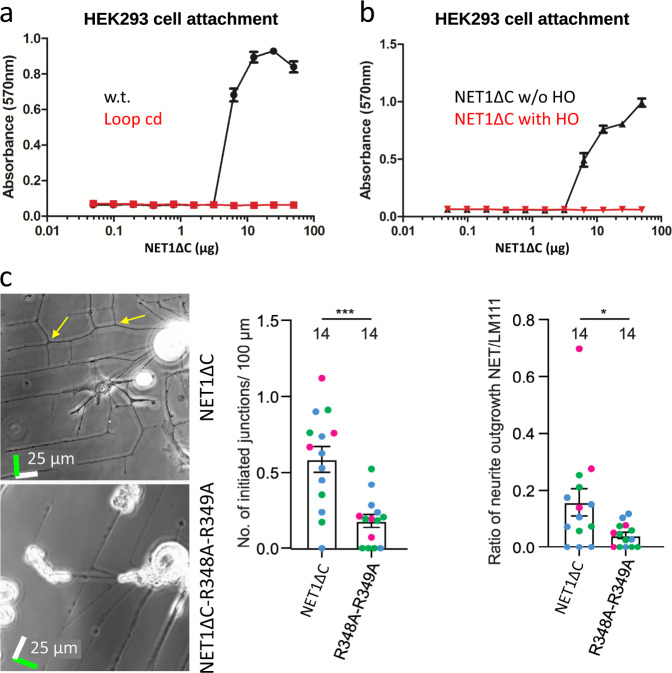
Physiological relevance of the NET1ΔC-GAG interaction. **a** Cell-attachment assay of wild type NET1ΔC in comparison to NET1ΔC-loop cd mutant. **b** Cell-attachment studies show that HO-dp20 is blocking NET1ΔC cell attachment. **c** Neuronal branching points (yellow arrows) of sensory neurons cultured on printed substrates. Bands of laminin-111 substrates were crossed with stripes of NET1ΔC and the NET1ΔC Cardin-Weintraub RR double mutant. Scale bars represent 25 µm and white bars indicate the direction of laminin-111 bands. Green depicts NET1ΔC and NET1ΔC-mutant stripes. More branch points were initiated on cells cultured on laminin-111 crossed with NET1ΔC compared with NET1ΔC R348A-R349A (*P* = 0.0002, Student’s *t* test, *N*_1_ = *N*_2_ = 14). More neurite outgrowth was supported on NET1ΔC compared to NET1ΔC-R348A-R349A (*P* = 0.02, Mann Whitney test, *N*_1_ = *N*_2_ = 14). Data are presented as mean ± s.e.m. with dots representing individual cells, colour indicates three distinct experiments.

To explore the physiological relevance of our results, we assessed the effect of LE-2 modifications in cell attachment and neuronal branching experiments (Fig. 6). HEK-293 cells were seeded on plates coated with recombinant NET1ΔC and NET1ΔC-mutants and cell adhesion was analysed (Fig. 6a, b). In agreement with our GAG-binding studies in vitro, NET1ΔC can act as direct cell adhesion substrate, but the GAG-binding motif was strictly required, since the addition of HO-dp20 and loop cd mutant versions of NET1ΔC completely abolished the cell binding. To test whether GAG-binding is a prerequisite for NET1ΔC-mediated neuronal branching, we scored the number of branch-point initiations when sensory neurons were cultured on patterned substrates (Fig. 6c)^33^. Primary sensory neurons were cultured on cross-hatched patterns of laminin-111 and either wild type NET1ΔC or a mutant NET1ΔC. Neurites predominantly extended over the laminin-111 and formed collateral branches on the wild type NET1ΔC. The number of branch point initialisations and the extent of neurite outgrowth was reduced on mutant NET1ΔC, compared to the wild-type protein. Our experiments demonstrate that disrupting the GAG-interaction leads to an inhibition of both branching initiation and neurite outgrowth.

**Fig. 7:**
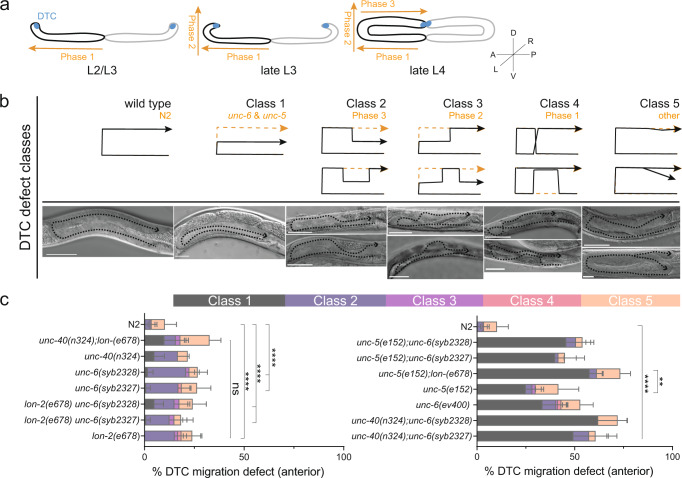
Physiological relevance of the NET1ΔC-GAG interaction in the model system *C. elegans* (part 1). **a** Schematic representation of post-embryonic hermaphrodite gonad development. Phase 1 migration begins along the anteroposterior (AP) axes followed by phase 2 leading the gonad arm along the dorsoventral (DV) axes. Phase 3 represents cessation of migration. Leading distal tip cells (DTC) are indicated in blue. **b** Representative Differential Interference Contrast (DIC) micrograph of gonad arms of L4 stage wild type animals in lateral view (first panel). Class 1 represents the classic *unc-5/unc-6* defect, classes 2–4 frequently observed defects during the indicated phase, class 5 mild or defects other than guidance defects. Gonad migration trajectories are indicated by black dotted lines. Scale bar: 20 µm. **c** Quantification of DTC migration defects of anterior gonad arms of wild type N2, NET1/*unc-6(ev400)* (null mutant), single and double mutants of NET1/*unc-6 (loop cd) mutants ((unc-6(syb2327) & unc-6(syb2328))*. Error bars represent mean ± SD. Results for ordinary 1-way ANOVA Tukey multiple comparisons test are indicated: ****, *P* < 0.0001; **, *P* < 0.0072; ns, not significant. Samples size for genotypes were *N* = 60 (crosses with *unc-40(n324)* and *unc-5(e152)*); *N* = 120 (crosses with HSPGs); *N* > 300 (wild type N2).

To determine the in vivo effects of the heparin-binding site in NET1, loop cd mutations were introduced into the homologous *unc-6* gene in *C. elegans* via CRISPR/Cas9, accordingly. Pivotal studies have described that *unc-6* null mutants display severe cell and growth cone migration defects as well as uncoordinated locomotion behaviour^34,35^. ELISA style binding assays performed on recombinantly expressed UNC-6ΔC and NET1ΔC have revealed that mutation of loop cd is sufficient to reduce heparin and glypican-3 binding, respectively (Supplementary Fig. 1e). To gain a comprehensive understanding to what extend the introduced mutation site affects HSPG binding in vivo, we generated double mutants with *lon-2*/glypican, *unc-52*/perlecan, and *sdn-1*/syndecan null mutants (Fig. 7 and Supplementary Fig. 20). We also generated double mutants with NET1-dependence receptor *unc-40*/DCC and *unc-5*/UNC-5 mutants to evaluate if the introduced mutation site interfere with the canonical *unc-6*/NET1 signalling pathway (Supplementary Table 7).

The introduced heparin binding mutations did not lead to egg-laying defects (Egl), nor locomotion defects (Unc). Unlike the *unc-6* null allele, both *unc-6(loop cd)* heparin binding mutations show normal locomotion behaviour, suggesting heparin binding sites play minor roles in axon guidance. This hypothesis is confirmed by our observations that neither guidance of circumferential neurons nor the development of hermaphrodite vulvas were affected in the *unc-6(loop cd)* mutants (Supplementary Fig. 20d, e). To analyse if heparin binding mutations affect cell migration, we examined gonad morphology in wild type and mutant worms at late L4 or stage. The hermaphrodite gonad arms of *C. elegans* led by the migration of distal tip cells (DTCs) are extending symmetrically in three phases during larval development (Fig. 7a)^36^. To better characterise the DTC migration defects, we introduced 5 defect classes according to the arise of observed DTC migration defects (Fig. 7b). Accordingly, DTC migration defects associated with classic *unc-6*/*unc-5* were grouped in class 1, DTC migration defects observed during the 3^rd^ migration phase in class 2, DTC migration defects observed during the 2^nd^ migration phase in class 3, DTC migration defects observed during the 1^st^ migration phase in class 4, and mild DTC migration defects or defects with another nature than migration failure in class 5. We found that ~20% of unc-6(loop cd) mutants exhibited pathfinding defects on their anterior gonad arm during the 3^rd^ DTC migration phase compared with ~3% in wild type animals (Fig. 7c). Comparing the phenotype from *unc-6(loop cd)* mutants with HSPG and NET1 dependence receptor mutant strains revealed that *lon-2*/glypican and *unc-40*/DCC show very similar moderate defects (Fig. 7c, right). However, *unc-40/*DCC, but not *lon-2/*glypican mutation, significantly enhances the observed DTC migration defects in *unc-6(loop cd)* mutant worms. The result is consistent with the previous ELISA style binding studies (Supplementary Fig. 1e) suggesting that *unc-6/*NET1 and *lon-2/*glypican work in the same pathway or that they physically interact. Interestingly, defects observed for the *unc-5/*UNC-5 single mutant resemble the defects observed in *unc-6(loop cd);unc-40* double mutant and the resemblance is further augmented by *lon-2/*glypican null mutation in the *unc-5/*UNC-5 mutant background (Fig. 7c, left). These observations indicate that the mutation site within *unc-6*/NET1 could be required to form a stable complex between *unc-40*/DCC|*unc-6*/NET1|*unc-5*/UNC-5 mediated by *lon-2*/glypican to regulate dependence receptor signalling. ELISA style binding assay support the notion that NET1 is required to bridge DCC-glypican-3 binding but not UNC5B-glypican-3 binding (Supplementary Fig. 1c). Surprisingly, *unc-40;lon-2* double mutants show an increased in class 5 defects, which is dominated by the defect previously described for transgenic worm strains overexpressing UNC-5 (Supplementary Fig. 20b)^37^, suggesting stage-specific roles for *unc-6*/NET1 receptors during DTC migration. Importantly, the described observation only holds true for the anterior gonad arm which suggest a different regulatory relationship on the posterior site of the worm (Supplementary Fig. 20c).

**Fig. 8:**
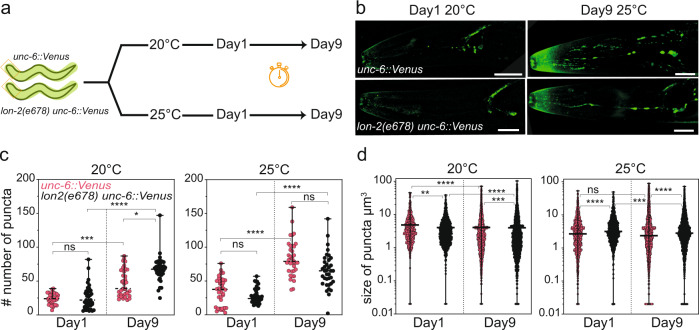
Physiological relevance of the NET1ΔC-GAG interaction in the model system *C. elegans* (part 2). **a** Schematic representation of workflow for NET1/UNC-6 puncta scoring. **b** Representative confocal fluorescent images of NET1/UNC-6 puncta in the head region of day 1 and day 9 adult worms grown at 20 °C or 25 °C. Scale bar: 20 µm. **c** Quantification of number of NET1/UNC-6 puncta per animal in the head region of day 1 and day 9 adult worms grown at 20 °C or 25 °C. Mean with range and results for Kruskal–Wallis Dunn’s multiple comparisons test are indicated: ****, *P* < 0.0001; ***, *P* < 0.0010; *, *P* < 0.0472; ns, not significant. For each genotype and condition between *N* = 36 to *N* = 39 worms were analysed. **d** Quantification of size distribution of NET1/UNC-6 puncta per genotype in the head region of day 1 and day 9 adult worms grown at 20 °C or 25 °C. Median with range and results for Kruskal–Wallis Dunn’s multiple comparisons test are indicated: ****, *P* < 0.0001; ***, *P* < 0.0004; **, *P* < 0.0027; ns, not significant. For each genotype and condition between *N* = 36 to *N* = 39 worms were analysed.

Our efforts to visualise UNC-6/NET1 at the distal tip cells remained unsuccessful so that an alternative approach was followed to assess if the observed NET1 filaments in vitro have an in vivo resemblance. We performed puncta scoring experiment as described for *C. elegans* models, which are used to study amyloidogenic proteins such as α-synuclein^38^. We quantified the fluorescent spots present in the region between the tip of the head and the end of the pharyngeal bulb for the Venus-tagged *unc-6* strains in a wild type and *lon-2*/glypican null background, *unc-6::Venus* & *lon-2(e678);unc-6::Venus*, respectively. To enhance the puncta formation, we grew worms at 20 °C and under temperature stress at 25 °C (Fig. 8a, b). The quantification of the number of puncta per worm and size of puncta were done at day 1 and at day 9 adulthood (Fig. 8c, d). These experiments revealed that the numbers of puncta for worms grown at 20 °C and 25 °C do not show significant differences when comparing *unc-6::Venus* with *lon-2(e678);unc-6::Venus* on day 1 and day 9. The number of puncta were increased in aged day 9 hermaphrodites when compared to day 1 hermaphrodites. Interestingly, the size distribution of the observed puncta were significantly altered when comparing *unc-6::Venus* with *lon-2(e678);unc-6::Venus* worms at day 1 as well as day 9 (Fig. 8d). Taken together, these, observations suggest a regulatory function of *lon-2*/glypican in *unc-6*/NET1 distribution. If the observed puncta are indeed aggregated or filamentous structures of UNC-6/NET1 remain to be answered.

## Discussion

Our cellular and molecular data are consistent with a model in which GAG chains of several HSPGs act as an entropic clamp, bringing NET1 in close spatial vicinity to the cell surface. In this way, NET1 is sequestered by glycosaminoglycans, providing a localised, readily accessible depot, yet available to activate cells by binding to its dependence receptors. In addition, we show that GAG chains do not act as co-receptors mediating NET1-dependence receptor interactions. Instead, we envision that the highly charged polysaccharide chains cause receptor clustering by the build-up of multimeric NET1 assemblies^39^, including the formation of extended NET1 filaments. Our observations are in alignment that HSPGs regulate NET1 function to ensure the proper development of cellular morphologies and its role in Axon guidance^9^.

NET1 is known to undergo posttranslational modifications leading to different isoforms^40,41^. We study the naturally occurring NET1 variant which lacks the C-terminal subdomain as a consequence of MMP9-cleavage, denoted NET1ΔC in this manuscript^41^. Previous studies have shown that NET1ΔC can act as adhesion molecule of eukaryotic cells and mediates interactions with the dependence receptors Unc-5^42^, DCC, and neogenin^43,44^. Furthermore, NET1ΔC is bioactive and provokes vascular permeability in diabetic retinopathy and is a potential therapeutic target to treat diabetic retinopathy^41^. Moreover, our data clearly demonstrate that both, NET1ΔC and full-length NET1 can form filaments.

The evolutionary conserved and unique Cardin-Weintraub motif (RRXR) alongside the RH-HR motif are responsible for NET1-cell attachment, initiation of neuronal branching, and pathfinding defects in *C. elegans*. We show compellingly that the identified heparin-binding region within the LE-2 subdomain of NET1 is necessary for the proper migration of hermaphrodite gonads arms and interferes with the NET1 signalling pathway. Further, we show through binding assays that partial mutation of this region is sufficient to diminish glypican-3 binding. The circumstance that single-chain short GAG’s are no co-receptors for NET1-mediated dependence receptor binding and that amino acid moieties accompanying the Cardin-Weintraub motif (Asn355) are involved in Unc5 binding suggests a dual mediator role of the extended motif. Whereas the RRXR mediates GAG interactions, other loop fragments of the LE-2 subdomain close to the RRXR motif mediate UNC-5 and DCC complex formation^21,44^. The finding that HO-dp8 and HO-dp10 are able to induce a hierarchical NET1 oligomerization is unique among GAG-interacting proteins^45^. Longer HO-chains (dp30) are required to tetramerize RPTPσ^46^ and hexamerize Shh^26^ or to cause establishment of the FGF–FGFR complex^47^. Other morphogens, such as Wnts are lipid-modified and form extracellular gradients by associating with lipoprotein particles^48^.

Our results point to a mechanism by which short GAGs modulate NET1 mobility and allow for fine-tuned diffusion by transferring the guidance cue from one HSPG to the next along the cell surface, at the same time establishing a chemotropic gradient. This is supported by our finding that in fluorophore-tagged *unc-6*/NET1 worm stains in a *lon-2*/glypican null background the size distribution of the observed UNC-6 puncta is significantly altered compared with the wild-type background. NET1 clustering by GAGs at the cell surface generates a platform for dependence receptor binding, which might limit diffusion and loss of NET1 into the extracellular space. In this way, dependence receptor binding and GAG-chain-induced multimerisation occurs independently, allowing NET1 to act simultaneously as a scaffold on different regulators. As a consequence, this may prevent nonspecific binding and aggregation of NET1 while preserving accessibility to the receptor binding sites.

The described NET1ΔC and full-length NET1 filaments are dynamic assemblies that appear to be induced by short GAG chains and our dependence receptor binding experiments with the NET1ΔC filaments reveal that their dependence receptor binding properties are still intact. Therefore, we suggest NET1 oligomerisation may be a regulatory mechanism that functions either to stabilise, sequester and/or locally amplify dependence receptor binding activity. Further studies will be necessary in understanding the role if the filaments in cancers and other human diseases. The modulation of the NET1 signalling pathway by small molecules targeting the GAG-binding site is a compelling strategy for the treatment of cancer and developmental disorders, and for stem cell therapies.

## Methods

### Heparin oligosaccharides

All heparin oligosaccharides (HOs) used in this study were sourced from Iduron, Alderley Edge, UK. dp8: cat #HO08; dp10: cat. # HO10; dp12: cat. # HO12; dp20: cat. # HO20.

### Recombinant protein expression and purification

NET1ΔC consisting of domains LN and LE (a.k.a. VI and V domains), full-length NET1 and the dependence receptors DCC, neogenin and UNC5B were expressed using the inducible sleeping beauty transposon system in human embryonic kidney (HEK) 293 T cells with a BM40 signal peptide and a N- or C-terminal Twin-Strep-tag or 8 histidine tag (*Gallus gallus* full length NET1 NP_990750 aa 26-606, *Gallus gallus* NET1ΔC NP_990750 aa 26-458, *Gallus gallus* NET1ΔC R350 R351 NP_990750 aa 26-458 R350A R351A, *Gallus gallus* NET1ΔC loop ab NP_990750 aa 26-458 R350A R351A R353A N355A, *Gallus gallus* NET1ΔC loop abcd NP_990750 aa 26-458 R350A R351A R353A N355A R374A H375A K395A H399A R400A K401A, *Gallus gallus* NET1ΔC loop cd NP_990750 aa 26-458 R374A H375A K395A H399A R400A K401A, *Mus musculus* NET1ΔC NP_032770 aa 24-457, *Mus musculus* NET1ΔC NP_032770 aa 24-457 R348A R349A, UNC5B NP_001346202 aa 25-304, *Rattus norvegicus* DCC short XP_017456347 aa 26-1077, DCC short NP_031857.2 aa 530-1024, *Rattus norvegicus* DCC long NP_036973.1 aa 26-1097, DCC long NP_031857.2 aa 530-1044, *Mus musculus* neogenin short NP_001036217.1 aa 42-1069, *Mus musculus* neogenin short NP_001036217.1 aa 573-1069, *Mus musculus* neogenin long NP_032710.2 aa 42-1085, *Mus musculus* neogenin long NP_032710.2 aa 573-1087, testican-1 NP_033288 aa 22-442, testican-2 NP_443720 aa 23-423, SMOC-1 NP_071711 aa 26-452, SMOC-2 NP_071710 22-447, syndecan-2 NP_032330 aa 19-146, syndecan-3 XP_017175579 aa 22-361, glypican-3 AF185614 aa 25-557 R357A, glypican-5 NP_780709 aa 25-549)^21,29,49^. Identified mutation sites were introduced into the UNC-6 sequence without the C-terminal domain (P34710 aa 18–459), including UNC-6ΔC (loop c: P34710 R378A H379A); UNC-6ΔC (loop d: P34710 H403A R404A K405A); and UNC-6ΔC (loop c-d: P34710 R378A H379A H403A R404A K405A). All protein versions were cloned into a sleeping beauty vector, transfected into HEK-293 cells, and expressed in serum-free DMEM/F12 medium. HEK-293 cells were transfected with FuGENE HD (Promega) and selected with 3 µg/ml puromycin for five days. Cells were grown to confluency in Dulbecco’s Modified Eagle’s Medium (DMEM/F12) supplemented with 10% foetal bovine serum before being transferred into HYPERFlask™ M cell culture vessels (Corning, New York, USA) or Nunc^TM^ TripleFlask^TM^ (ThermoFisher Scientific) for expression. Protein expression was induced with 0.5 µg/ml doxycycline after cells reached confluence and spent media were collected following 0.2 µm filtration for four times every two days. The conditioned media were applied to a 5 mL Strep-Tactin Superflow Plus cartridge (Qiagen Inc., Toronto, Canada) or Strep-Tactin®XT (IBA Lifescience, Göttingen, Germany) equilibrated with 50 mM tris, pH 8, 200 mM NaCl (*I* = 234 mM). After washing the bound protein with a high-ionic strength buffer (50 mM tris, pH 8, 1000 mM NaCl (*I* = 1035 mM), it was eluted with 50 mM tris, pH 8, 200 mM (*I* = 234 mM) containing 0.053% (w/v) d-desthiobiotin or 5 mM biotin and analysed by SDS-PAGE Coomassie dye staining. In the case of NET1ΔC, the Twin-Strep-tag® was removed by incubating the protein with 0.5 U thrombin/mg protein in combination with dialysis into 50 mM tris, pH 8.0, 1000 mM NaCl, 2.5 mM CaCl_2_ (*I* = 1042 mM) overnight. As final purification step, NET1ΔC was polished on a Superdex 200 increase column in 50 mM tris, pH 7.5, 500 mM NaCl and 500 mM (NH_4_)_2_SO_4_, before dialysing it into the final buffer (50 mM tris, pH 7.5, 200 mM NaCl). After tag removal and purification, an N-terminal sequence extension before residue 25 (APLA) and a C-terminal extension after residue 458 (GSLVPR) remained on the protein in comparison to the UniProt sequence. The quality of all proteins was checked by SDS-PAGE and dynamic light scattering before setting up crystal trays for screening. An EXCEL file listing the primers used for cloning is included with the Supplementary Data.

### Antibody production

The ectodomains from *Mus musculus* DCC, neogenin, and UNC5B were amplified by PCR and ligated into a modified PCEP-4 expression vector (gift from Ernst Poeschl) containing a His-8 tag and a BM40 signal peptide. 293-EBNA cells (Invitrogen) were transformed (FuGENE HD; Promega GmbH) with the expression vector and selected after 2 days with puromycin (SigmaAldrich). Conditioned media were applied onto nickel-chelated Sepharose columns (Thermo Fisher Scientific) and after extensive wash with the binding buffer (200 mm NaCl, 20 mm tris-HCl, pH 8) and eluted stepwise by applying binding buffer containing increasing concentrations of imidazole (10–150 mm). The purified recombinant proteins were used to immunise rabbits. The antisera obtained were purified by affinity chromatography on columns with antigens coupled to CNBr-activated Sepharose (Thermo Fisher Scientific). The specific antibodies were eluted with 150 mm NaCl, 0.1 m triethylamine, pH 11.5 and neutralised with 1 m tris-HCl, pH 6.8. To verify the specificity of the different antibodies, western blot analysis with the supernatants from cells expressing the DCC, neogenin, and UNC5B ectodomains were performed.

### ELISA style binding assays

Biotinylation of heparin was performed as follows^50^: Porcine heparin (grade 1 A, sodium salt, Sigma-Aldrich cat. H3393) was activated using epichlorohydrin and 1,6-diaminohexane prior to biotinylation with Biotin-X-NHS (Sigma-Aldrich). For the NET1ΔC-heparin and UNC-6ΔC-heparin binding assays strep-tag purified netrins were diluted in TBS, pH 7.4, and 10 μg/ml (0.5 µg/well) were coated onto 96-well plates (Nunc® MaxiSorp™) at 4 °C overnight. After washing with TBS, unspecific binding sites were blocked at room temperature with 100 µl 3% BSA in TBS for 1 h. Biotinylated heparin was then serial diluted in blocking buffer to concentrations from 1 pM to 1 µM in 50 µL per well. After 1 hour incubation at room temperature the ELISA plate was carefully washed three times with TBS buffer and a Biotin-HRP antibody 1:10000 (Aviva Systems Biology) in blocking buffer was applied for 1 hour at room temperature. Afterwards the plate was washed three times. Horseradish peroxidase activity was detected utilising the 1-Step Ultra TMB ELISA substrate solution (ThermoFisher Scientific). The reaction was stopped with 50 µl 10% H_2_SO_4_ and the absorbance was measured at 450 nm.

For the NET1ΔC-heparin and UNC-6ΔC-heparin and the dependence receptor assays, ELISA 96-well plates (Nunc® MaxiSorp™) were coated with His-tagged NET1ΔC or UNC-6ΔC (0.5 µg/well in TBS buffer) and incubated overnight at 4 °C. After two wash steps with TBS buffer, unspecific binding sites were blocked with 3% BSA in TBS buffer/2 mM CaCl_2_ for 1 hour. For heparin competition experiments 1 µM HO-dp12 or HO-dp20 were added to lanes 5–8 of the 96-well plate. The lanes 1–4 were incubated with empty blocking buffer. After 1 hour incubation the plate was carefully washed with TBS-buffer/2 mM CaCl_2_ or 20 mM HEPES, 100 mM NaCl, 2 mM CaCl_2_, pH 6.5. The ligands were added in a serial dilution starting from 1 µM ligand in TBS based blocking solution or HEPES buffer-based blocking solution. After 90 minutes the ELISA plate was washed twice with HEPES buffer and then fixed for 10 min with 1% glutaraldehyde in HEPES buffer. Next the bound ligand was detected after 1 hour incubation with a Strep-HRP conjugate (IBA Lifesciences), the plate was washed three times, and 50 µl 1-Step Ultra TMB ELISA substrate solution (ThermoFisher Scientific) was applied. After the reaction was stopped with 50 µL 10% H_2_SO_4_ the absorbance was measured at 450 nm.

To test dependence receptor binding to glypican-3, 96-well plates (Nunc® MaxiSorp™) were coated with glypican-3 (0.5 µg/well in TBS buffer) and incubated overnight at +4 °C. Unspecific binding sites were blocked with 3% BSA in TBS buffer/2 mM CaCl_2_ for 1 hour, followed by a washing step. Subsequently, serial dilutions of NET1-dependent receptors were added to the plates, either alone or in a 1:1 and 1:2 mixture with NET1ΔC, preincubated for 1 h at RT. After 1 h incubation protein binding was detected with home-made polyclonal affinity purified rabbit antibodies against NET1, DCC, neogenin and UNC5B.

Competitive ELISA studies were performed by adding GAG (0−1 μM) to NET1ΔC (500 nM) in solution for 1 hour. Subsequently, a serial dilution was applied on 96-well plates (Nunc® MaxiSorp™) coated with glypican-3. The binding analysis was performed as described above.

### Cell attachment assays

96-well plates (Nunc® MaxiSorp™) were coated with a 1:2 serial dilution of chicken NET1ΔC or NET1ΔC mut loop cd, starting from 50 µg/ml in PBS overnight at 4 °C. Unspecific binding sites were blocked with 100 µL solution of 1% BSA/PBS for 3 hours at 4 °C. For the HO preincubation experiment 1 µM HO-dp20 was added to the blocking solution and all the plates were blocked for 1 additional hour. HEK-293 cells at 60% confluence were trypsinised. After resuspension in DMEM medium, the cells were counted and diluted to 5 ∙ 10^5^ cells/ml. The cell suspension was then briefly vortexed, and 1 mM MnCl_2_ and 2 mM MgCl_2_ were added prior to the pipetting of 100 µl cell suspension into each well. Next, the plate was placed in a 37 °C incubator for 30 minutes and thereafter carefully washed with PBS buffer and fixed with fresh 1% glutaraldehyde for 15 min. Then the cells were stained with 0.1% crystal violet in H_2_O for 25 min followed by an extensively wash with tap water. The cells were then lysed with 50 µl 0.2% Triton X-100 and the absorbance of the lysate was measured with an ELISA reader at 570 nm.

### Crystallisation and structure determination

Purified NET1ΔC was concentrated to 9–12 mg/ml. The concentration was measured spectrophotometrically at 280 nm using extinction coefficient of 49455 M^−1^cm^−1^, that was calculated from sequence using the ExPASy ProtParam tool^51^. Crystallisation experiments were performed at 293 K employing the vapour diffusion technique. Sitting droplets were made by mixing equal volume of protein solution and 20% w/v polyethylene glycol 8000 and 100 mM CHES pH 9.5. The crystals belong to spacegroup P2_1_2_1_2_1_ and contain an antiparallel NET1ΔC dimer in the asymmetric unit. For subsequent complex determinations, native crystals were soaked for 5–7 days in 10 mM SOS. X-ray diffraction experiments were performed on a Rigaku Micromax-007 HF (λ = 1.5418 Å) at 100 K and the X-ray diffraction data were processed with iMOSFLM^52^. Statistics of the merged data are given (Supplementary Table 1). Extensive co-crystallisation attempts with HOs did not produce NET1ΔC crystals suitable for X-ray diffraction experiments. The structure was determined by molecular replacement employing PHASER^53,54^ using the crystal structure of mouse NET1ΔC, 4OVE as a search model^21^. Refinement with Phenix^55^ was alternated with manual electron density refitting of side chains and N-glycan carbohydrate attachments using COOT^56^. Refinement strategies included bulk solvent correction and anisotropic scaling of the data, individual coordinate refinement and TLS parameterisation. After including all native amino acids residues, proper fitting of all four N-glycan attachments and inclusion of water molecules and ions the *R*_value_/*R*_free_ fell to 26.8/33.1. At this stage, both SOS molecules have been fitted into a 3σ contoured Sigma-A weighted Fo-Fc difference Fourier map using the coordinates of SOS^57^. Interpretation of the electron density maps for each solution together with monitoring of the *R*_value_/*R*_free_ ratio revealed that each NET1ΔC monomer binds one SOS molecule. The quality of the structure was validated with SwissModel and PROCHECK^51,58^. The final *R*_value_/*R*_free_ factors for the SOS, 7LRF were refined to 22.7/27.9. The final model comprises amino acid residues P40 - P357 and contains two calcium ions, one sodium ion, and one chloride ion, four 1,2-ethanediol (EDO), 2 polyethylene glycol (PEG) molecules, one CHES (2-[N-cyclohexylamino] ethane sulphonic acid) and 44 water molecules. The side chains of N97, N118, N133 and N419 are N-glycosylated. The carbohydrate structures were validated using PDB-CARE and CARP^59–61^.

Crystallisation of the NET1ΔC filament crystals was performed by sitting drop vapour diffusion in 0.1 M tris, pH 8.25, 41% 2-methyl-2,4-pentanediol at a protein concentration of 14.2 mg/ml. Data were collected at the 08B1-1 beamline at the Canadian Light Source with no additional cryoprotectant. Data were collected for a 180° rotation using a 1° oscillation angle at a wavelength of 0.97952 Å and an exposure of 30 s. The 6.0 Å dataset was integrated and scaled using XDS and XSCAL^62^ and merged using Aimless^63^. The structure was determined by molecular replacement employing PHASER^53,54^ using the crystal structure of mouse NET1ΔC 4OVE as a search model^21^. Model building and refinement was completed with the Phenix software package^64^ and Coot^56^. Interface contacts were determined using FastContact and electrostatic potentials were generated using APBS^65,66^. Buried surface area values of protein-protein interactions were calculated using the PISA webserver for a probe radius of 1.4 Å^67^. Structure figures were prepared using the programme PyMOL (Molecular Graphics System, Schrödinger, LLC), ChimeraX^68^ and sequence conservation analysis was performed using MULTILAN^69^ and ESCRIPT^70^. The final crystallographic data are summarised in Supplementary Table 1.

### Molecular dynamics simulation

The NET1ΔC-SOS complex structure, 7LRF was used as starting model for the MD simulations. Both, HO-dp10 and HO-dp24, were positioned in close spatial vicinity to the SOS^ab^ and SOS^cd^ binding sites and energy minimised using the MODELLER software package^71^. For the NET1ΔC-dp10 complex, the GLYCAM server was used for proper decasaccharide unit definition, whereas for the NET1ΔC-dp24 complex the first conformer of the HO-dp24 ensemble 3IRJ was used as described in Khan et al.^24^. Since there was no GROMACS compatible version of GLYCAM06j, the topology and parameters of dp24 were converted from AMBER format to GROMACS format using ACPYPE tool^72,73^. The MD simulations for both NET1ΔC complexes were carried out in GROMACS 2020 package with two compatible force fields. The ff14SB force field^74^ was applied to the NET1ΔC protein and the GLYCAM06j to HO-dp10 and HO-dp24^75^. For both simulations, the complex was solvated in a TIP3P dodecahedron periodic box with a minimal distance to the periodic box border of 10 Å. Sodium and chloride ions (Na^+^ and Cl^−^) were added as counter ions and to maintain the salt concentration of 200 mM. The whole system was subject to 5000 steps of steepest decent minimisation, and subsequently heated from 0 to 300 K for 100 ps in the canonical ensemble (NVT ensemble), applying harmonic restraints with a force constant of 1000 kJ/mol nm^2^ on both the protein and respective HOs. The systems were then equilibrated in an isothermal isobaric ensemble (NPT ensemble) under constant pressure (105 Pa) for 100 ps. Finally, productive MD runs were carried out to generate a 100 ns long simulation trajectory. LINCS algorithm, time integration step of 2 fs, short-range interactions cutoff of 12 Å, and Particle Mesh Ewald method were used. Protein-ligand model and simulation trajectory files are available on the Centre for Open Science (OSF) database [10.17605/osf.io/kn4q2].

The generated trajectories were analysed using VMD and its plugins^76^. The root-mean-square deviation (RMSD) value is a useful estimation for quantifying conformational changes and was calculated for the whole system and for individual HOs alone. Hydrogen bonds facilitating NET1ΔC-HO interactions were determined from the MD simulation trajectory by finding the heavy atom pairs that have distances below 3.5 Å^77^. Cluster analysis was done to group trajectory frames that have similar conformation of the individual HOs. Each cluster is represented by a frame that has the lowest RMSD compared to the average structure of the cluster by applying a cutoff of 5 Å.

### Transmission and negative stain electron microscopy

Human heparanase (AAD54516; AA:37-545) was cloned into the sleeping beauty expression vector containing a N-terminal BM40 signal peptide followed by a Twin-strep tag. *Gallus gallus* NET1ΔC (NP990750; AA:26-458) was cloned into the expression vector containing a N-terminal BM40 signal peptide and a C-terminal Twin-strep tag. NET1ΔC alone or together with heparanase (20%) were transfected into HEK-293 cells, selected, and cultured for four days in the presence of 0.5 µg/ml DOX. For an additional three days, 6 µg/ml of HO-dp12 was added to some of the cultures. After three days, the medium was removed and the cultures were fixed with 4% paraformaldehyde and 2.5% glutaraldehyde in PBS, pH7.4. The whole cell sheets were carefully removed and processed for transmission electron microscopy and immunostaining. In short, the fixed and washed samples were subsequently dehydrated in ethanol and further processed for standard Epon embedding. Sections were cut with an LKB ultratome and mounted on Formvar-coated copper grids. The sections were post fixed with uranyl acetate and lead citrate and examined in a Philips/FEI CM100 BioTwin transmission electron microscope operated at a 60-kV accelerating voltage. Images were recorded with a Gatan Multiscan 791 charge-coupled device camera. The ultrathin sections were stained with uranyl acetate (Laurylab, Saint Fons, France) and lead citrate (Laurylab). Immunolabelling of thin sections after antigen unmasking with sodium metaperiodate (Merck) with gold-labelled anti-NET1 mAb was performed following a published protocol^21,78^. To quantify NET1ΔC deposits, we have performed the statistical analysis in Photoshop CS6 by manually counting the gold particles/μm^2^ for 30 cellular profiles. The appearance of immuno-gold labelled anti-NET1 mAb at the HEK-293 cell surface in the control has been compared with cells treated with exogenous HO-dp10 and cells overexpressing haparanase.

Regarding the interaction of NET1ΔC with HSPGs, purified NET1ΔC samples were incubated with equimolar amounts of syndecan-2/3 or glypican-3, respectively, for 1 h at 37 °C in TBS (150 mM NaCl, 50 mM tris-HCl, pH 7.5). NET1ΔC and full-length NET1 filaments were prepared by incubating the protein with 10 μg/ml HO-dp10 in TBS for 1 h at 37 °C. Prior to negative staining and electron microscopy^79,80^, specimens were diluted with TBS to 20 nM concentration. 5 μl-samples were then adsorbed to 400 mesh carbon-coated grids which had been rendered hydrophilic using a glow-discharger at low vacuum conditions. They were subsequently stained on 5 μl drops of 2% (w/v) uranyl acetate. Samples were examined in a Philips/FEI CM 100 TWIN transmission electron microscope (FEI Co, Hillsboro, OR, USA) at 60 kV accelerating voltage. Images were recorded with a side-mounted Olympus Veleta camera 4k with a resolution of 2048 × 2048 pixels (2k × 2 K) using ITEM^TM^ software.

NET1ΔC loop ab mutant or NETΔC loop cd mutant were incubated for 2 h at 37 °C at similar conditions are described above. 5 µl samples were pipetted on glow-discharged carbon coated copper grids and excessive liquid was removed using filter paper. After negative staining with 0.75 % uranyl acetate, the samples were imaged on a Talos F200C electron microscope (Thermo Fisher Scientific) operating at 200 kV with a Ceta CMOS camera. A control experiment with wild-type NET1ΔC that was prepared identically to the mutants confirmed that wild type NET1ΔC formed filaments under theses conditions, but no filaments were observed for the mutants.

To study the interaction of the NET1ΔC filaments with its dependence receptors, preformed filaments were mixed with 1 μM DCC or UNC5B in 20 mM NaH_2_PO_4_, 150 mM NaCl, pH 6.5 for at least 2 hours to form a complex. Freshly prepared complexes were then placed on glow-discharged carbon-coated copper grids and excessive liquid was removed using filter paper. A droplet of 5 nm Nanogold-Streptavidin (Nanoprobes, USA) was placed on the grid. After 10 min incubation at room temperature the grid was blotted, rinsed with water, and negatively stained with 0.75% uranyl acetate. Electron microscopy was performed with Talos F200C electron microscope (Thermo Fisher Scientific) operating at 200 kV with a Ceta CMOS camera at ×57,000 magnification.

### 2D alignment and averaging of NET1 filaments

The 237 negative stain images of the NET1ΔC filaments were converted from TIFF to MRC format using the EMAN2 software package^81^, and a subset of filaments were picked manually to generate 442 reference particles in 440 Å boxes. An initial 2D classification using the helical alignment programme in Relion^82^ assuming a 360 Å tube diameter and a helical rise of 7 Å yielded a low-resolution reference used for helical autopicking, which generated a total of 56235 particles in 704 Å boxes. These particles were sorted into 50 classes, with an assumed tube diameter of 380 Å and a rise of 7 Å. As all of the filaments were to some degree damaged or collapsed during staining, the offset search range was set to 220 Å to increase the probability of aligning intact sections of the filaments. Measurement of the tube dimensions from the resulting class averages was done using the Fiji installation of ImageJ^83^. The 234 full-length NET1 images were processed in the same manner, with 55,165 particles in 792 Å boxes aligned into 100 classes.

### Size exclusion chromatography coupled multiple angle light scattering (SEC-MALS)

Multiangle static light scattering (MALS) data were collected on a DAWN HELEOS II detector from Wyatt Technology (Santa Barbara, CA, USA) coupled to an ÄKTA pure FPLC system (Cytiva, Vancouver, Canada). Protein concentrations were recorded in-line with a 0.2 cm UV cell (Cytiva) at 280 nm and an Optilab T-rEX differential refractometer (Wyatt Technology). Both Wyatt detectors were operated with temperature control at 25 °C. For the measurements with the 24 ml Superose™ 6 Increase 10/300 GL size exclusion column (Cytiva^TM^), we injected 300 µl sample at 3–4 mg/ml and used a pump speed of 0.3 ml/min. If we used the LW-803 column (Shodex^TM^), we injected 150 µl sample at 4–5 mg/ml concentration and used a pump speed of 0.4 ml/min. Detector alignment, band broadening at detector normalisation were performed with 6 mg/ml bovine serum albumin (Millipore Sigma, Oakville, ON, Canada) in 50 mM tris, pH 7.5, 200 mM NaCl, the same buffer that was used for the sample. To prepare the sample mixture, 1.2 ml of NET1ΔC were concentrated at 1.0 mg/ml in above buffer to 400 µl (4.0 mg/ml) using an Amicon-4 concentrator (Millipore Sigma, Oakville, Canada) with 30 kDa molecular weight cutoff. 10 µl of HOs (dp6, dp8, dp10, dp12 or dp20) at 10 mg/ml were added immediately and incubated for 1–2 h. To remove aggregates, the sample was centrifuged for 5 min at 12,000 × *g* in a MiniSpin® tabletop centrifuge (Eppendorf, Mississauga, ON, Canada) immediately before injection. Data analysis was performed with ASTRA 7 (Wyatt) and the results were plotted with the Python library Matplotlib^84^.

### Analytical ultracentrifugation

NET1ΔC binding to HO-dp8 has been performed using the sedimentation velocity (SV) method in the analytical ultracentrifugation. Protein (at 1.0 mg/ml in buffer) and HO-dp8 (at 5.0 mg/ml in water) were dialysed overnight in separate Float-A-Lyzer® G2 dialysis devices (Repligen, Waltham, USA) with 0.5 Da molecular weight cut-off membranes in the same buffer container to equilibrate the buffer with the components. Protein and oligosaccharide were then mixed to various molar ratios with the protein concentration constant at 13 µM. The dialysed buffer was 50 mM tris, pH 7.5, 200 mM NaCl (*I* = 244 mM). The following protein/dp8 ratios were investigated: 1/0.25, 1/1, 1/2, 1/4, 1/8, 1/16, 1/32. SV experiments were performed using a ProteomeLab™ XL-I analytical ultracentrifuge and an An50Ti 8-cell rotor (Beckman Coulter Inc., Mississauga, ON)^29,85–87^. Standard 12 mm Epon double-sector cells were filled with 400 μl of buffer and 400 μl of sample. After a temperature equilibration of at least two hours at rest and under vacuum, the sedimenting samples were measured at rotors speeds of first 30,000 rpm and in a second experiment at 42,000 rpm for 24 h. Both, absorbance and interference data were collected. Two-dimensional distributions *c(s, f*_*r*_*)* of sedimentation coefficient *s* and frictional ratio *f*_*r*_ were calculated using the SEDFIT programme^88,89^ and plotted with GUSSI^90^.

### Mass photometry measurements

Prior to measurement, glass slides (24 × 50 mm #1.5 special, Menzel-Gläser) were reacted with 3-aminopropyl-triethoxysilane (APTES) to produce an amino-silane functionalized surface. Slides were plasma cleaned under O_2_, then dipped in a 5% APTES solution in acetone. Excess APTES was removed by rinsing in acetone. The slides were then baked at 110 °C before washing with isopropanol. 5 μM of either apo-NET1ΔC or NET1ΔC with equimolar HO-dp8 or HO-dp10 were incubated overnight in 50 mM tris pH 8.5, 200 mM NaCl at 4 °C. 0.2 μl of NET1ΔC-dp8 or NET1ΔC-dp10 were diluted in 9.8 μl of buffer directly on APTES functionalized glass slides in a 3 mm diameter 1 mm deep culture well gasket (Grace Bio-Labs), for a final concentration of 100 nM. Interferometric videos were taken on a prototype mass photometry system and processed as described^32^. Briefly, a 477 nm laser focussed to 1.5 μm was passed through a beam splitter and swept across the sample to generate an interferometric video of the buffer-glass interface. Frames were collected at 1000 Hz, and a differential video generated by subtracting each frame from the previous. In these videos, the interaction of the protein with the glass is clearly visible as a spot, where the intensity of the spot is directly proportional to the protein mass. Pixels were pre-binned 3 × 3 and frames were fivefold time averaged during acquisition, giving a final pixel size of 70.2 nm and a frequency of 200 Hz. During processing, videos were subjected to a further fivefold frame averaging. Masses were calculated via a calibration curve using alcohol dehydrogenase, β-amylase, and Protein A. For NET1ΔC w/o GAG, a total count of 8072 were measured with a monomer population of 92.99%. For the NETΔC-dp8 complex, 7192 counts revealed a monomer-dimer ratio of 86.37% to 12.39%. The NET1ΔC-dp10 complex was studied with 17075 total counts revealing a monomer-hexamer ratio of 79.45% to 3.38%.

### Size exclusion chromatography coupled small angle X-ray scattering (SEC-SAXS)

All SEC-SAXS data were collected at beamline B21, Diamond Light Source^91^ (Didcot, UK) that operates at a wavelength of at 1.0 Å a photon flux of ~10^12^ photons per second and features a fixed camera length of 4.014 m. It is equipped with an Agilent 1200 HPLC system.

Regarding NET1ΔC in presence of HO (experiment ID sm16028-7): Tag-free NET1ΔC was shipped to the beamline at 1.0 mg/ml in 50 mM tris, pH 7.5, 1.0 M NaCl. Using a PD-10 desalting column with Sephadex G-25 resin (Cytiva, Vancouver, Canada) the buffer was changed to 50 mM tris, pH 7.5, 0.2 M NaCl and the protein was then concentrated to 9.23 ± 0.09 mg/ml (186 ± 2 µM) using an Amicon-4 concentrator (MilliporeSigma, Etobicoke, Canada) with 30 kDa molecular weight cutoff. 20 µl of HO (*i.e*. dp4, dp6, dp8 or dp10 at 10 mg/ml in water) was then added to 100 µl protein solution and then let to incubate for 40–80 min before injection. A second series was let to incubate for 4–5 h before injection. We observed strong precipitate formation with HO-dp4, but less with HO-dp6. No precipitation occurred with HO-dp8 and HO-dp10. Precipitates were removed by centrifugation at 12,000 × *g* followed by filtration through a 0.1 µM Ultrafree-MC spin filter (MilliporeSigma, Etobicoke, Canada). 50 µl of sample were injected without further delay into the buffer equilibrated 4.6 ml Shodex KW 403-4 F size exclusion column from where the protein eluted through the diffraction flow cell at a rate of 0.16 ml/min. X-ray diffraction images were collected with 3 s exposure time. The software packages ScÅtter (http://www.bioisis.net/tutorials/9), Chromixs, or BioXTAS RAW were used to assess the elution profiles and to calculate the radius of gyration *R*_*g*_ for each frame in the sample elution region^92,93^. A series of 10–12 adjacent frames at the maximum of the elution peak were selected and averaged after buffer subtraction, yielding the scattering profiles for the 3D electron density reconstruction (Supplementary Figs. 16–19a, e). Guinier fits in the low scattering angle region of the scattering profile were performed with the programmes autorg and datrg from the ATSAS software package, yielding estimates for *R*_*g*_ and the scattering intensity at 0 angle *I(0)* (Supplementary Figs. 16–19b, c; Supplementary Table 5c, d)^94,95^. The indirect Fourier transforms (IFT) of the scattering profiles were calculated with the programme GNOM from the same package to obtain the pair distance distribution functions *P(r)* (Supplementary Figs. 16–19d). 3D electron density reconstruction was performed with the software package DENSS^30^. 93–108 reconstructions were generated per scattering profile (voxel size set to 5 Å and oversampling set to 5), which were aligned and averaged to yield an averaged electron density map. Using different random seeds to add uniformly distributed random noise to the averaged map prior to refinement, 25 final maps (Supplementary Figs. 16–19f, g, h) were refined from the averaged map, yielding a representative gallery of models that all fit the original scattering data. The model galleries are provided as an additional supplement.

Regarding NET1ΔC without HO (experiment ID sm22113-7): Tag-free chicken NET1ΔC was shipped injection-ready to the beamline at 5.8 mg/ml in 20 mM tris, pH 7.5, 500 mM NaCl (sample1) or 4.5 mg/ml in 50 mM tris, pH 7.5, 200 mM NaCl (sample11). 50 µl of sample was injected into the Shodex column equilibrated with the respective buffer (matched to the sample by overnight dialysis). For sample1, we used the Shodex KW403-4F column and for sample11 the Shodex KW404-4F column. The elution traces were assessed with the programme BioXTAS RAW which showed two overlapping peaks, indicating the presence of at least two different scattering components (Supplementary Figs. 10a, 11a). Using the evolving factor analysis (EFA) implemented in BioXTAS RAW, we deconvoluted the scattering signals of each component (Supplementary Figs. 10b–g, 11b–d)^96^. Even though not perfect, this method yields scattering profiles that have the signal contribution from the other components significantly reduced. Analysis and 3D electron density map reconstruction then progressed as described above. The Guinier analysis is shown in Supplementary Figs. 12–15a, b and Supplementary Table 5a, b; the *P(r)* distribution in Supplementary Figs. 12–15c, the fits to the scattering profile in Supplementary Figs. 12–15d and the depiction of a selected model from the gallery in Supplementary Figs. 12–15e, f, g.

### Calculation of hydrodynamic properties from DENSS electron density models

To calculate the hydrodynamic and geometric properties of the DENSS electron density models, we first cut the electron density volume to the support volume reported by DENSS by setting the electron density outside the support volume to 0. The support volume marks the upper limit of the particle volume. The support volume was then filled with the expected numbers of electrons (26376 for monomeric NET1ΔC, 52752 for dimeric NET1ΔC, 131800 for the NET1ΔC associated with heparin dp8/dp10 oligosaccharides). To estimate the real particle volume, we used the ATSAS suite to build bead models^97^. We first generated two unique cores (damstart.pdb), each averaged^98^ from a set of 20 different (random seed) DAMMMIF models^99^. The shape setting parameter suggested by DAMMIF was used for the 1^st^ core and the “unknown” shape setting parameter for the 2^nd^ core. From the 2 cores we calculated 4 DAMMIN models using different random seeds^100^. The average volume of the 4 DAMMIN models was used as target volume for the hydrodynamic calculations. If the DAMMIN volume was larger than the average DENSS support volume, we used the latter, instead. We then proceeded with the programme HYDROMIC to calculate the hydrodynamic and geometric properties of the DENSS electron density models^101^. For each dataset we had generated a set of 25 separate DENSS models (see SEC-SAXS section). For each set we provided a common electron density cut-off level to HYDROMIC, such that the resulting average volume of the entire set matched the target volume. We also calculated these properties for the averaged electron density map. The values for the averaged map and spread of the values for the refined models in brackets are given in Supplementary Table 5. We also included the properties of the DAMMIN bead models that were calculated using the programme HYDROPRO using the procedure we published earlier^87,102^. To accomplish the electron density volume calculations and manipulations we wrote Python scripts that were based on the *saxstats* module in DENSS^30^ and the *volumeInfo* and *volumeViewer* module from UCSF Chimera^68^. The scripts are available from the authors on request.

### Preparation of microcontact-printed substrates for neurite outgrowth assay

Neurite outgrowth from acutely prepared dorsal root ganglia (DRG) neurons was assayed using microcontact-printed substrates. Namely, on coverslips patterned with recombinant laminin-111 (Biolamina, Stockholm, Sweden) and the NET1ΔC variants below as the test substrate. Negative silicon masters used to create stamps for microcontact printing were provided by Dr. Siegmund Schroeter (Institute of Photonic Technology, Jena, Germany) from which PDMS stamps were cast, as described^103^. Protein printing inks were prepared containing 20 μg/ml recombinant laminin-111 or *Mus musculus* NET1ΔC wild type (NP_032770, aa: 24-457) or the double mutant (NP_032770, aa:1-457, R348A, R349A) in TBS containing 1 mM CaCl_2_. The printing ink also contained 2 µg/ml Alexa 555- conjugated goat anti-rabbit antibodies to provide a fluorescent marker for detecting printed regions. Coverslips for stamping were cleaned^33^ and activated with oxygen plasma for 1 minute immediately prior to stamping. Stamps covered in printing ink were incubated at 37 °C for 2 hours, then rinsed with ultrapure water and dried with nitrogen. Immediately after drying, the protein was printed from the stamp onto freshly activated glass coverslips. To create a cross-patterned substrate, the test protein was always printed on the coverslip first, the coverslip was then carefully removed from the stamp, rotated through 90 degree and then the recombinant laminin-111 was printed in bands at right angles to the test protein structures, creating a grid pattern. As a positive control, recombinant laminin-111 was printed in both directions. The printed substrates were then used within 1 hour of printing.

### Neuronal cell culture and neurite outgrowth assay

All mice were housed in a licensed animal housing facility at the Max Delbrück Centre, Germany and were euthanized before isolating the primary cells in strict compliance with protocols approved by the German federal authorities (specifically: Landesamt für Gesundheit und Soziales, State of Berlin animal experimentation committee). No experiments were performed with living mice. Male wild-type C57Bl/6 mice were obtained from Charles River, Sulzfeld, Germany at age 4 weeks and were housed for 2–4 days at controlled temperature with a 12 h light, 12 h dark cycle before euthanasia. Mice used in this study were naïve to any treatment or manipulation before euthanasia and sample preparation.

For each primary culture, dorsal root ganglia (DRG) were prepared acutely from a separate, single mouse. The DRG were collected in 1 ml PBS on ice, then treated for 30 minutes at 37 °C with 1 µg/ml collagenase IV in 1 ml PBS and finally for 5–20 minutes at 37 °C with 0.05% trypsin in 1 ml PBS. Cells were dissociated in 1 ml DMEM/F-12 by being passaged through a 20 G needle then collected, washed and finally resuspended in D-MEM/F-12 medium containing 10% horse serum. Cells (~60–120 µl of cell suspension per coverslip) were seeded on printed substrates. After 6 hours, an additional DRG medium was added to the coverslips. Cells were cultured for 24–48 hours at 37 °C in a Steri-Cult 200 incubator. No nerve growth factor or other neurotrophins were added to the medium. After neurite outgrowth over the recombinant laminin-111 was observed, cells were fixed using 4% PFA in PBS for 15 minutes at room temperature. Phase contrast images of the cells were obtained using an Axiovert 200, inverted light microscope (Zeiss, Jena, Germany). Fluorescence images of the labelled protein substrates were also obtained to visualise the protein pattern. Neurite outgrowth was assessed in two ways: First, the ratio of neurite outgrowth between test substrate and recombinant laminin-111 was calculated. Secondly, branching events where neurite branches had initiated on the test substrate, regardless of whether the neurite extended over the substrate or not, were counted and the number of branch points per unit length calculated. All data represents measurements obtained from cultures from at least four mice and at least five printed coverslips. The data were compared using either a Student’s *t* test (normally distributed data) or a Mann–Whitney test when data did not follow a normal distribution. The DRG were separated into parallel samples applied across all test substrates, thus no randomisation into separate sample groups was required. An exclusion criteria was established that any preparations in which no neurite outgrowth was observed would be removed from analysis (however, this situation did not arise in the course of the study).

#### *C. elegans* maintenance and strains

Nematode culture standard procedures were used for the culture, maintenance and genetic analysis of *C. elegans*^104^. Worms were grown at 20 °C unless otherwise noted. The N2 Bristol strain was used as the standard wild type strain. All other strains used in experiments were outcrossed at least 4 times to the N2 Bristol control strain. All strains used in this study are listed in Supplementary Table 7. Two independent Crispr/Cas strains were generated for the *unc-6* heparin binding deficient mutants *unc-6(syb2327) & unc-6(syb2328)* (C,D: NP_509165: C: R378, H379A and D: H403A, R404A, K405A; obtained from Suny biotech).

### Distal tip cell (DTC) migration scoring

Late L4 or young adult hermaphrodites without eggs grown at 25 °C without starvation were mounted on a 5% agarose pad in M9 buffer containing 5 mM sodium azide. Their gonad shapes were observed under a Zeiss axiovision microscope. For each genotype 20 animals (experimental replicates) were scored with six or three biological replicates (*N* = 3 or 6). If less than 10 animals fulfilled the chosen criteria, the scoring was disregarded. Observed DTC migration defects of anterior and posterior gonad arms were divided in five classes (class 1: including typical *unc-6* and *unc-5* DTC migration defects, class 2: including phase 3 DTC migration defects, class 3: including phase 2 DTC migration defects, class 2: including phase 1 DTC migration defects and class 5: including mild or such which had another nature than DTC migration defects. Data from independent counts were subsequently analysed with GrapPad Prism. Images were taken with an Axio Imager Z1 Zeiss axiovision microscope. For the statistical analysis, the total observed defect percentage was used. DTC migration scoring was analysed with an ordinary one-way ANOVA Tukey multiple comparisons test after testing for normal distribution. Total sample sizes for each genotype were: *lon-2(e678)* *N* = 120; *lon-2(e678) unc-6(syb2327)* *N* = 120; *lon-2(e678) unc-6(syb2328)* *N* = 120; *unc-52(e699)* *N* = 120; *unc-52(e699);unc-6(syb2327)* *N* = 120; *unc-52(e699);unc-6(syb2328)* *N* = 120; *sdn-1(zh20)* *N* = 120; *sdn-1(zh20) unc-6(syb2327)* *N* = 120; *sdn-1(zh20) unc-6(syb2328)* *N* = 120; *unc-40(n324)* *N* = 60; *unc-40(n324);unc-6(syb2327)* *N* = 60; *unc-40(n324);unc-6(syb2328)* *N* = 60; *unc-6(ev400) N* = *120; unc-6(syb2327)* *N* = 120; *unc-6(syb2328)* *N* = 120; *unc-40(n324);lon-(e678)* *N* = 60; *unc-5(e152)* *N* = 60; *unc-5(e152);unc-6(syb2327)* *N* = 60; *unc-5(e152);unc-6(syb2328)* *N* = 60;*unc-5(e152);lon-(e678)* *N* = 60; wild type N2 *N* = > 300.

### PVM/AVM & PLM/ALM neuron scoring

Late L4 or young adult hermaphrodites without eggs grown at 25 °C without starvation under ad libitum bacteria were mounted on a 5% agarose pad in M9 buffer containing 50 mM sodium azide. PVM/AVM & PLM/ALM neuron were observed and imaged under a Zeiss axiovision microscope. For each genotype at least three biological replicates with a minimum number of 20 animals (experimental replicate) were scored. Data from independent scorings were subsequently analysed with GrapPad Prism. Images were taken with an Axio Imager Z1 Zeiss axiovision microscope. For the statistical analysis two-way ANOVA Tukey multiple comparisons test after testing for normal distribution was used. For most of the genotypes, a total of 60 worms were scored and used for statistical analysis. Analysed variables were the genotype and neuron typ. The computed results indicate that 9% of total variance results from interaction of the variables (*F* = 4.07, DFn = 3, DFd = 54 *P* value is <0.0001). If there is no interaction overall, there is a <0.01% chance that such an interaction will be observed by chance for an experiment of this size. Furthermore, the computed results indicate that the genotype accounts for 74% of the total variance (*F* = 98.13. DFn = 18, DFd = 200, *P* value <0.0001). The neuron type affect was determined to account for 9% of the total variance (F = 71.88. DFn = 3, DFd = 200 *P* value <0.0001).

### Vulva scoring

Late L4 hermaphrodites grown at 25 °C without starvation under ad libitum bacteria were mounted on a 5% agarose pad in M9 buffer containing 50 mM sodium azide. Vulvas were observed and imaged under a Zeiss axiovision microscope. For each genotype at least three biological replicates with a minimum number of 20 animals (experimental replicates) were scored. If less than 10 animals fulfilled the chosen criteria, the scoring was discarded. Data from independent scorings were subsequently analysed with GrapPad Prism. For the statistical analysis, an ordinary one-way ANOVA Tukey multiple comparisons test after testing for normal distribution was used. Total sample sizes for each genotype were: wild type N2 *N* ≥ 300; *unc-6(syb2327)* *N* = 89; *unc-6(syb2328)* *N* = 110; *unc-6(ev400)* *N* = 35; *lon-2(e678)* *N* = 61; *lon-2(e678) unc-6(syb2327)* *N* = 64; *lon-2(e678) unc-6(syb2328)* *N* = 93; *unc-52(e699)* *N* = 101; *unc-52(e699);unc-6(syb2327)* *N* = 102; *unc-52(e699);unc-6(syb2328)* *N* = 109; *sdn-1(zh20)* *N* = 111; *sdn-1(zh20) unc-6(syb2327)* *N* = 106; *sdn-1(zh20) unc-6(syb2328)* *N* = 99; *unc-40(n324)* *N* = 58; *unc-40(n324);unc-6(syb2327)* *N* = 56; *unc-40(n324);unc-6(syb2328)* *N* = 43; *unc-5(e152)* *N* = 59;*unc-5(e152);unc-6(syb2327)* *N* = 60; *unc-5(e152);unc-6(syb2328)* *N* = 60.

### Puncta scoring of NET1/UNC-6

Outcrossed *ghIs9(unc-6p::Venus::unc-6)* and *lon-2(e678) ghls9(unc-6p::Venus::unc-6)* strains were grown at 20 °C on NGM plates. Gravid adult hermaphrodites were transferred onto fresh NGM plates and let lay eggs for 4 hours. After removing adult hermaphrodites, NGM plates were grown at 20 °C and 25 °C. At day 1 and day 9, 20–25 hermaphrodites were mounted on 5% agarose pads in M9 buffer containing 100 mM sodium azide. Z stack images of the head region were obtained by using a high magnification (×40 objective) on a Leica SP8-DLS microscope. For each condition three biological replicates with at least 11 worms (experimental replicates) were imaged. The number and size of observed fluorescent spots were analysed using IMARIS. Prior to the analysis images were processed applying background subtraction, gaussian filter and adjustment of threshold using IMARIS. For the spot analysis the spots function of IMARIS was used setting following parameters: spots bigger than 1.5 mm, using a quality filter within the limits 4.5 and 12 and an additional intensity mean filter within the limits 10 and 35. For the statistical analysis GraphPad prism was used. After testing for normal distribution, a Kruskal-Wallis Dunn’s multiple comparisons test was applied to the number of puncta. A Kruskal–Wallis Dunn’s multiple comparisons test was applied to analyse the size of puncta. For most of the tested conditions a minimum of 36 hermaphrodites were imaged and used for statistical analysis.

### Reporting summary

Further information on research design is available in the Nature Portfolio Reporting Summary linked to this article.

## Supplementary information

Supplementary Information

Description of Additional Supplementary Files

Supplementary Data 1

Supplementary Data 2

Supplementary Data 3

Supplementary Movie 1

Supplementary Movie 2

Supplementary Movie 3

Reporting Summary

## Data Availability

The X-ray crystallography datasets and structural models from this study have been deposited in the Protein Data Bank under accession codes 7LRF and 7LER. Other coordinates and structure factors used as a reference in this study are available in the Protein Data Bank under accession codes 4OVE and 3IRJ. The DENSS 3D electron density reconstructions from SEC-SAXS were deposited to the Small Angle Scattering Biological Data Bank (SASBDB) under accession codes SASDRJ2, SASDRK2, SASDRL2, SASDRM2, SASDRN2, SASDRP2, SASDRQ2, SASDRR2 and additional scattering data are included with the supplement. Protein-ligand model and simulation trajectory files of the molecular dynamics simulation are available on the Centre for Open Science (OSF) database. SEC-MALS and sedimentation velocity raw data sets are available from the authors on request. The python scripts we wrote for electron density volume calculations are available from the authors on request. All other data generated in this study are included in the published article and supplement. Source data are provided in this paper.

## Source data

Source Data
