# Supplementary material for: The dynamic nature of netrin-1 and the structural basis for glycosaminoglycan fragment-induced filament formation
Source: Nat Commun. 2023 Mar 3;14:1226. doi: 10.1038/s41467-023-36692-w (PMC9984387; doi:10.1038/s41467-023-36692-w)
Supplement: Supplementary file 1 — Supplementary Information [file 41467_2023_36692_MOESM1_ESM.pdf]

The Dynamic Nature of Netrin-1: The Structural Basis for Glycosaminoglycan Fragment induced Filament Formation

Markus Meier[1#], Monika Gupta[1#], Serife Akgül[2,3#], Matthew McDougall[1#], Thomas Imhof[2], Denise Nikodemus[4], Raphael Reuten[5,6], Aniel Moya-Torres[1], Vu To[1], Fraser Ferens[1], Fabian Heide[1], Gay Pauline Padilla-Meier[1], Philipp Kukura[7], Wenming Huang[3], Birgit Gerisch[3], Matthias Mörgelin[8], Kate Poole[9], Adam Antebi[3,10*], Manuel Koch[2,11,12*], & Jörg Stetefeld[1*]

*****Corresponding authors: aantebi@age.mpg.de, manuel.koch@uni-koeln.de, jorg.stetefeld@umanitoba.ca

# These authors contributed equally

[1]Department of Chemistry, University of Manitoba, Canada

[2]Center for Biochemistry II, Faculty of Medicine and University Hospital Cologne, University of Cologne, 50931, Cologne, Germany.

[3]Max Planck Institute for Biology of Ageing, Cologne, Germany

[4]Faculty of Biology, Institute of Biology II, Albert Ludwigs University of Freiburg, Germany

[5]Institute of Experimental and Clinical Pharmacology and Toxicology, Medical Faculty, University of Freiburg, Freiburg, Germany.

[6]Department of Obsterics and Gynecology, Medical Center, University of Freiburg, Freiburg, Germany.

[7]Physical and Theoretical Chemistry Laboratory, Department of Chemistry, University of Oxford, South Parks Road, Oxford OX1 3QZ, UK.

[8]Colzyx AB, Lund, Sweden

[9]Max Delbrück Center for Molecular Medicine, Robert Roessle Str 10, Berlin-Buch, Germany

Current address: EMBL Australia Node in Single Molecule Science, School of Medical Sciences, Faculty of Medicine, University of New South Wales, Sydney, NSW, Australia

[10]Cologne Excellence Cluster on Cellular Stress Responses in Aging Associated Diseases, University of Cologne, Cologne 50931, Germany

[11]Institute for Dental Research and Oral Musculoskeletal Biology, Faculty of Medicine and University Hospital Cologne, University of Cologne, 50931, Cologne, Germany.

[12]Center for Molecular Medicine Cologne, Faculty of Medicine and University Hospital Cologne, University of Cologne, 50931, Cologne, Germany.

Abbreviations

dp: Degree of polymerization

HO: Heparin oligosaccharide

NET1: Netrin-1

NET4: Netrin-4

NET5: Netrin-5

SOS: Sucrose octasulfate

UNC-6: Netrin unc-6 / uncoordinated protein 6

Supplementary Tables

Supplementary Table 1: Data collection and refinement statistics

	NET1ΔC with SOS<br>pdb: 7LRF	NET1ΔC filaments<br>pdb: 7LER
	**Data collection**	
**Space group**	P 2_1 2_1 2_1	P 6_5
**Cell dimensions**		
***a*, *b*, *c* (Å)**	75.105, 80.152, 241.702	196.69, 196,69, 476.33
**α, β, γ (°)**	90, 90, 90	90, 90, 120
**Resolution (Å)**	47.08 - 3.21 (3.325 - 3.21)*	49.17 - 5.99 (6.40 - 5.99)
**R_{sym} or R_{merge}**	0.188 (1.061)	0.190 (1.476)
**$I / \Sigma I$**	9.8 (2.0)	8.0 (1.8)
**Completeness (%)**	99.47 (98.67)	99.5 (98.2)
**Redundancy**	8.3 (8.4)	11.1 (10.9)
**$CC_{1/2}$**	0.994 (0.634)	0.997 (0.777)
**Refinement**		
**No. reflections**	24519 (2375)	26028 (4633)
**R_{value} / R_{free}**	0.2273 / 0.2799	0.3044 / 0.3681
**No. atoms**	6727	26405
**Protein**	6301	25469
**Ligand/ion**	422	936
***B*-factors**		
**Protein**	88.84	305.08
**Ligand/ion**	154.22	290.38
**R.m.s. deviations**		po
**Bond lengths (Å)**	0.011	0.014
**Bond angles (°)**	1.10	1.11
**Ramachandran Statistics**		
**Favoured (%)**	85.42	89.37
**Disallowed (%)**	1.94	0.34

*Values in parentheses are for highest-resolution shell.

Supplementary Table 2: Masses from SEC-MALS

	**Mass (kDa)**		
**Column**	Superose™ 6 increase 10/300 GL	Superose™ 6 increase 10/300 GL	Shodex LW-803
Date	*20171118*	*201811211*	*20200703*
**NET1ΔC**	65.2 ± 0.1<br>85.1 ± 0.2	73.9 ± 0.3<br>92.0 ± 0.3	59.5 ± 0.4<br>80.0 ± 0.3
Date	*20171108/1*	*20171108/2*	
**NET1ΔC + HO-dp6**	72.5 ± 0.2<br>93.5 ± 0.3	71.5 ± 0.2<br>86.0 ± 0.2	n/a
Date	*20190130*		*20200703*
**NET1ΔC + HO-dp8**	73.9 ± 0.3<br>235.0 ± 0.4	n/a	154.0 ± 0.7<br>234.9 ± 0.7
**NET1ΔC + HO-dp10**	110.2 ± 0.4<br>267.5 ± 0.4	n/a	177.5 ± 1.0<br>286.0 ± 0.8
**NET1ΔC + HO-dp12**	139 ± 6<br>278 ± 4	n/a	n/a
**NET1ΔC + HO-dp20**	360.1 ± 0.5	n/a	n/a

The uncertainties represent standard uncertainties of the measurement (statistical consistency of the data). The actual uncertainties of the masses are larger (~20%). Masses shown were determined from a narrow region (0.4 ml) at the elution peak centre. In all experiments with a double peak, the one with the larger mass was the dominant peak (see **Fig. 4a**).

Supplementary Table 3: Buffer and molecule parameters used in the sedimentation velocity analysis

**Tag-free *Gallus gallus* NET1ΔC**			
**Property**	***Value***	***Temperature***	***Source***
**Partial specific volume *ν***	0.70998 cm^3/g	20 °C	Sednterp[1]
**Molecular mass[§] M_w**	52367.5 Da	20 °C	Sednterp[1]
**Hydration**	0.390089 g/g	20 °C	Sednterp[1]

[§]Ammonium sulfate treated tag-free *Gallus gallus* NET1ΔC with four common core pentasaccharides (6x β-D-N-Acetyl glucosamine (GlcNAc), 9x β-D-Mannose (Man), 3x glycosidic linkage)

**HO-dp8 (Iduron # HO08)**			
**Property**	***Value***	***Temperature***	***Source***
**Partial specific volume *ν***	0.467 cm^3/g	20 °C	Pavlov et al.[2]
**Mean molecular mass M_w**	~2400 Da	20 °C	Iduron

**0.05 M tris, pH 7.5, 0.20 M NaCl**			
**Density *ρ***	1.007900 g/cm^3	20 °C	Sednterp
**Viscosity *η***	0.0103573 P	20 °C	Sednterp

Supplementary Table 4: Masses from sedimentation velocity

**Population**	**NET1ΔC oligomeric state**	**Mass (kDa)**	**Peak width (kDa)**
**NET1**	Monomers	50	±13
**[NET1]^{2}**	Dimers	109	±23
**[NET1]^{3}**	Trimers	163	±26
**[NET1]^{5}**	Tetramer-Pentamer	273	±48
**[NET1]$^{6-8}$**	6 – 8	399	±65
**[NET1]$^{8-10}$**	8 - 10	543	±52
**[NET1]$^{10-12}$**	10 - 12	729	±49

Measured masses of the NET1ΔC populations in solution associated to HO-dp8. The shown masses and peak widths were averaged from the *c(s, M)* distributions of two independent sedimentation velocity experiments (Supplementary Fig. 9b). The most likely oligomeric state of each population is indicated. For larger species, a significant number of HO-dp8 may be bound, making the protein oligomeric state assignment ambiguous.

Supplementary Table 5: Hydrodynamic and geometric properties of SEC-SAXS electron density models (DENSS) and bead models (DAMMIN)

Supplementary Table 5a

Parameter	Deconvoluted NET1ΔC monomer	Deconvoluted NET1ΔC monomer	Method
**Experiment ID**	sm22113-7/sample11	sm22113-7/sample1	
**SEC column**	Shodex KW404-4F	Shodex KW403-4F	
**Buffer**	50 mM tris, pH 7.5, 200 mM NaCl	20 mM tris, pH 7.5, 500 mM NaCl	
**Number of DENSS models**	24	23	DENSS[3]
**Number of DAMMIN models**	20	20	DAMMIN[4]
**Hydrodynamic radius $R_{h\ 20°C,\ w}$ (nm)**	4.05 [4.33 - 4.73]	3.75 [3.98 - 4.26]	HYDROMIC[5] (DENSS)
**Hydrodynamic radius $R_{h\ 20°C,\ w}$ (nm)**	[4.16 - 4.31]	[4.09 - 4.20]	HYDROPRO[6] (DAMMIN)
**Sedimentation coefficient† $s_{20°C,\ w}$ (S)**	3.31 [2.83 - 3.10]	3.58 [3.15 - 3.37]	HYDROMIC[5] (DENSS)
**Sedimentation coefficient† $s_{20°C,\ w}$ (S)**	[3.11 - 3.23]	[3.20 - 3.28]	HYDROPRO[6] (DAMMIN)
**Radius of gyration R_g (nm)**	4.09 ± 0.04	3.98 ± 0.03	BioXTAS RAW[7]
**Radius of gyration R_g (nm)**	3.93 [3.79- 3.88]	3.89 [3.69 - 3.79]	HYDROMIC[5] [DENSS]
**Radius of gyration R_g (nm)**	[4.31 - 4.33]	[4.39 - 4.41]	DAMMIN[4]
**Extrapolated scattering intensity at 0 angle *I(0)***	0.708 ± 0.003	0.9316 ± 0.003	BioXTAS RAW[7]
**Longest dimension D_{max} (nm)**	14.11	14.83	DATCLASS[8]
**Longest dimension D_{max} (nm)**	15.73 [15.26 - 16.58]	14.51 [14.49 - 15.88]	HYDROMIC[5] (DENSS)
**Longest dimension D_{max} (nm)**	[18.15 - 18.84]	[18.18 - 18.88]	DAMMIN[4]
**Corr. Porod Volume V_p (nm^3)**	85.4	81.3	BioXTAS RAW[7, 9]
**Volume *V* (nm^3)**	[124 - 126]	[82 - 84]	DAMMIN[4]
**Reproduced volume†† (nm^3)**	125 [112 - 134]	83 [78 - 87]	HYDROMIC[5]
**Support Volume††† (nm^3)**	[294 - 342]	[248 - 309]	DENSS[3]
**Corrected†††† molecular mass *M* (kDa)**	55.6 [52.6 - 58.8]	49.9 [48.3 - 53.8]	PRIMUS - bayesian[10]
**Formula mass† (kDa)**	52.3675	52.3675	SEDNTERP[1]
**$\chi 2$ of fit**	[1.51 - 3.94]	[6.60 - 14.52]	DENSS[3]
**Resolution of averaged map (Å)**	39.5	39.4	DENSS[3]

Numbers in bracket refer to range of values found within the models. The number without brackets refers to the averaged DENSS electron density map.

† Based on formula mass, includes 3 core pentasaccharides. The mass is an input parameter to HYDROMIC.

†† Based on average DAMMIN volume.

††† Always larger than real particle volume.

†††† $\frac{M_{\text{primus}} * 0.7425 \text{cm}^3/\text{g}}{\bar{\nu}}$ with $\bar{\nu}$ = 0.70998 cm^3/g. Uncertainty is given as 95% credibility interval.

Supplementary Table 5b

Parameter	Deconvoluted NET1ΔC dimer	Deconvoluted NET1ΔC dimer	Method
**Experiment ID**	sm22113-7/sample11	sm22113-7/sample1	
**SEC column**	Shodex KW404-4F	Shodex KW403-4F	
**Buffer**	50 mM tris, pH 7.5, 200 mM NaCl	20 mM tris, pH 7.5, 500 mM NaCl	
**Number of DENSS models**	35	24	DENSS[3]
**Number of DAMMIN models**	20	20	DAMMIN[4]
**Hydrodynamic radius $R_{h\ 20°C,\ w}$ (nm)**	5.11 [5.29 - 5.79]	4.95 [5.31 - 5.64]	HYDROMIC[5] (DENSS)
**Hydrodynamic radius $R_{h\ 20°C,\ w}$ (nm)**	[5.02 - 5.13]	[5.05 - 5.21]	HYDROPRO[6] (DAMMIN)
**Sedimentation coefficient† $s_{20°C,\ w}$ (S)**	5.25 [4.64 - 5.07]	5.42 [4.76 - 5.06]	HYDROMIC[5] (DENSS)
**Sedimentation coefficient† $s_{20°C,\ w}$ (S)**	[5.22 -5.34]	[5.15 - 5.31]	HYDROPRO[6] (DAMMIN)
**Radius of gyration R_g (nm)**	5.78 ± 0.07	5.86 ± 0.06	BioXTAS RAW[7]
**Radius of gyration R_g (nm)**	6.02 [5.43 - 5.60]	5.46 [5.58 - 5.71]	HYDROMIC[5] [DENSS]
**Radius of gyration R_g (nm)**	[5.99 - 6.00]	[6.04 - 6.05]	DAMMIN[4]
**Extrapolated scattering intensity at 0 angle *I(0)***	1.616 ± 0.008	1.058 ± 0.005	BioXTAS RAW[7]
**Longest dimension D_{max} (nm)**	19.56	20.14	DATCLASS[8]
**Longest dimension D_{max} (nm)**	21.42 [20.76 - 24.43]	21.39 [21.25 - 22.77]	HYDROMIC[5] (DENSS)
**Longest dimension D_{max} (nm)**	[21.94 - 22.49]	[20.81 - 21.35]	DAMMIN[4]
**Corr. Porod Volume V_p (nm^3)**	150	154	BioXTAS RAW[7, 9]
**Volume *V* (nm^3)**	[165 - 168]	[168 - 171]	DAMMIN[4]
**Reproduced volume†† (nm^3)**	166 [148 - 179]	170 [156 - 181]	HYDROMIC[5]
**Support Volume††† (nm^3)**	[647 - 801]	[447 - 546]	DENSS[3]
**Corrected†††† molecular mass *M* (kDa)**	79.8 [76.7 - 90.9]	75.7 [66.0 - 80.9]	PRIMUS - bayesian[10]
**Formula mass† (kDa)**	104.735	104.735	SEDNTERP[1]
**$\chi 2$ of fit**	[0.381 - 2.501]	[1.468 - 3.713]	DENSS[3]
**Resolution of averaged map (Å)**	35.8	46.3	DENSS[3]

Numbers in bracket refer to range of values found within the models. The number without brackets refers to the averaged DENSS electron density map.

† Based on formula mass, includes 3 core pentasaccharides. The mass is an input parameter to HYDROMIC.

†† Based on average DAMMIN volume.

††† Always larger than real particle volume.

†††† $\frac{M_{\text{primus}} * 0.7425 \text{cm}^3/\text{g}}{\bar{\text{v}}}$ with $\bar{v}$ = 0.70998 cm^3/g. Uncertainty is given as 95% credibility interval.

Supplementary Table 5c

Parameter	NET1ΔC with dp8	NET1ΔC with dp8	Method
**Experiment ID**	sm16028-7/379532	sm16028-7/379539	
**SEC column**	Shodex KW403-4F	Shodex KW403-4F	
**Number of DENSS models**	23	24	DENSS[3]
**Number of DAMMIN models**	4	4	DAMMIN[4]
**Buffer**	50 mM tris, pH 7.5, 200 mM NaCl	50 mM tris, pH 7.5, 200 mM NaCl	
**Hydrodynamic radius $R_{h\ 20°C,\ w}$ (nm)**	6.63 [6.94 - 7.45]	6.78 [6.99 - 7.40]	HYDROMIC[5] (DENSS)
**Hydrodynamic radius $R_{h\ 20°C,\ w}$ (nm)**	[7.09 - 7.29]	[7.31 - 7.38]	HYDROPRO[6] (DAMMIN)
**Sedimentation coefficient† $s_{20°C,\ w}$ (S)**	6.07 [5.40 - 5.80] (trimer)<br>8.10 [7.20 - 7.74] (tetramer)<br>10.12 [9.00 - 9.67] (pentamer)<br>12.14 [10.80 - 11.60] (hexamer)<br>14.17 [12.60 - 13.54] (heptamer)<br>16.19 [14.39 - 15.47] (octamer)	5.93 [5.44 - 5.75] (trimer)<br>7.91 [7.25 - 7.67] (tetramer)<br>9.89 [9.07 - 9.59] (pentamer)<br>11.87 [10.88 - 11.51] (hexamer)<br>13.85 [12.69 - 13.42] (heptamer)<br>15.82 [14.51 - 15.34] (octamer)	HYDROMIC[5] (DENSS)
**Sedimentation coefficient† $s_{20°C,\ w}$ (S)**	[5.51 - 5.67] (trimer)<br>[7.35 - 7.57] (tetramer)<br>[9.19 - 9.46] (pentamer)<br>[11.03 - 11.35] (hexamer)<br>[12.87 - 13.24] (heptamer)<br>[14.71 - 15.13] (octamer)	[5.45 - 5.50] (trimer)<br>[7.27 - 7.34] (tetramer)<br>[9.09 - 9.17] (pentamer)<br>[10.91 - 11.01] (hexamer)<br>[12.72 - 12.84] (heptamer)<br>[14.54 -14.68] (octamer)	HYDROPRO[6] (DAMMIN)
**Radius of gyration R_g (nm)**	6.46 ± 0.07	6.43 ± 0.07	AUTORG[11]
**Radius of gyration R_g (nm)**	6.69 [6.22 - 6.31]	6.11 [6.22 - 6.36]	HYDROMIC[5] [DENSS]
**Radius of gyration R_g (nm)**	[6.58 - 6.59]	[6.57]	DAMMIN[4]
**Extrapolated scattering intensity at 0 angle *I(0)***	0.1930 ± 0.0003	0.2490 ± 0.0004	AUTORG[11]
**Longest dimension D_{max} (nm)**	21.53	20.7	DATCLASS[8]
**Longest dimension D_{max} (nm)**	23.25 [22.55 - 27.17]	23.59 [22.80 - 24.53]	HYDROMIC[5] (DENSS)
**Longest dimension D_{max} (nm)**	[23.71 - 24.48]	[22.75 - 23.30]	DAMMIN[4]
**Corr. Porod Volume V_p (nm^3)**	416	409	BioXTAS RAW[7, 9]
**Volume *V* (nm^3)**	[463 - 478]	[704 - 715]	DAMMIN[4]
**Reproduced volume†† (nm^3)**	470 [443 - 499[	712 [569 - 671]	HYDROMIC[5] (DENSS)
**Support Volume††† (nm^3)**	[1161608 - 1362902]	[1008867 - 1261762]	DENSS[3]
**Corrected†††† molecular mass *M* (kDa)**	254 [231 - 390]	333 [231 - 390]	PRIMUS - bayesian[10]
**Formula mass† (kDa)**	157.1025 (trimer)<br>209.4700 (tetramer)<br>261.8375 (pentamer)<br>314.2050 (hexamer)<br>366.5725 (heptamer)<br>418.9400 (octamer)	157.1025 (trimer)<br>209.4700 (tetramer)<br>261.8375 (pentamer)<br>314.2050 (hexamer)<br>366.5725 (heptamer)<br>418.9400 (octamer)	SEDNTERP[1]
**$\chi 2$ of fit**	[0.04- 0.07]	[0.05- 0.16]	DENSS[3]
**Resolution of averaged map (Å)**	74.0	62.7	DENSS[3]

Numbers in bracket refer to range of values found within the models. The number without brackets refers to the averaged DENSS electron density map.

† Based on formula mass, includes 3 core pentasaccharides. The mass is an input parameter to HYDROMIC.

†† Based on average DAMMIN volume.

††† Always larger than real particle volume.

†††† $\frac{M_{\mathrm{primus}} * 0.7425\mathrm{cm}^3/\mathrm{g}}{\bar{v}}$ with $\bar{v}$ = 0.70998 cm^3/g. Uncertainty is given as 95% credibility interval.

Supplementary Table 5d

Parameter	NET1ΔC with dp10	NET1ΔC with dp10	Method
**Experiment ID**	sm16028-7/379533	sm16028-7/379540	
**SEC column**	Shodex KW403-4F	Shodex KW403-4F	
**Number of DENSS models**	25	21	DENSS[3]
**Number of DAMMIN models**	4	4	DAMMIN[4]
**Buffer**	50 mM tris, pH 7.5, 200 mM NaCl	50 mM tris, pH 7.5, 200 mM NaCl	
**Hydrodynamic radius $R_{h\ 20°C,\ w}$ (nm)**	6.67 [6.92 - 7.57]	6.95 [7.08 - 7.63]	HYDROMIC[5] (DENSS)
**Hydrodynamic radius $R_{h\ 20°C,\ w}$ (nm)**	[7.08 - 7.19]	[7.27 - 7.38]	HYDROPRO[6] (DAMMIN)
**Sedimentation coefficient† $s_{20°C,\ w}$ (S)**	6.04 [5.32 - 5.81] (trimer)<br>8.05 [7.09 - 7.75] (tetramer)<br>10.06 [8.86 - 9.69] (pentamer)<br>12.08 [10.63 - 11.62] (hexamer)<br>14.09 [12.40 - 13.56] (heptamer)<br>16.10 [14.18 - 15.50] (octamer)	5.79 [5.28 -5.68] (trimer)<br>7.72 [7.04 - 7.57] (tetramer)<br>9.65 [8.79 - 9.46] (pentamer)<br>11.59 [10.55 - 11.36] (hexamer)<br>13.52 [12.31 - 13.25] (heptamer)<br>15.45 [14.07 - 15.14] (octamer)	HYDROMIC[5] (DENSS)
**Sedimentation coefficient† $s_{20°C,\ w}$ (S)**	[5.60 - 5.68] (trimer)<br>[7.46 - 7.57] (tetramer)<br>[9.33 - 9.47] (pentamer)<br>[11.19 - 11.36] (hexamer)<br>[13.06 - 13.25] (heptamer)<br>[14.93 - 15.15] (octamer)	[5.45 - 5.53] (trimer)<br>[7.27 - 7.37] (tetramer)<br>[9.09 - 9.22] (pentamer)<br>[10.90 - 11.06] (hexamer)<br>[12.72 - 12.90] (heptamer)<br>[14.54 - 14.75] (octamer)	HYDROPRO[6] (DAMMIN)
**Radius of gyration R_g (nm)**	6.43 ± 0.06	6.47 ± 0.04	AUTORG[11]
**Radius of gyration R_g (nm)**	6.97 [6.24 - 6.33]	6.12 [6.24 - 6.30]	HYDROMIC[5] [DENSS]
**Radius of gyration R_g (nm)**	[6.56]	[6.56]	DAMMIN[4]
**Extrapolated scattering intensity at 0 angle *I(0)***	0.2340 ± 0.0004	0.3610 ± 0.0005	AUTORG[11]
**Longest dimension D_{max} (nm)**	21.3	21.8	DATCLASS[8]
**Longest dimension D_{max} (nm)**	24.38 [22.23 - 25.77]	22.58 [22.38 - 25.24]	HYDROMIC[5] (DENSS)
**Longest dimension D_{max} (nm)**	[22.25- 22.50]	[22.12 - 22.83]	DAMMIN[4]
**Corr. Porod Volume V_p (nm^3)**	429	437	BioXTAS RAW[7, 9]
**Volume *V* (nm^3)**	[445 - 450]	[757 - 775]	DAMMIN[4]
**Reproduced volume†† (nm^3)**	447 [424 - 496]	766 [603 - 711]	HYDROMIC[5] (DENSS)
**Support Volume††† (nm^3)**	[1111685 - 1259630]	[1095212 - 1303757]	DENSS[3]
**Corrected†††† molecular mass *M* (kDa)**	333 [231 - 391]	333 [231 - 391]	PRIMUS - bayesian[10]
**Formula mass† (kDa)**	157.1025 (trimer)<br>209.4700 (tetramer)<br>261.8375 (pentamer)<br>314.2050 (hexamer)<br>366.5725 (heptamer)<br>418.9400 (octamer)	157.1025 (trimer)<br>209.4700 (tetramer)<br>261.8375 (pentamer)<br>314.2050 (hexamer)<br>366.5725 (heptamer)<br>418.9400 (octamer)	SEDNTERP[1]
**$\chi 2$ of fit**	[0.09 - 0.16]	[0.05 - 0.10]	DENSS[3]
**Resolution of averaged map (Å)**	77.6	63.3	DENSS[3]

Numbers in bracket refer to range of values found within the models. The number without brackets refers to the averaged DENSS electron density map.

† Based on formula mass, includes 3 core pentasaccharides. The mass is an input parameter to HYDROMIC.

†† Based on average DAMMIN volume.

††† Always larger than real particle volume.

†††† $\frac{M_{primus} * 0.7425 cm^3/g}{\bar{v}}$ with $\bar{v}$ = 0.70998 cm^3/g. Uncertainty is given as 95% credibility interval.

Supplementary Table 6: Amino acid sequences and settings to generate conservation plot

```
Background frequencies: bgfreq.txt
Blosum substitution matrix:   blosum.txt
Chosen segment:   None
Colors:     {'050000': 'DE', '000000': 'RKH'}
Hobohm identity threshold:    0.63
Lines per page/picture: 2
Logo type:  Shannon
Minimum stack width fraction: 0.5
Position number of first stack:     341
Requested formats:      PDF
Resolution: 640x480
Sequence weighting type:      None
Show Ends:  False
Show X-axis:      True
Show Y-axis:      True
Show Y-axis label:      True
Show fineprint:   True
Stacks per line:  33
Tic interval of the x-axis:   0
Title:
Unit type:  Bits
Vertical x-axis numbers:      False
Weight on Prior:  0.0
Y-axis range:     [0.0, 0.0]
```

```
>sp|P34710|UNC6_CAEEL Netrin unc-6 OS=Caenorhabditis elegans OX=6239 GN=unc-6 PE=1 SV=1
VACNCNQHAKRCRFDAELFRLSGNRSGGVCLNCRHNTAGRNCHLCKPGFVRDTSLPMTHRKACKSC

>sp|O95631|NET1_HUMAN Netrin-1 OS=Homo sapiens OX=9606 GN=NTN1 PE=1 SV=2
VACNCNLHARRCRFNMELYKLSGRKSGGVCLNCRHNTAGRHCHYCKEGYYRDMGKPITHRKACKAC

>sp|O09118|NET1_MOUSE Netrin-1 OS=Mus musculus OX=10090 GN=Ntn1 PE=1 SV=3
VACNCNLHARRCRFNMELYKLSGRKSGGVCLNCRHNTAGRHCHYCKEGFYRDMGKPITHRKACKAC

>sp|Q90922|NET1_CHICK Netrin-1 OS=Gallus gallus OX=9031 GN=NTN1 PE=1 SV=1
VACNCNLHARRCRFNMELYKLSGRKSGGVCLNCRHNTAGRHCHYCKEGFYRDLSKPISHRKACKEC

>tr|O42140|O42140_DANRE Netrin-1 OS=Danio rerio OX=7955 GN=ntn1a PE=2 SV=1
VACNCNLHARRCRFNMELYKLSGRKSGGVCLNCRHNTAGRHCHYCKEGYYRDMSKPISHRKACKAC

>tr|B0CM04|B0CM04_DANRE Netrin-1 (Fragment) OS=Danio rerio OX=7955 GN=ntn1b PE=1 SV=2
VACHCNLHARRCRFNMELYKLSGRRSGGVCLNCRHNTAGRHCHYCKEGYYRDMSKAISHRRACKAC

>sp|Q2HXW4|NET1_PIG Netrin-1 OS=Sus scrofa OX=9823 GN=NTN1 PE=2 SV=1
VACNCNLHARRCRFNMELYKLSGRKSGGVCLNCRHNTAGRHCHYCKEGYFRDLGKPITHRKACKAC

>sp|Q924Z9|NET1_RAT Netrin-1 OS=Rattus norvegicus OX=10116 GN=Ntn1 PE=1 SV=1
VACNCNLHARRCRFNMELYKLSGRKSGGVCLNCRHNTAGRHCHYCKEGFYRDMGKPITHRKACKAC

>tr|F1N0C7|F1N0C7_BOVIN Netrin-1 OS=Bos taurus OX=9913 GN=NTN1 PE=4 SV=2
VACNCNLHARRCRFNMELYKLSGRKSGGVCLNCRHNTAGRHCHYCKEGYYRDLGKPITHRKACKAC
```

>tr|F6QTJ6|F6QTJ6_HORSE Netrin-1 OS=Equus caballus OX=9796 GN=NTN1 PE=4 SV=3
VACNCNLHARRCRFNMELYKLSGRKSGGVCLNCRHNTAGRHCHYCKEGYYRDMGKPITHRKACKAC

>tr|A0A3Q2Q7P5|A0A3Q2Q7P5_FUNHE Netrin-1 OS=Fundulus heteroclitus OX=8078 PE=4 SV=1
VACHCNLHARRCRFNMELFKLSGRKSGGVCLNCRHNTAGRHCHYCKEGYYRDLSKPISHRKACKAC

>tr|A0A2Y9Q6I1|A0A2Y9Q6I1_DELLE Netrin-1 OS=Delphinapterus leucas OX=9749 GN=NTN1 PE=4 SV=1
VACNCNLHARRCRFNMELYKLSGRKSGGVCLNCRHNTAGRHCHYCKEGYYRDLGKPITHRKACKAC

>tr|W5NXF2|W5NXF2_SHEEP Netrin-1 OS=Ovis aries OX=9940 PE=4 SV=1
CSCNCNLHARRCRFNMELYKLSGRKSGGVCLNCRHNTAGRHCHYCKEGYYRDLGKPITHRKACKAC

>tr|O57339|O57339_XENLA Netrin-1 (Fragment) OS=Xenopus laevis OX=8355 GN=Netrin-1 PE=2 SV=1
VACNCNLHARRCRFNMELFKLSGRRSGGVCLNCRHNTAGRHCHYCKEGYYRDMTKAITHRKACKAC

>tr|H2QC90|H2QC90_PANTR Netrin-1 OS=Pan troglodytes OX=9598 GN=NTN1 PE=2 SV=2
VACNCNLHARRCRFNMELYKLSGRKSGGVCLNCRHNTAGRHCHYCKEGYYRDMGKPITHRKACKAC

>tr|A0A3P8RME6|A0A3P8RME6_AMPPE Netrin-1 OS=Amphiprion percula OX=161767 GN=NTN1 PE=4 SV=1
VACNCNLHARRCRFNMELYKLSGRKSGGVCLNCRHNTAGRHCHYCKEGYYRDLSKPISHRKACKAC

>tr|G3SUN1|G3SUN1_LOXAF Netrin-1 OS=Loxodonta africana OX=9785 GN=NTN1 PE=4 SV=1
VACNCNLHARRCRFNMELYKLSGRKSGGVCLNCRHNTAGRHCHYCKEGYYRDMGKPITHRKACKAC

>tr|A0A090LSV4|A0A090LSV4_STRRB Netrin-1a OS=Strongyloides ratti OX=34506 GN=SRAE_X000060400 PE=4 SV=1
VACNCNLHARKCRFNAELYRLSGNKSGGICINCRHNTAGRNCHYCKPGYYRDNTKAITHRRACKAC

>tr|A0A6P6KPE7|A0A6P6KPE7_CARAU Netrin-1 OS=Carassius auratus OX=7957 GN=LOC113053406 PE=4 SV=1
VACHCNLHARRCRFNMELYKLSGRRSGGVCLNCRHNTAGRHCHYCKEGYYRDMTKAISHRRACKAC

>tr|F7ELZ6|F7ELZ6_XENTR Netrin-1 OS=Xenopus tropicalis OX=8364 GN=LOC100494394 PE=4 SV=4
KRCRCHNHAHSCHFDLSVWLSSGRTSGGVCENCQHHTEGDRCQRCQPGYYRDPKEPMASPAACKEC

>tr|A0A1S2ZPN6|A0A1S2ZPN6_ERIEU Netrin-1 OS=Erinaceus europaeus OX=9365 GN=NTN1 PE=4 SV=1
VACNCNLHARRCRFNMELYKLSGRKSGGVCLNCRHNTAGRHCHYCKEGYYRDLGKPITHRKACKAC

>tr|A0A6I9INJ8|A0A6I9INJ8_VICPA Netrin-1 OS=Vicugna pacos OX=30538 GN=NTN1 PE=4 SV=1
VACNCNLHARRCRFNMELYKLSGRKSGGVCLNCRHNTAGRHCHYCKEGYYRDLGKPITHRKACKAC

Supplementary Table 7: All strains generated and used in this study

Name	Genotype	Source
**AA5202**	unc-6(syb2327)	This study/SunyBiotech
**AA5203**	unc-6(syb2328)	This study/SunyBiotech
**NW434**	unc-6(ev400) X	This study/CGC
**AA5349**	N2;unc-52(e669)	This study/CGC
**AA1495**	N2;lon-2(e678)	This study/CGC
**AH205**	N2;sdn-1(zh20) X.	This study/CGC
**AA5350**	N2; unc-5(e152)	This study/CGC
**AA5351**	N2; unc-40(n342)	This study/CGC
**AA5427**	unc-6(syb2327) X; unc-40(n342)	This study
**AA5426**	unc-6(syb2328) X; unc-40(n342)	This study
**AA5424**	unc-6(syb2328) X; unc-5(e152)	This study
**AA5425**	unc-6(syb2327) X; unc-5(e152)	This study
**AA5420**	unc-6(syb2328) X; lon-2(e678)	This study
**AA5421**	unc-6(syb2327) X; lon-2(e678)	This study
**AA5422**	unc-6(syb2328) X; sdn-1(zh20)	This study
**AA5423**	unc-6(syb2327) X; sdn-1(zh20)	This study
**AA5418**	unc-6(syb2328) X; unc-52(e669)	This study
**AA5419**	unc-6(syb2327) X; unc-52(e669)	This study
**AA5495**	lon-2(e678);unc-5(e152) IV	This study
**AA5496**	lon-2(e678);unc-40(n324) I.	This study
**AA5362**	N2; zdIs5 [mec-4p::GFP + lin-15(+)]	This study/CGC
**AA5440**	sdn-1; zdIs5 [mec-4p::GFP + lin-15(+)]	This study
**AA5441**	unc-52;zdIs5 [mec-4p::GFP + lin-15(+)]	This study
**AA5361**	lon-2(e678); zdIs5 [mec-4p::GFP + lin-15(+)]	This study
**AA5438**	unc-40; zdIs5 [mec-4p::GFP + lin-15(+)]	This study
**AA5439**	unc-5; zdIs5 [mec-4p::GFP + lin-15(+)]	This study
**AA5366**	unc-6(syb2327); zdIs5 [mec-4p::GFP + lin-15(+)]	This study
**AA5365**	unc-6(syb2328); zdIs5 [mec-4p::GFP + lin-15(+)]	This study
**AA5364**	unc-6(ev400); zdIs5 [mec-4p::GFP + lin-15(+)]	This study
**AA5437**	unc-6(27);unc-40(n342); zdIs5 [mec-4p::GFP + lin-15(+)]	This study
**AA5436**	unc-6(28);unc-40(n342); zdIs5 [mec-4p::GFP + lin-15(+)]	This study
**AA5434**	unc-6(28);unc-5(e152); zdIs5 [mec-4p::GFP + lin-15(+)]	This study
**AA5435**	unc-6(27);unc-5(e152) ; zdIs5 [mec-4p::GFP + lin-15(+)]	This study
**AA5431**	unc-6(27);lon-2(e678); zdIs5 [mec-4p::GFP + lin-15(+)]	This study
**AA5430**	unc-6(28);lon-2(e678);zdIs5 [mec-4p::GFP + lin-15(+)]	This study
**AA5432**	unc-6(28);sdn-1(zh20); zdIs5 [mec-4p::GFP + lin-15(+)]	This study
**AA5433**	unc-6(27);sdn-1(zh20); zdIs5 [mec-4p::GFP + lin-15(+)]	This study
**AA5428**	unc-6(28);unc-52(e669); zdIs5 [mec-4p::GFP + lin-15(+)]	This study
**AA5429**	unc-6(27);unc-52(e669);zdIs5 [mec-4p::GFP + lin-15(+)]	This study
**YC42**	ghIs9(unc-6p::venus::unc-6) IV.	Yoshio Goshima, Japan
**AA5468**	lon-2(e678);ghIs9(unc-6p::venus::unc-6) IV.	This study

Supplementary Figures

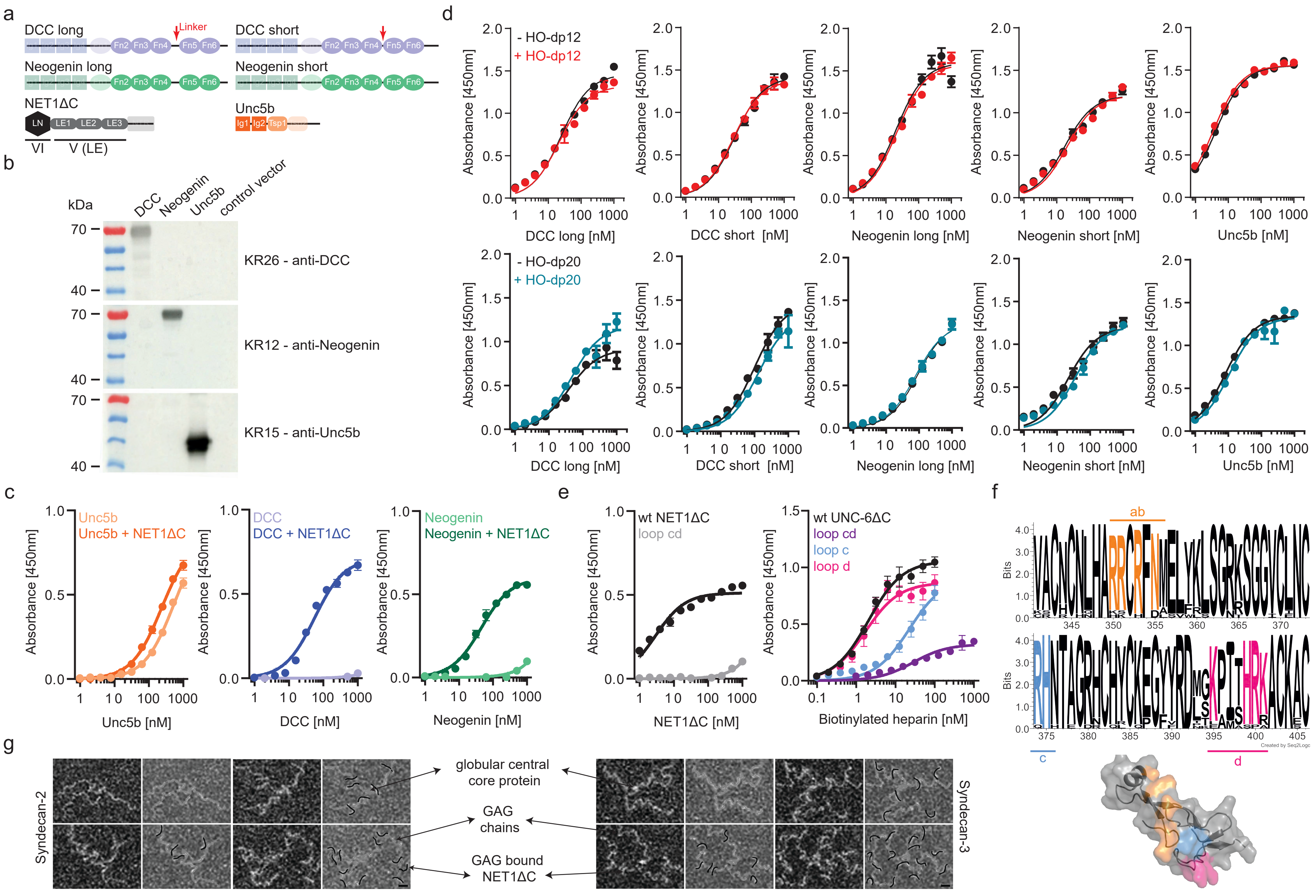

Supplementary Figure 1

(**a**) Schematic drawing of the protein domain structures used for binding assays. Linker regions in the neogenin long and the DCC long constructs are marked by a red arrow. Abbreviation: LN, laminin-like domain; LE1 - LE3, Laminin-type epidermal growth factor subdomains; NTR, Netrin-like domain; Ig1 - Ig4, immunoglobulin domains; FN1 - FN6, fibronectin type III domains; TSP1 - TSP2, thrombospondin type 1 domains. (**b**) Validation of the home-made polyclonal antibodies by Western blot analysis using HEK-293 cell supernatants overexpressing the DCC, neogenin and UNC5B ectodomains. Each of the antibody specifically detects the proper ectodomain. (**c**) ELISA style binding assays of the DCC, neogenin or UNC5B ectodomains in the presence and absence of NET1ΔC were assessed for binding to immobilized glypican-3 (1:1 ratio, max concentration 1000 nM). Error bars represent the mean ±SD of N = 3 technical repeats. (**d**) Binding of the DCC, neogenin or UNC5B ectodomains to immobilized NET1ΔC was measured in presence and absence of short (dp12) and medium length (dp20) HOs. Error bars represent the mean ±SD of N = 3 technical repeats. (**e**) Left side: ELISA style binding assay of wild type NET1ΔC and NET1ΔC loop cd against immobilized glypican-3. Error bars represent the mean ±SD of N = 3 technical repeats. Right side: ELISA style binding assay of biotinylated porcine heparin against immobilized wild type UNC-6ΔC, UNC-6ΔC loop c, UNC-6ΔC loop d and UNC-6ΔC loop cd. Error bars represent the mean ±SD of N = 3 technical repeats. (**f**) Sequence conservation logo[12] of NET1 LE-2 subdomain based on amino acid sequences of 22 organisms including invertebrates. Mutated amino acid patches ab (orange), c (blue), d (magenta) are colored. Same color code was used to map the mutation sites onto the structure of NET1 LE-2 subdomain below (PDB ID: 4PLM). (**g**) Negatively stained transmission electron micrographs of NET1ΔC and syndecan-2/3 interactions. Variable numbers of NET1ΔC molecules are attached to different locations along the GAG chains. Left panel: syndecan-2; right panel: syndecan-3. Each panel is divided into a left side (original electron micrographs) and a right side (interpretation in pseudo colours. Grey: syndecan-2/3; black: NET1ΔC. The scale bar represents 20 nm.

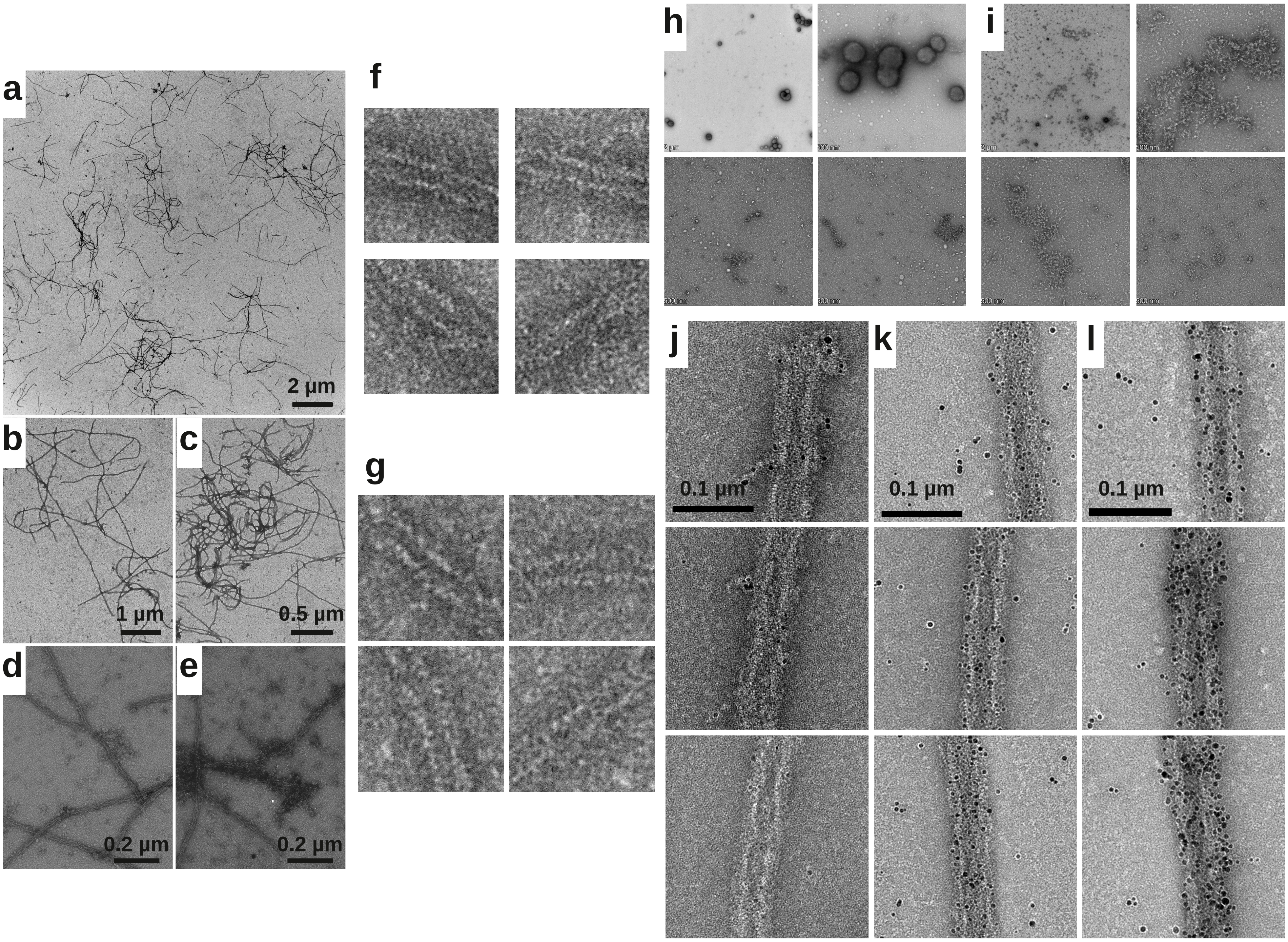

Supplementary Figure 2

(**a - e**) Overview of filament architecture. A field of NET1ΔC filaments is shown in (**a**) with the scale bar of 2 µm. (**b & d**) NET1ΔC filaments are shown at different magnifications with scale bars of 1 µm (**b**) and 0.2 µm (**d**). NET1-full length filaments (**c & e**) are shown at different magnifications with a scale bar of 0.5 µm (**c**) and 0.2 µm (**e**), respectively. (**f - g**) Representative picked particles used to generate the aligned 2-D class averages of NET1ΔC (**f**) and full-length NET1 (**g**). Negative stain images of NET1ΔC loop ab (**h**), and NET1ΔC loop cd (**i**) mutants show that filament formation is not detectable. (**j - l**) TEM images of gold labelled dependence receptor domains of UNC5 (**j**), DCC^{long} (**k**) and DCC^{short} (**l**) in complex with NET1ΔC filaments. To determine location of receptors bound to NET1ΔC filaments, Nanogold-Streptavidin (black dots) was added to the complex to bind to the Twin-Strep-tag® on DCC and UNC5. The scale bars represent 0.1 µm.

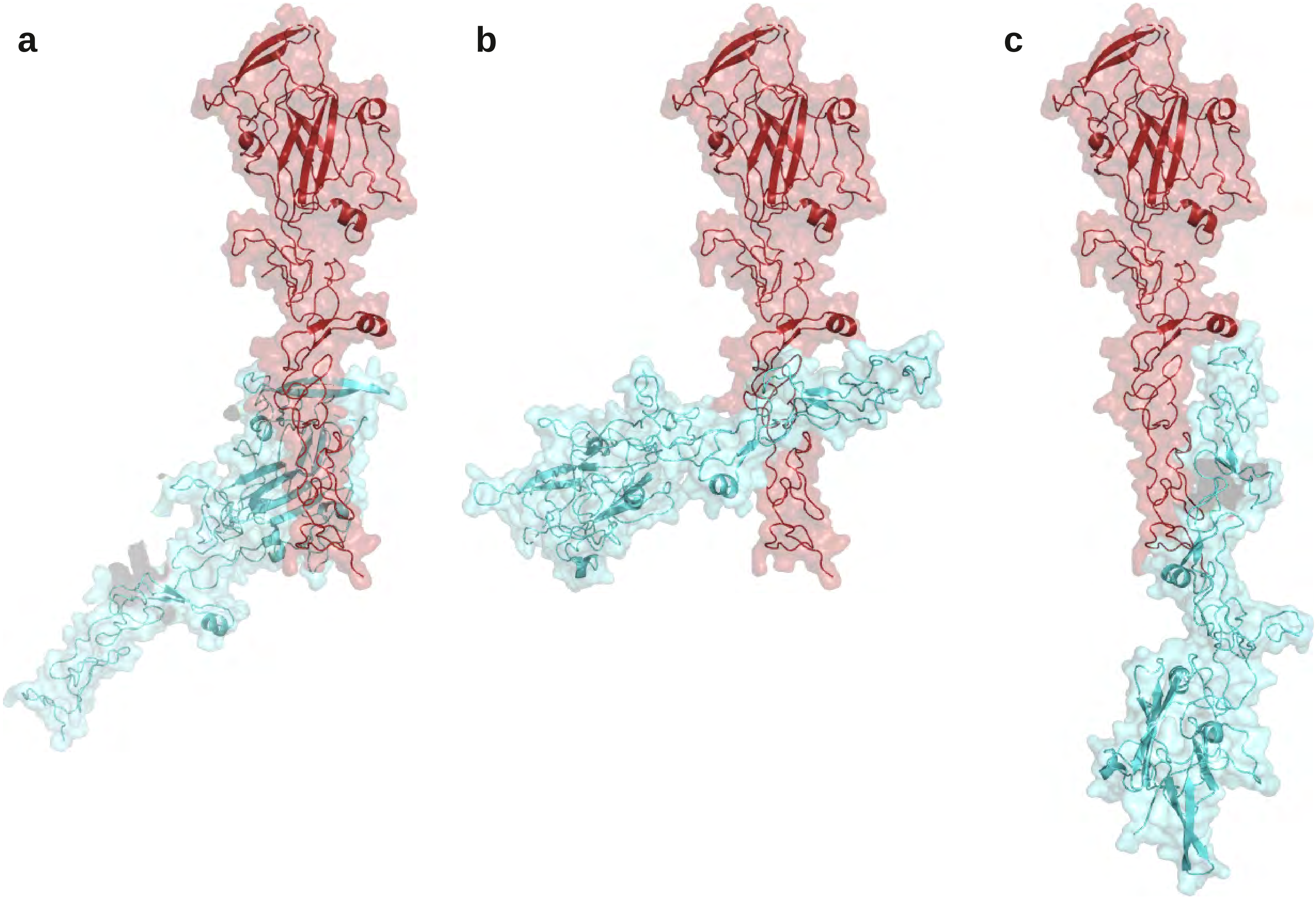

Supplement Figure 3

Analysis of the low resolution crystal structure of the NET1ΔC helix reveals three unique interaction interfaces. (**a**) Interactions between the LE-2 subdomain of NET1ΔC (red) and the top of the LN domain of the adjacent NET1ΔC (teal) and between the LE-2 subdomains of both NET1ΔC molecules form the core of the helical motif. (**b**) Though reminiscent of the published cross-shaped NET1ΔC dimer (PDB 4PLN), the second interaction observed here occurs on the opposite face of the LE-2 subdomain. (**c**) The third observed interaction occurs throughout the LE-2 and LE-3 subdomains, and is also observed in several other NET1ΔC structures (PDB 4OVE, 7LRF). Whereas interactions shown in **a** and **b** are essential for helix formation, the third interface results in NET1ΔC "spikes" projecting from the helical core, giving rise to the observed density outside the tubular walls in the aligned negative stain images.

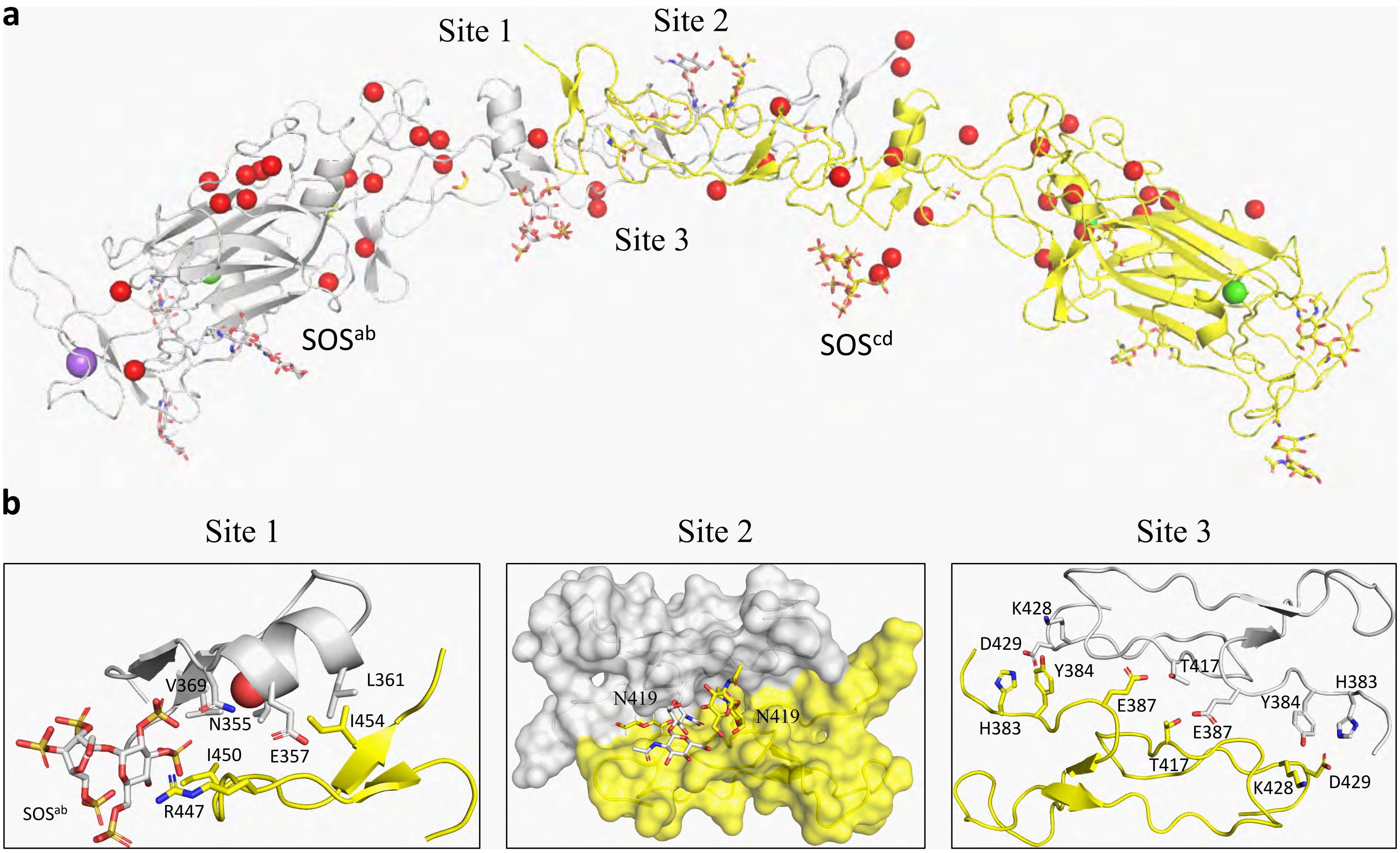

Supplementary Figure 4

(**a**) The antiparallel NET1ΔC dimer is banana-shaped and spans a maximum dimension of ~20.0 nm, in contrast to the NET1ΔC monomer which has a length of ~13.5 nm. Cartoons of monomer A and B are shown in silver and yellow, respectively. Both calcium ions are shown as green spheres, the sodium ion is magenta. Water molecules are shown as red balls. All N-glycans and both sucrose octasulphate (SOS) moieties, named SOS^{ab} and SOS^{cd}, are presented as sticks and balls. Examination of the buried surface area revealed that only three contact points stabilize the dimeric NET1ΔC assembly, named sites 1 to 3. (**b**) The site 1 interaction uses the α-helix in loop b of LE-2 to contact the antiparallel β-sheet of LE-3, whereas site 2 is mediated via carbohydrate moieties of N419 glycan linkages. Site 3 is an electrostatic crossover at the centre of gravity between T417 and E387 acting like a hinge-like element.

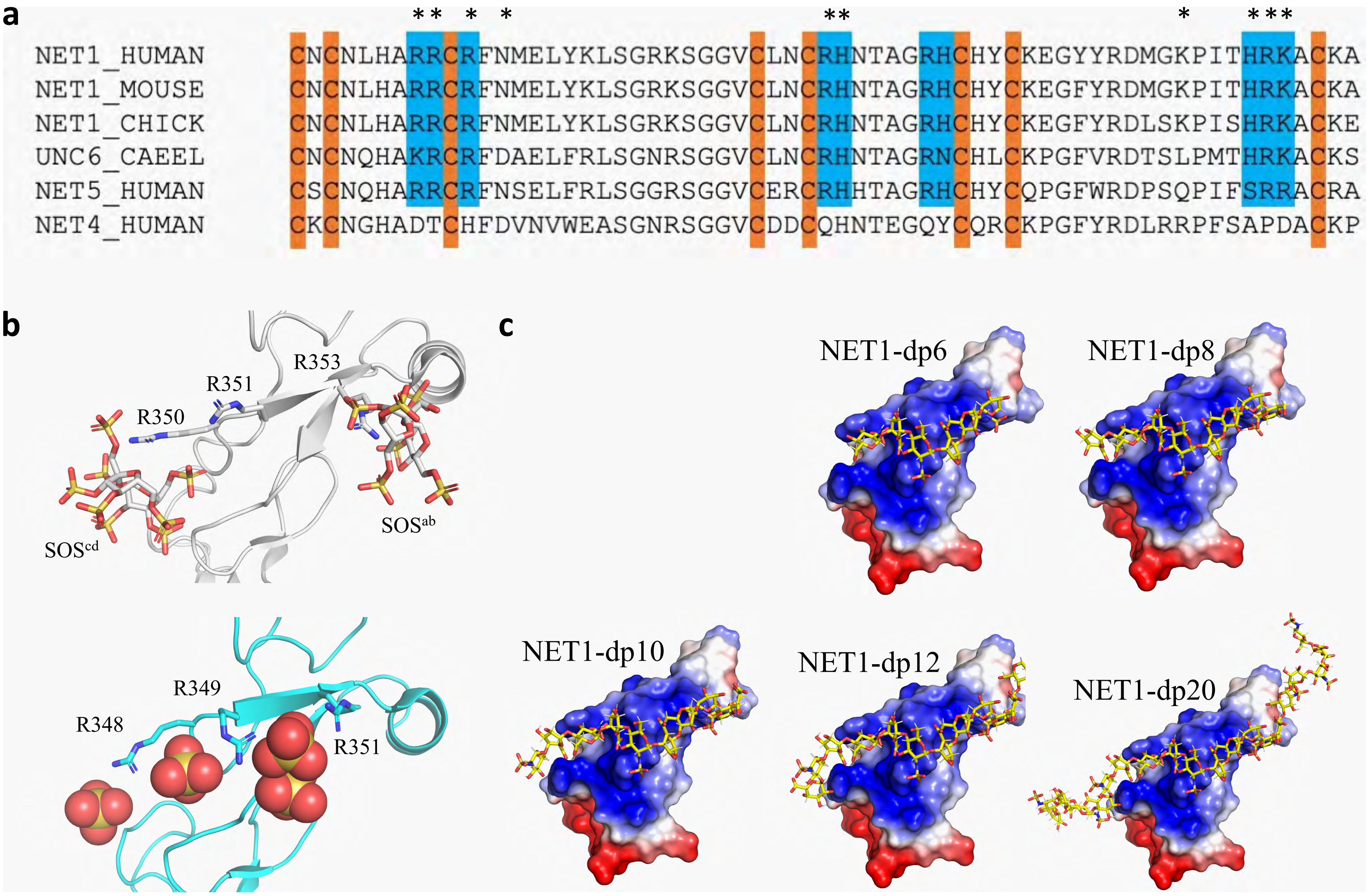

Supplementary Figure 5

(**a**) Sequence alignment and structural comparison of different LE-2 subdomains of NET1, NET4, NET5 and UNC-6. The LE-2 subdomain of *Homo sapiens* NET1 (NP_004813), *Mus musculus* NET1 (NP_032770), *Gallus gallus* NET1 (NP_990750), *Caenorhabditis elegans* UNC-6 (NP_509165) as well as human netrin-5 (NP_ 665806) and human netrin-4 (NP_ 067052) were aligned. The eight cysteine residues in each subdomain LE-2 are disulfide linked in the order 1 - 3, 2 - 4, 5 - 6, and 7 - 8, creating the four loop segments a - d. The residues studied in the mutation analysis are marked by *. (**b**) Detailed structural comparison of the Cardin-Weintraub motif in NET1. Top view represents NET1ΔC-SOS (pdb-code: 7LRF). The bottom cartoon represents the sulfate binding motif in 4URT[13]. Individual arginine residues are labelled and the sulfate moieties are shown as spheres. (**c**) The electrostatic potential is displayed as color gradient from red (*-8 k_b T/e_c*) to blue (*+8 k_b T/e_c*). Structural models represent different NET1ΔC-HO complexes based upon the NET1ΔC-SOS structure.

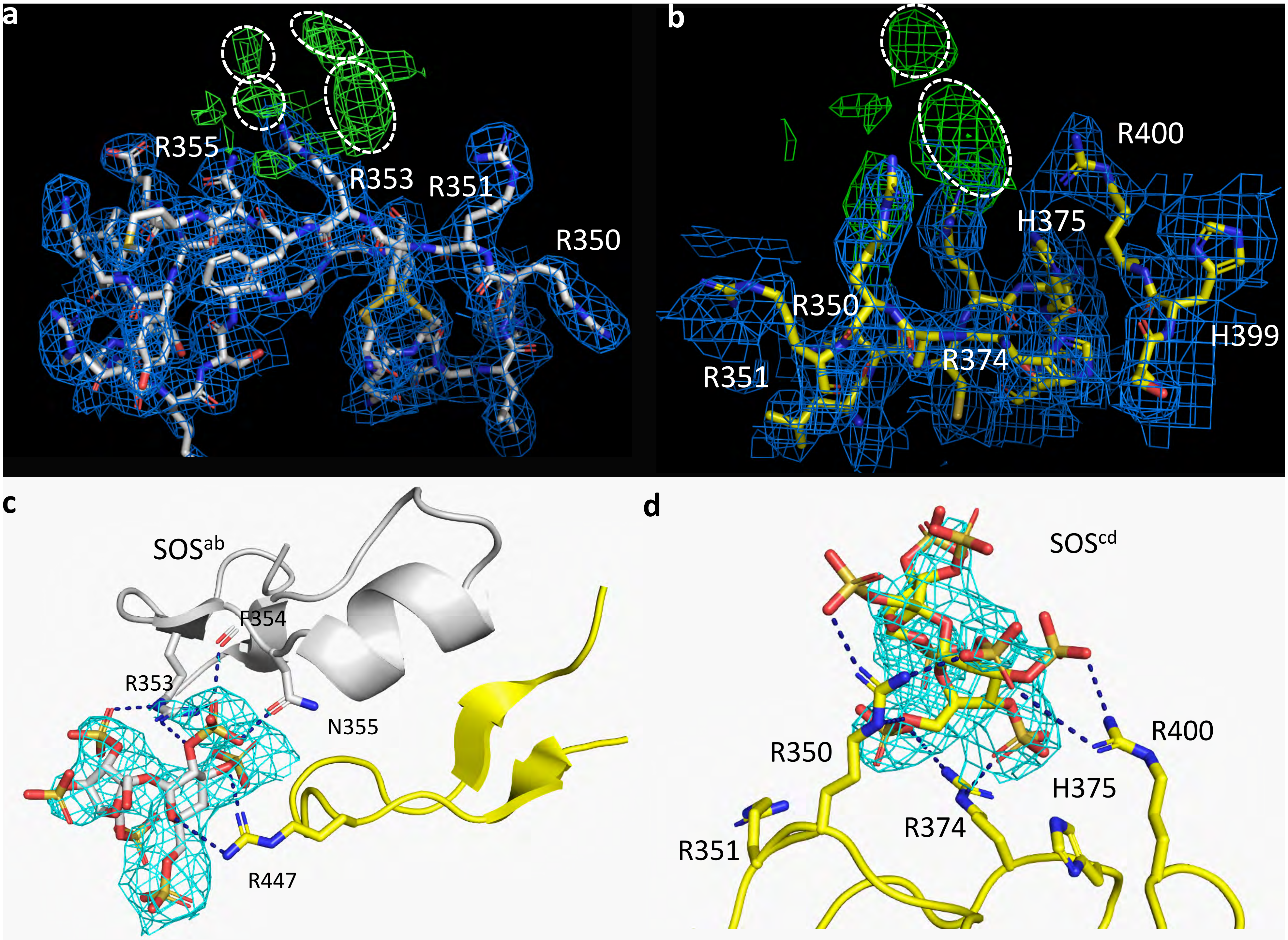

Supplementary Figure 6

(**a**) and (**b**) Models of NET1ΔC-sucrose octasulphate crystal structures SOS^{ab} (left) and SOS^{cd} (right) built into the SigmaA-weighted electron density maps from refinement of the NET1ΔC-apo model after molecular replacement in Phaser[14, 15]. The 2mFo-DFc maps (blue) and mFo-DFc difference maps (green) are contoured at 1 σ and ±3 σ respectively. Unmodelled features in the electron density maps are highlighted by white dashed lines. Asterisks mark the density into which sulphate groups of the SOS ligands were initially placed, and onto which a full five-six membered SOS molecule was superposed. In the subsequent refinement stages the R_{value}/R_{free} dropped from 26.8/33.1 to 22.7/27.9, respectively. (**c**) and (**d**) Electron density map for the 3.2 Å crystal structure of NET1ΔC in complex with SOS^{ab} and SOS^{cd}. The Cyan mesh represents the SigmaA weighted 2mFo-DFc electron density map contoured at 1 σ, after the final round of structure refinement in Phenix[14, 15]. The carbon backbone for GAG binding segments are colored in silver (SOS^{ab}) and yellow (SOS^{cd}), whereas other atoms are highlighted as follows: nitrogen, blue; oxygen, orange; sulphur, red. Distances less than 3.8 Å between donor - acceptor hydrogen pairings are indicated by black dashed lines. The sidechains of the Cardin-Weintraub motif residues R350 and R353 are suitably positioned to form electrostatic interactions with the negatively charged sulphate groups of both SOS ligands. Remarkably, whereas SOS^{ab} is involved in salt bridges with R353 and R447 (neighbouring monomer), SOS^{cd} is attached via salt bridges with R350 (loop a segment), R374 (loop c) and R400 (loop d). Whereas H375 is embedded in a cleft between both R374 and R400, it is not involved in electrostatic interactions. R351 is disordered and clear density is not visible for the sidechain.

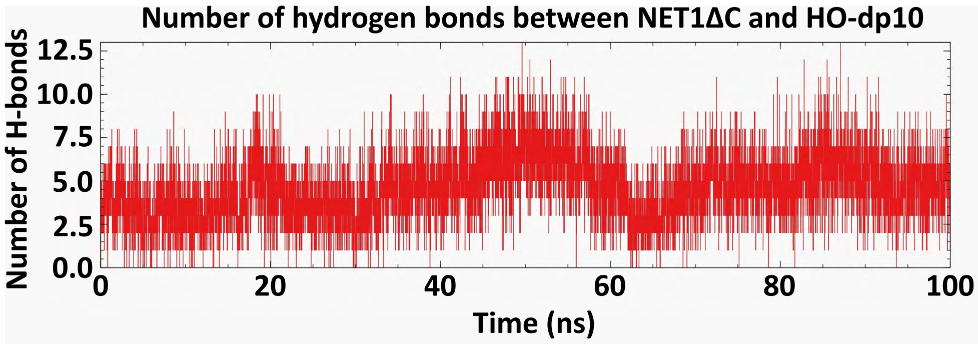

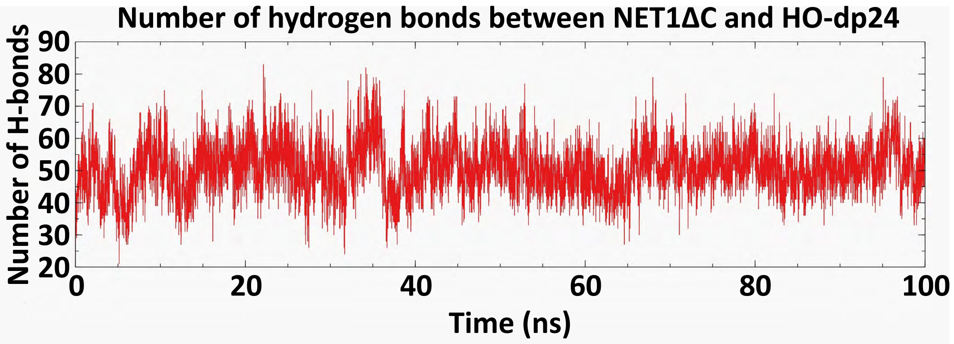

Donor	Acceptor	Occupancy
ARG351	dp10(4)SO3(II)	68.83%
CYS352	dp10(3)SO3(II)	35.36%
TYR325	dp10(3)SO3(II)	24.86%
ARG374	dp10(3)SO3(I)	24.58%
ARG350	dp10(1)SO3(II)	22.23%
ARG351	dp10(6)SO3(I)	21.57%
ARG374	dp10(2)SO3(II)	19.57%
MET356	dp10(9)SO3(II)	18.03%
ARG353	dp10(9)SO3(I)	17.46%
ASN372	dp10(3)SO3(I)	15.72%
ASN355	dp10(9)SO3(II)	15.45%
PHE323	dp10(3)SO3(II)	12.21%
ARG350	dp10(3)SO3(I)	10.86%

Donor	Acceptor	Occupancy
ARG374	dp24(17)SO3(II)	71.84%
ASN372	dp24(14)SO3(II)	70.28%
LYS360	dp24(6)SO3(I)	66.11%
ARG350	dp24(16)SO3(I)	65.27%
ASN355	dp24(9)SO3(II)	64.83%
ARG353	dp24(9)SO3(II)	63.97%
LYS360	dp24(6)SO3(II)	62.10%
ARG400	dp24(18)SO3(I)	60.15%
LYS401	dp24(20)SO3(I)	59.84%
ARG364	dp24(5)SO3(I)	58.54%
ASN372	dp24(13)SO3(II)	49.64%
ARG400	dp24(20)SO3(I)	47.39%
ARG353	dp24(9)SO3(I)	44.97%
ARG380	dp24(9)SO3(II)	43.03%
LYS386	dp24(15)SO3(I)	41.26%
ARG351	dp24(13)SO3(II)	38.49%
ARG350	dp24(14)SO3(II)	37.82%
LYS386	dp24(14)SO3(II)	37.53%
LYS395	dp24(22)SO3(II)	37.37%
ARG350	dp24(17)SO3(II)	36.93%
ASN355	dp24(8)SO3(I)	36.49%
ASN355	dp24(9)SO3(I)	35.94%
LYS401	dp24(22)SO3(I)	35.91%

Supplementary Figure 7

Hydrogen bond analysis (top row) shows a stable number of hydrogen bonds throughout the MD simulation, indicating a stable binding of NET1ΔC to HO-dp10 and HO-dp24. A total of 63 and 191 hydrogen bonds with different occupancy were found throughout the MD simulation for NET1ΔC-dp10 and NET1ΔC-dp24, respectively. The tables show only the hydrogen bonds with occupancy cut-offs above 10% for NET1ΔC-dp10 and 35% for NET1ΔC-dp24, respectively. Any hydrogen bond with high percentage of occupation during the MD simulation time indicates strong interaction between NET1ΔC and the respective oligosaccharide unit. Each row indicates the proton donor and the respective proton acceptor identity. The nomenclature for the GAG molecule follows the naming definition by GLYCAM force field. For example, dp10(4)SO3(II) represents a sulphate group of the 4th residue of dp10 with sulphate groups attached to oxygens named SO3(I) whereas sulphate groups attached to nitrogen are named SO3(II).

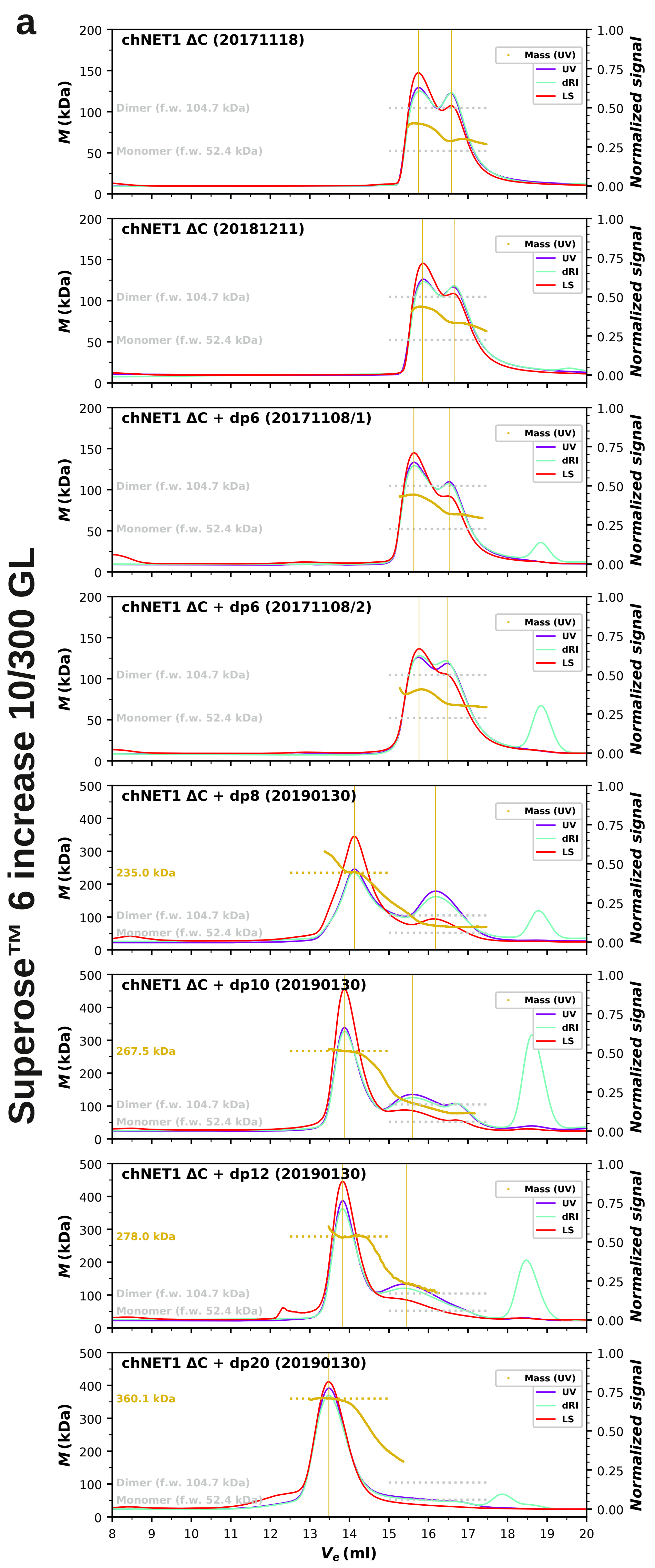

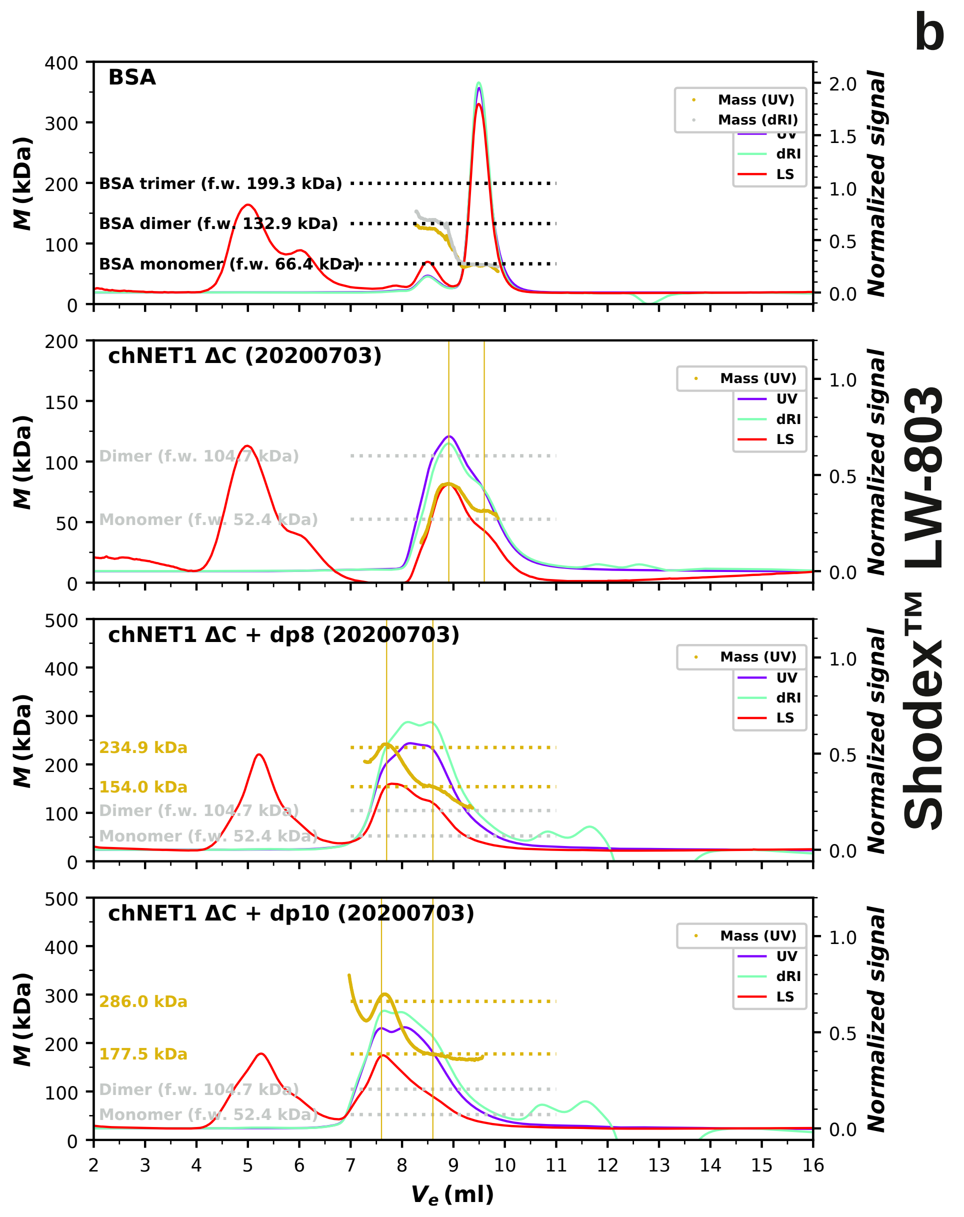

Supplementary Figure 8

SEC-MALS traces of NET1ΔC alone and NET1ΔC associated with different sized heparin oligosaccharides (HO-dp6 to HO-dp20). HO-dp6 does not affect the monomer/dimer equilibrium. A shift to larger mass starts with HO-dp8. There is an increase of mass with increasing HO chain length. Also apparent is the complete disappearance of monomeric and dimeric NET1ΔC with the longest HO chain length. (**a**) Elution from the Cytiva Superose™ 6 increase 10/300 GL SEC column. (**b**) Elution from the ShodexTM LW-803 SEC column. The mass distribution across the elution peak is shown in gold, the $UV_{280\ nm}$ trace in blue, the differential refractive index change in green and the light scattering signal (90° angle) in red. Expected masses for NET1ΔC monomers and dimers are shown in silver. The peak positions used to determine the masses of Supplement Table 2 are marked by vertical lines.

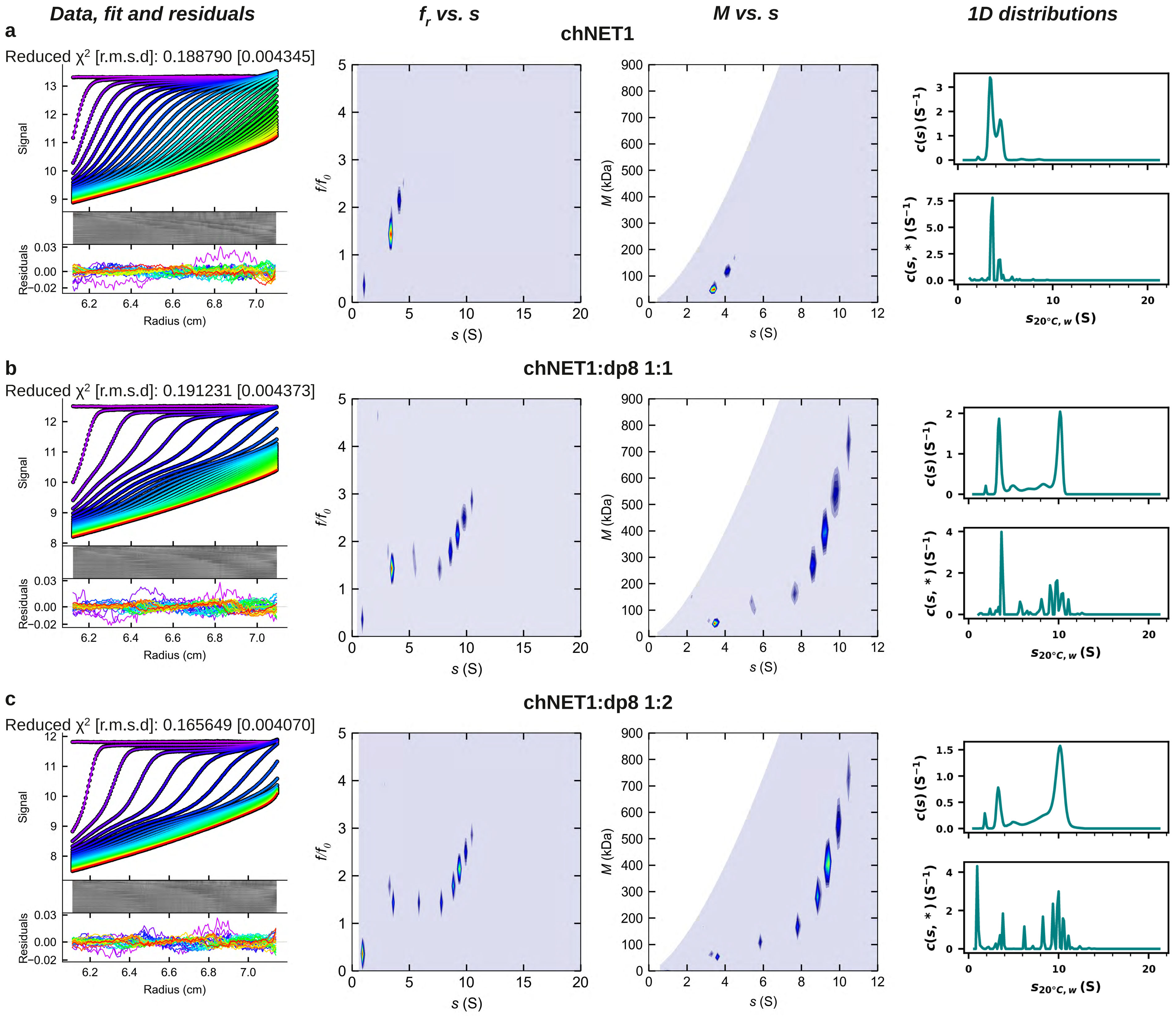

Supplementary Figure 9

(**a**) The results of the sedimentation velocity experiment with NET1ΔC in the absence of HO. The left panel shows the fit to the interference data and the residuals. The second panel depicts the *c(s, f_r)* distribution as calculated in SEDFIT[16]. In the third panel, the 2-dimensional distribution was converted to mass, yielding the *c(s, M)* distribution. The right panel shows the one-dimensional *c(s)* distribution in the upper subplot and the *c(s, *)* distribution in the lower subplot. Two species are discernible that correspond to monomeric and dimeric NET1ΔC. (**b**) and (**c**) The results of the sedimentation velocity experiment with NET1ΔC in the presence of HO-dp8 in a 1:1 and 1:2 molar ratio. The left panel shows the fit to the interference data and the residuals. The second panel depicts the *c(s, f_r)* distribution as calculated in SEDFIT[16]. In the third panel, the 2-dimensional distribution was converted to mass, yielding the *c(s, M)* distribution. The right panel shows the one-dimensional *c(s)* distribution in the upper subplot and the *c(s, *)* distribution in the lower subplot. The plots show a series of distinct populations of increasing molecular mass, spaced approximately 100 - 200 kDa apart. This suggests a stepwise addition of NET1ΔC molecules to the HO moieties.

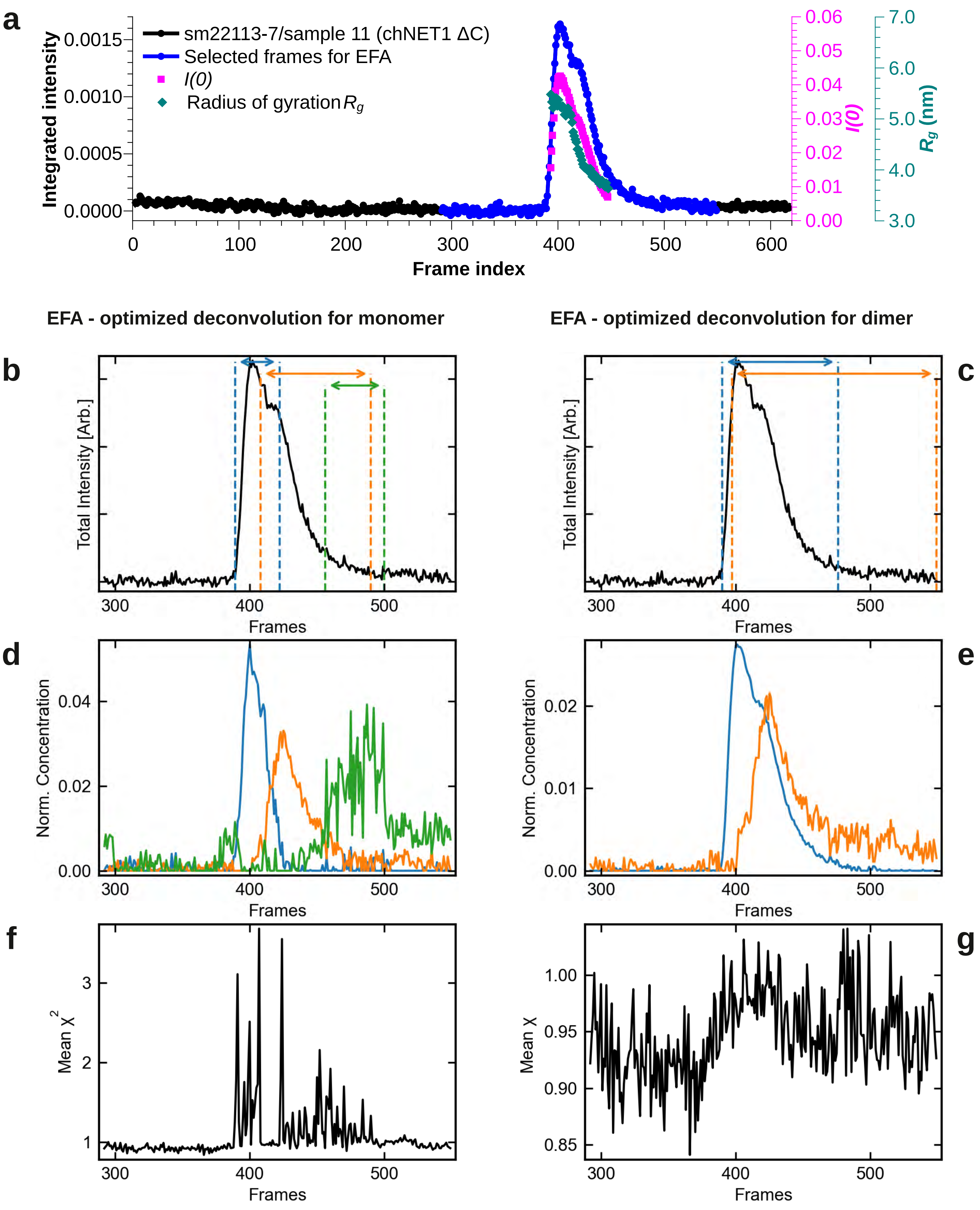

Supplementary Figure 10

Evolving factor analysis (EFA) and deconvolution of the SEC-SAXS dataset sm22113-7/sample11 with BioXTAS RAW[7, 17]. (**a**) Integrated scattering signal of NET1ΔC eluting at 0.16 ml/min from the Shodex KW404-4F size exclusion column (black curve) as recorded by a sequence of frames taken every 3 seconds. The chosen frames for EFA are highlighted in blue. The extrapolated intensity at 0 angle *I(0)* and the calculated radius of gyration R_g for the frames of the elution peaks are shown in magenta and teal, respectively. (**b, c**) Elution peak ranges as determined by EFA for the monomeric (orange) and dimeric (blue) scattering components. Optimization of the scattering profile for the monomeric species required the removal of a third scattering component (green, panel **b**). The scattering profile for the dimer was of better quality without the inclusion of the third component (panel **c**). (**d, e**) Deconvolution of the monomeric (orange) and dimeric (blue) scattering components by single value decomposition (SVD), optimized to obtain the best scattering profile for the monomeric component (panel **d**) or the dimeric component (panel **e**). (**f, g**) Mean error weighted χ^2 plots of for the SVD vector rotation optimizing the scattering profile of the monomeric component (**f**) or the dimeric component (**g**).

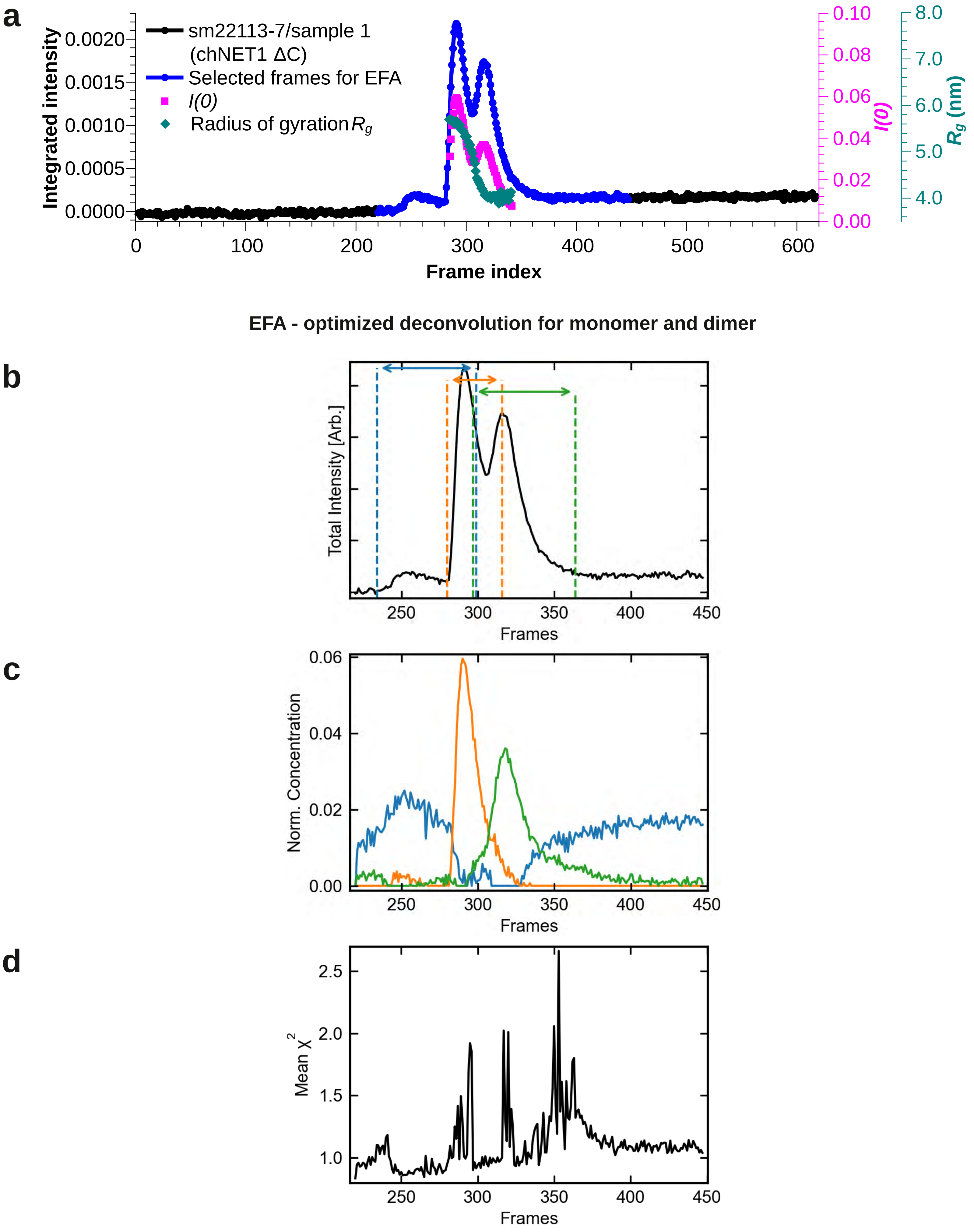

Supplementary Figure 11

Evolving factor analysis (EFA) and deconvolution of the SEC-SAXS dataset sm22113-7/sample1 with BioXTAS RAW. (**a**) Integrated scattering signal of NET1ΔC eluting at 0.16 ml/min from the Shodex KW403-4F size exclusion column (black curve) as recorded by a sequence of frames taken every 3 seconds. The chosen frames for EFA are highlighted in blue. The extrapolated intensity at 0 angle *I(0)* and the calculated radius of gyration R_g for the frames of the elution peaks are shown in magenta and teal, respectively. (**b**) Elution peak ranges as determined by EFA for the monomeric (green) and dimeric (orange) scattering components. (**c**) Deconvolution of the monomeric (green) and dimeric (orange) scattering components by single value decomposition (SVD). The baseline drift could be compensated for by including a third scattering component (blue). (**d**) Mean error weighted χ^2 plot of for the SVD vector rotation.

Deconvoluted NET1ΔC monomer - experiment ID sm22113-7/sample 11

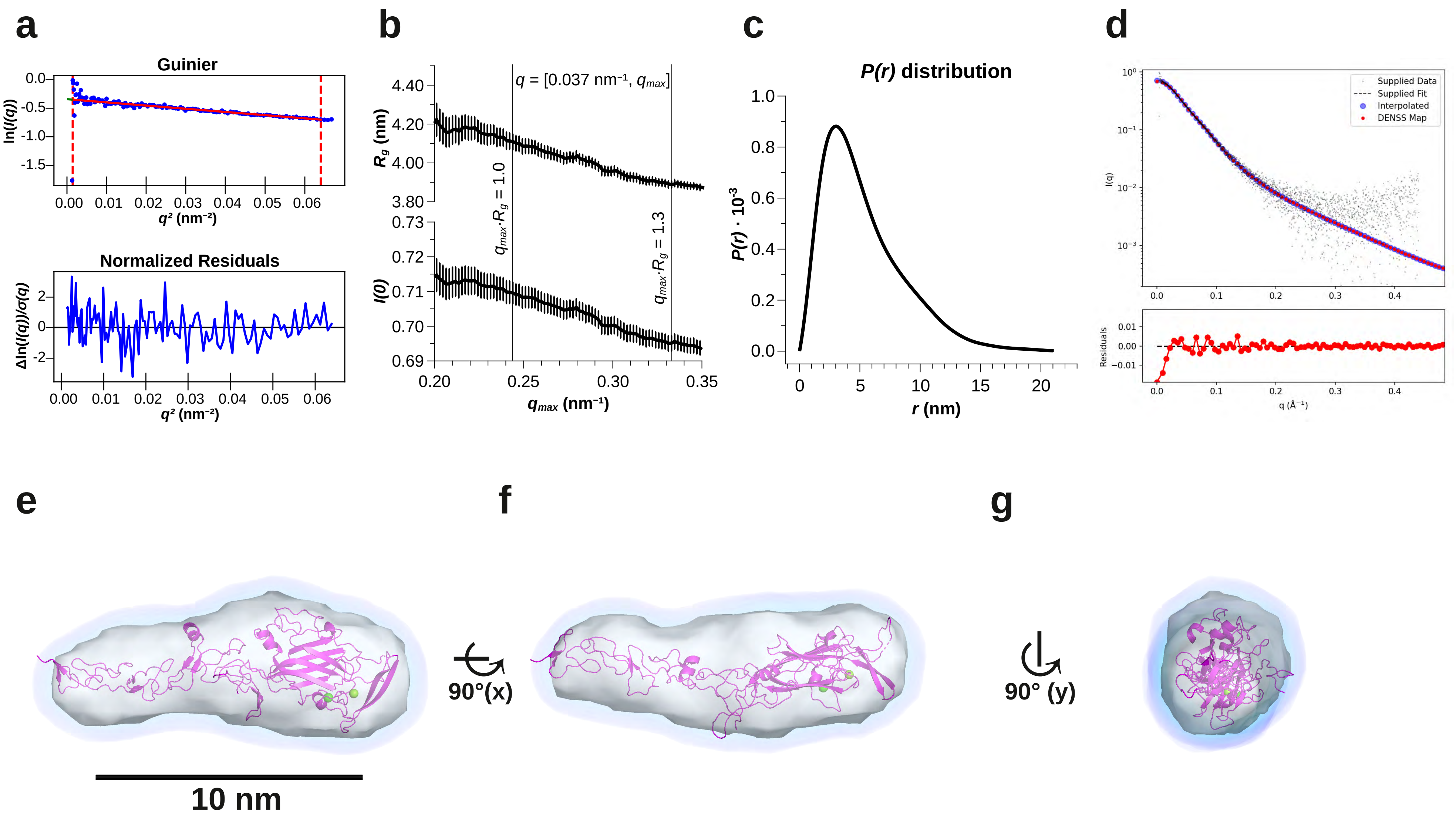

Supplementary Figure 12

Scattering profile of the monomeric component of SEC-SAXS dataset sm22113-7/sample11 and electron density (ED) reconstruction of the monomeric NET1ΔC by DENSS[3]. (**a**) Guinier fit (red line) to the data (blue dots) at low scattering angle q, yielding the scattering intensity at 0 angle $I(0)$ by extrapolation (green line), and the radius of gyration R_g of the scattering particle (see Supplementary Table 5a)[18]. The residuals of the fit are shown in the bottom half of the panel. (**b**) Variation of $I(0)$ and R_g obtained from iterative Guinier fits to data between a fixed q_{min} and an increasing q_{max} (abscissa). The scattering angles where $q_{max} \cdot R_g = 1.0$ and $q_{max} \cdot R_g = 1.3$ are marked by vertical lines. The Guinier approximation of a rod shaped particle would be linear up to the first vertical line and that of the globular particle up to the second vertical line. (**c**) Pair distance distribution $P(r)$ obtained from the scattering profile by indirect Fourier transform (IFT). (**d**) The top half of the panel shows the experimental scattering profile (black dots), reverse IFT of the $P(r)$ distribution (blue dots) and the scattering profile calculated from the ED map (red dots). The residuals from the scattering profile of the ED map to the experimental scattering profile are shown in the bottom half of the panel. (**e, f, g**): 3D-reconstruction of the ED map from the scattering data. The blue-shaded region encompasses the support volume reported by DENSS. The surface representation is rendered at an ED level that encloses the particle volume reported by DAMMIN (see Supplementary Table 5a). The X-ray crystal structure of monomeric *Gallus gallus* NET1ΔC (PDB ID: 4PLM) is fit into the ED map[19]. Models shown in panels **f** and **g** are rotated 90° around the long axis (x) or the short axis (y), respectively, compared to the model in panel **e**. The scale bar indicates a length of 10 nm.

Deconvoluted NET1ΔC dimer - experiment ID sm22113-7/sample 11

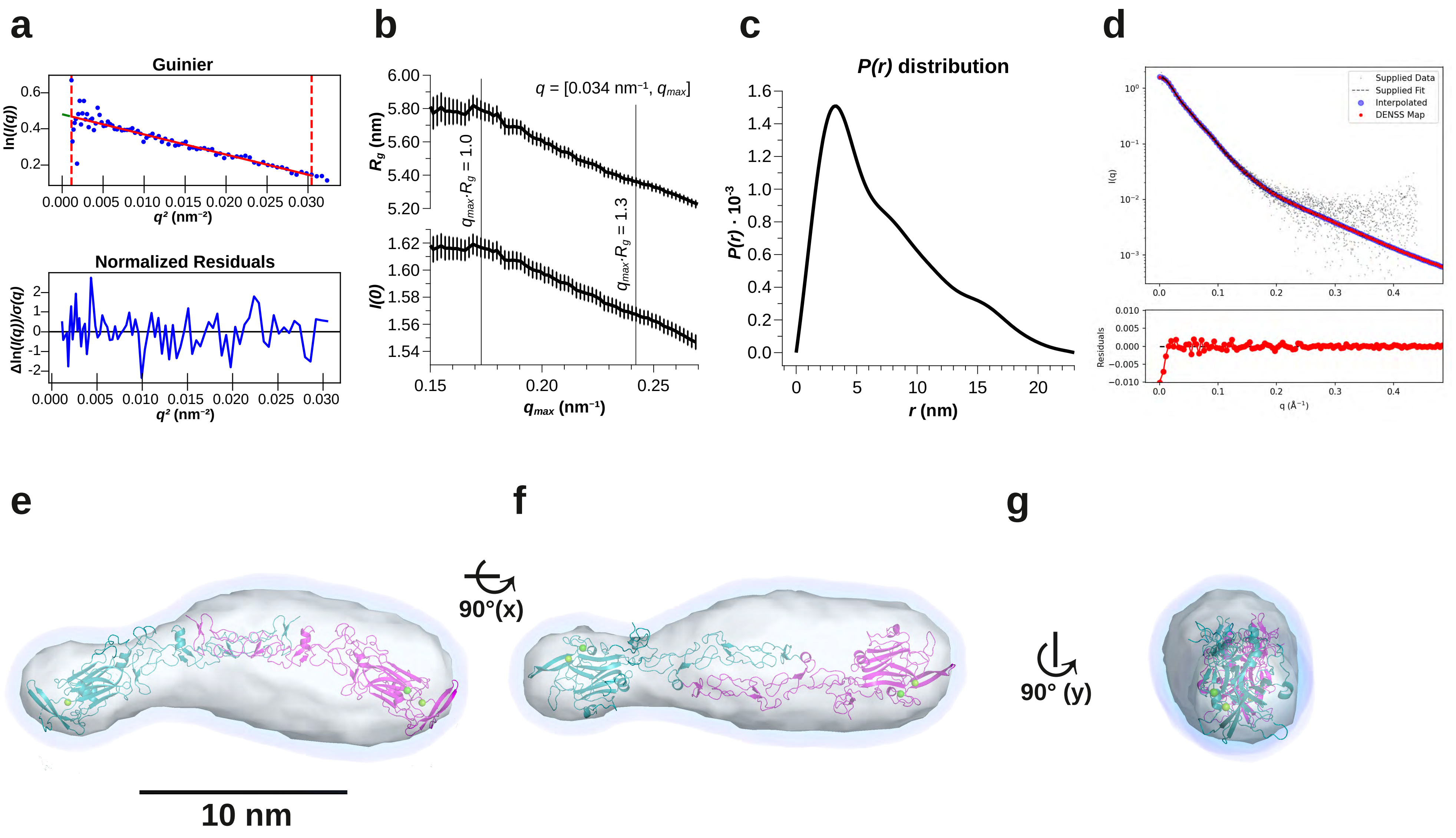

Supplementary Figure 13

Scattering profile of the dimeric component of SEC-SAXS dataset sm22113-7/sample11 and electron density (ED) reconstruction of the dimeric NET1ΔC by DENSS. (**a**) Guinier fit (red line) to the data (blue dots) at low scattering angle q, yielding the scattering intensity at 0 angle *I(0)* by extrapolation (green line), and the radius of gyration R_g of the scattering particle (see Supplementary Table 5b). The residuals of the fit are shown in the bottom half of the panel. (**b**) Variation of *I(0)* and R_g obtained from iterative Guinier fits to data between a fixed q_{min} and an increasing q_{max} (abscissa). The scattering angles where $q_{max} \cdot R_g = 1.0$ and $q_{max} \cdot R_g = 1.3$ are marked by vertical lines. The Guinier approximation of a rod shaped particle would be linear up to the first vertical line and that of the globular particle up to the second vertical line. (**c**) Pair distance distribution *P(r)* obtained from the scattering profile by indirect Fourier transform (IFT). (**d**) The top half of the panel shows the experimental scattering profile (black dots), reverse IFT of the *P(r)* distribution (blue dots) and the scattering profile calculated from the ED map (red dots). The residuals from the scattering profile of the ED map to the experimental scattering profile are shown in the bottom half of the panel. (**e, f, g**): 3D-reconstruction of the ED map from the scattering data. The blue-shaded region encompasses the support volume reported by DENSS. The surface representation is rendered at an ED level that encloses the particle volume reported by DAMMIN (see Supplementary Table 5b). The X-ray crystal structure of dimeric *Gallus gallus* NET1ΔC (PDB ID: 4PLM) is fit into the ED map[19]. Models shown in panels **f** and **g** are rotated 90° around the long axis (x) or the short axis (y), respectively, compared to the model in panel **e**. The scale bar indicates a length of 10 nm.

Deconvoluted NET1ΔC monomer - experiment ID sm22113-7/sample 1

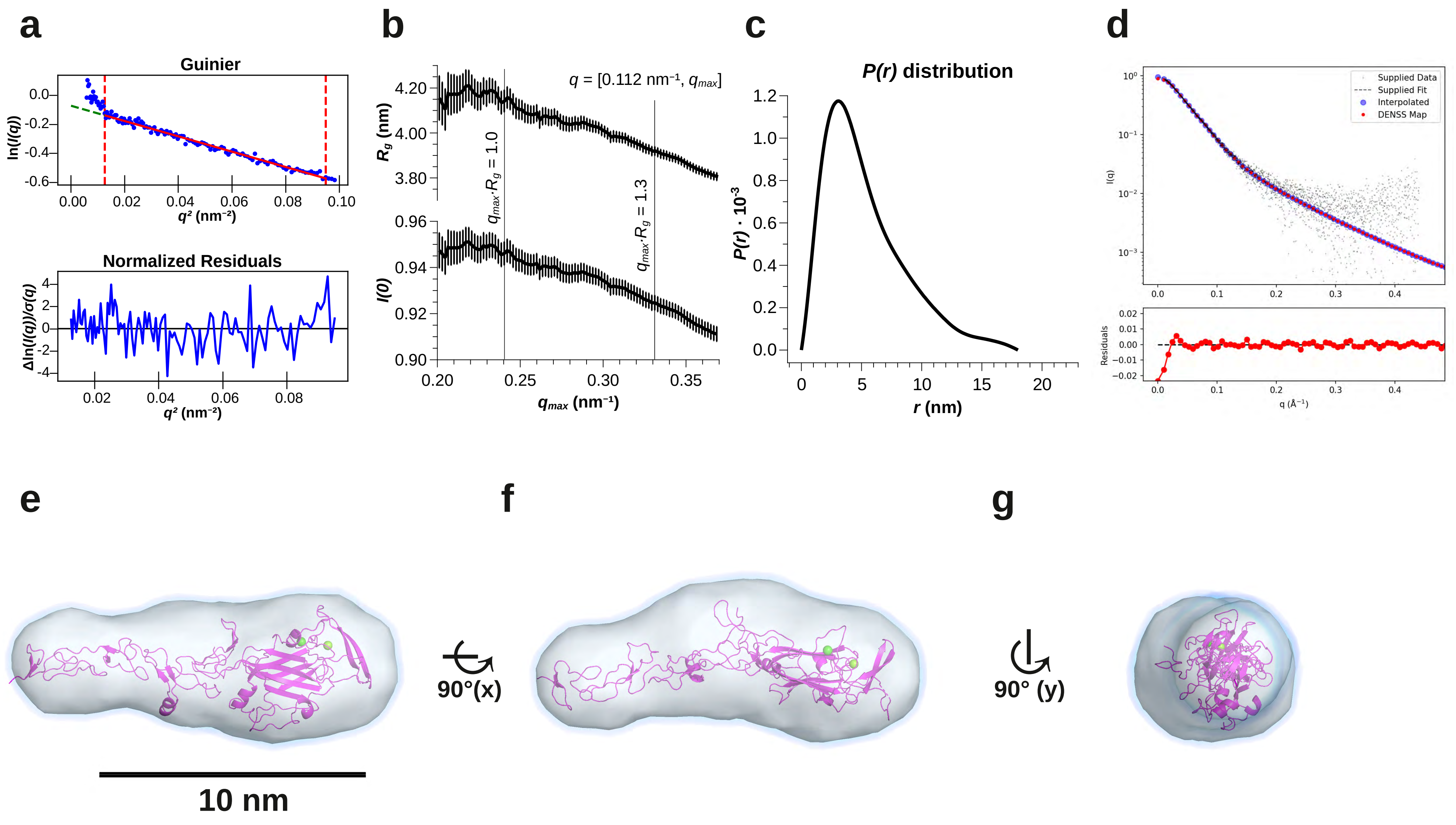

Supplementary Figure 14

Scattering profile of the monomeric component of SEC-SAXS dataset sm22113-7/sample1 and electron density (ED) reconstruction of the monomeric NET1ΔC by DENSS. (**a**) Guinier fit (red line) to the data (blue dots) at low scattering angle *q*, yielding the scattering intensity at 0 angle *I(0)* by extrapolation (green line), and the radius of gyration R_g of the scattering particle (see Supplementary Table 5a). The residuals of the fit are shown in the bottom half of the panel. (**b**) Variation of *I(0)* and R_g obtained from iterative Guinier fits to data between a fixed q_{min} and an increasing q_{max} (abscissa). The scattering angles where $q_{max} \cdot R_g = 1.0$ and $q_{max} \cdot R_g = 1.3$ are marked by vertical lines. The Guinier approximation of a rod shaped particle would be linear up to the first vertical line and that of the globular particle up to the second vertical line. (**c**) Pair distance distribution *P(r)* obtained from the scattering profile by indirect Fourier transform (IFT). (**d**) The top half of the panel shows the experimental scattering profile (black dots), reverse IFT of the *P(r)* distribution (blue dots) and the scattering profile calculated from the ED map (red dots). The residuals from the scattering profile of the ED map to the experimental scattering profile are shown in the bottom half of the panel. (**e, f, g**): 3D-reconstruction of the ED map from the scattering data. The blue-shaded region encompasses the support volume reported by DENSS. The surface representation is rendered at an ED level that encloses the particle volume reported by DAMMIN (see Supplementary Table 5a). The X-ray crystal structure of monomeric *Gallus gallus* NET1ΔC (PDB ID: 4PLM) is fit into the ED map. Models shown in panels **f** and **g** are rotated 90° around the long axis (x) or the short axis (y), respectively, compared to the model in panel **e**. The scale bar indicates a length of 10 nm.

Deconvoluted NET1ΔC dimer - experiment ID sm22113-7/sample 1

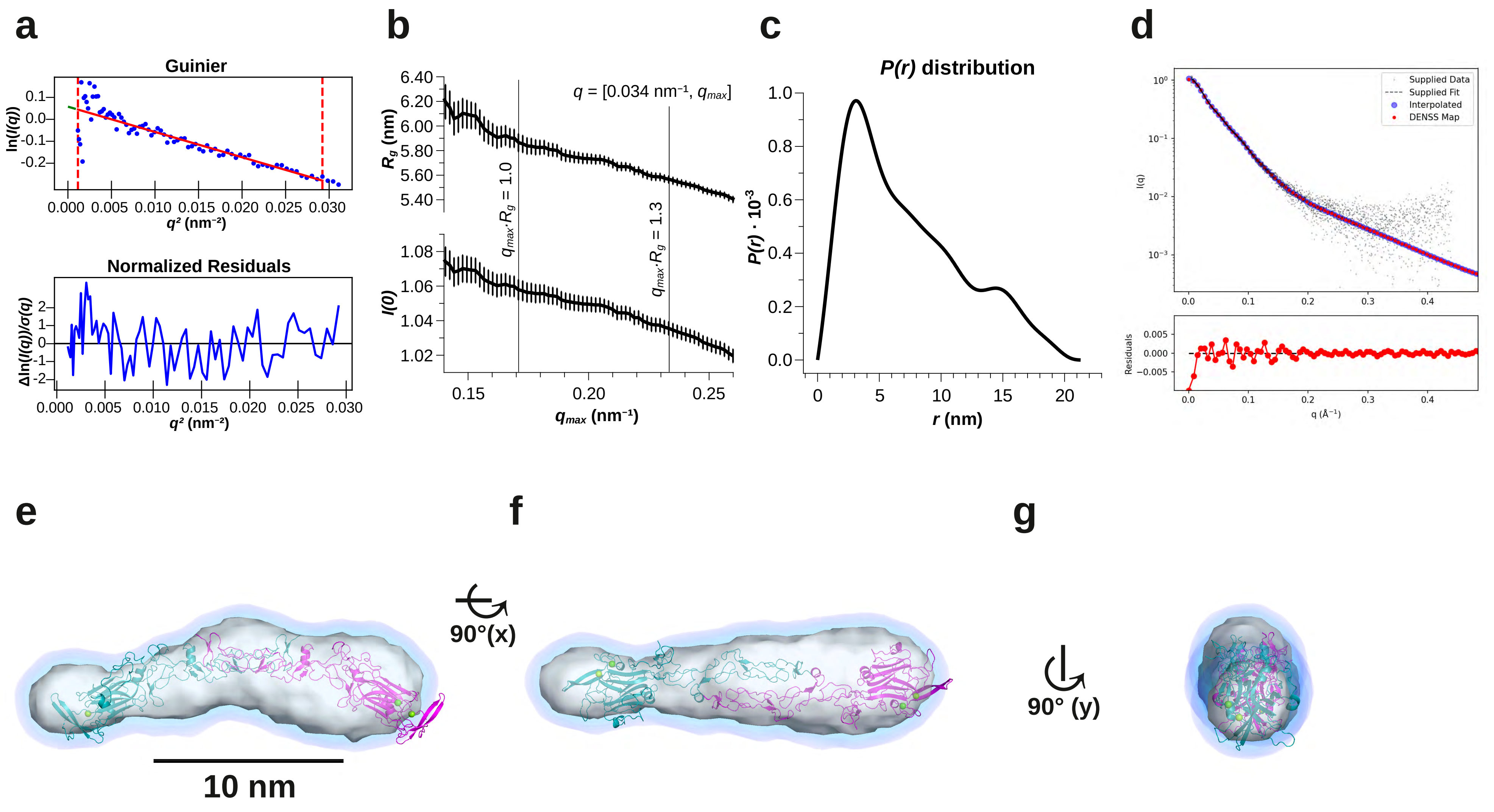

Supplementary Figure 15

Scattering profile of the dimeric component of SEC-SAXS dataset sm22113-7/sample1 and electron density (ED) reconstruction of the dimeric NET1ΔC by DENSS. (**a**) Guinier fit (red line) to the data (blue dots) at low scattering angle q, yielding the scattering intensity at 0 angle *I(0)* by extrapolation (green line), and the radius of gyration R_g of the scattering particle (see Supplementary Table 5b). The residuals of the fit are shown in the bottom half of the panel. (**b**) Variation of *I(0)* and R_g obtained from iterative Guinier fits to data between a fixed q_{min} and an increasing q_{max} (abscissa). The scattering angles where $q_{max} \cdot R_g = 1.0$ and $q_{max} \cdot R_g = 1.3$ are marked by vertical lines. The Guinier approximation of a rod shaped particle would be linear up to the first vertical line and that of the globular particle up to the second vertical line. (**c**) Pair distance distribution *P(r)* obtained from the scattering profile by indirect Fourier transform (IFT). (**d**) The top half of the panel shows the experimental scattering profile (black dots), reverse IFT of the *P(r)* distribution (blue dots) and the scattering profile calculated from the ED map (red dots). The residuals from the scattering profile of the ED map to the experimental scattering profile are shown in the bottom half of the panel. (**e, f, g**): 3D-reconstruction of the ED map from the scattering data. The blue-shaded region encompasses the support volume reported by DENSS. The surface representation is rendered at an ED level that encloses the particle volume reported by DAMMIN (see Supplementary Table 5b). The X-ray crystal structure of dimeric *Gallus gallus* NET1ΔC (PDB ID: 4PLM) is fit into the ED map. Models shown in panels **f** and **g** are rotated 90° around the long axis (x) or the short axis (y), respectively, compared to the model in panel **e**. The scale bar indicates a length of 10 nm.

NET1ΔC with heparin oligosaccharide dp8 - experiment ID sm16028-7/379532

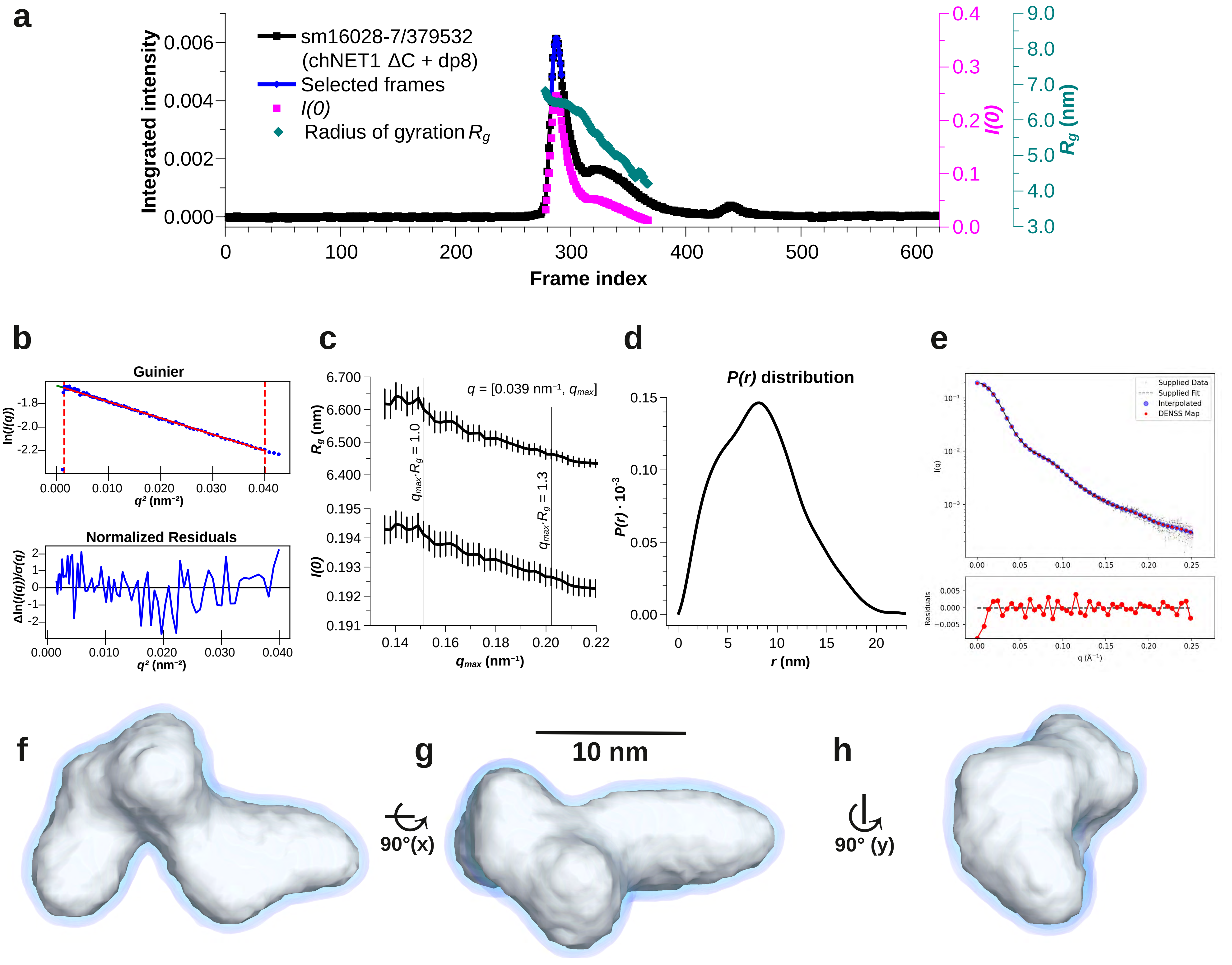

Supplementary Figure 16

Small angle X-ray data from data collection sm16028-7/379532 at beamline B21 (Diamond Light Source)[20] and the corresponding 3D reconstructions of NET1ΔC in presence of HO-dp8. (**a**) Integrated scattering signal of NET1ΔC eluting at 0.16 ml/min from the Shodex KW403-4F size exclusion column (black curve) as recorded by a sequence of frames taken every 3 seconds. The chosen frames for 3D reconstruction are highlighted in blue. The extrapolated intensity at 0 angle *I(0)* and the calculated radius of gyration R_g for the frames of the elution peaks are shown in magenta and teal, respectively. (**b**) Guinier fit (red line) to the data (blue dots) at low scattering angle *q*, yielding the scattering intensity at 0 angle *I(0)* by extrapolation (green line), and the radius of gyration R_g of the scattering particle (see Supplementary Table 5c). The residuals of the fit are shown in the bottom half of the panel. (**c**) Variation of *I(0)* and R_g obtained from iterative Guinier fits to data between a fixed q_{min} and an increasing q_{max} (abscissa). The scattering angles where $q_{max} \cdot R_g = 1.0$ and $q_{max} \cdot R_g = 1.3$ are marked by vertical lines. The Guinier approximation of a rod shaped particle would be linear up to the first vertical line and that of the globular particle up to the second vertical line. (**d**) Pair distance distribution *P(r)* obtained from the scattering profile by indirect Fourier transform (IFT). (**e**) The top half of the panel shows the experimental scattering profile (black dots), reverse IFT of the *P(r)* distribution (blue dots) and the scattering profile calculated from the ED map (red dots). The residuals from the scattering profile of the ED map to the experimental scattering profile are shown in the bottom half of the panel. (**f, g, h**): 3D-reconstruction of the ED map from the scattering data. The blue-shaded region encompasses the support volume reported by DENSS. The surface representation is rendered at an ED level that encloses the particle volume reported by DAMMIN (see Supplementary Table 5c). Models shown in panels **g** and **h** are rotated 90° around the horizontal axis (x) or the vertical axis (y), respectively, compared to the model in panel **f**. The scale bar indicates a length of 10 nm.

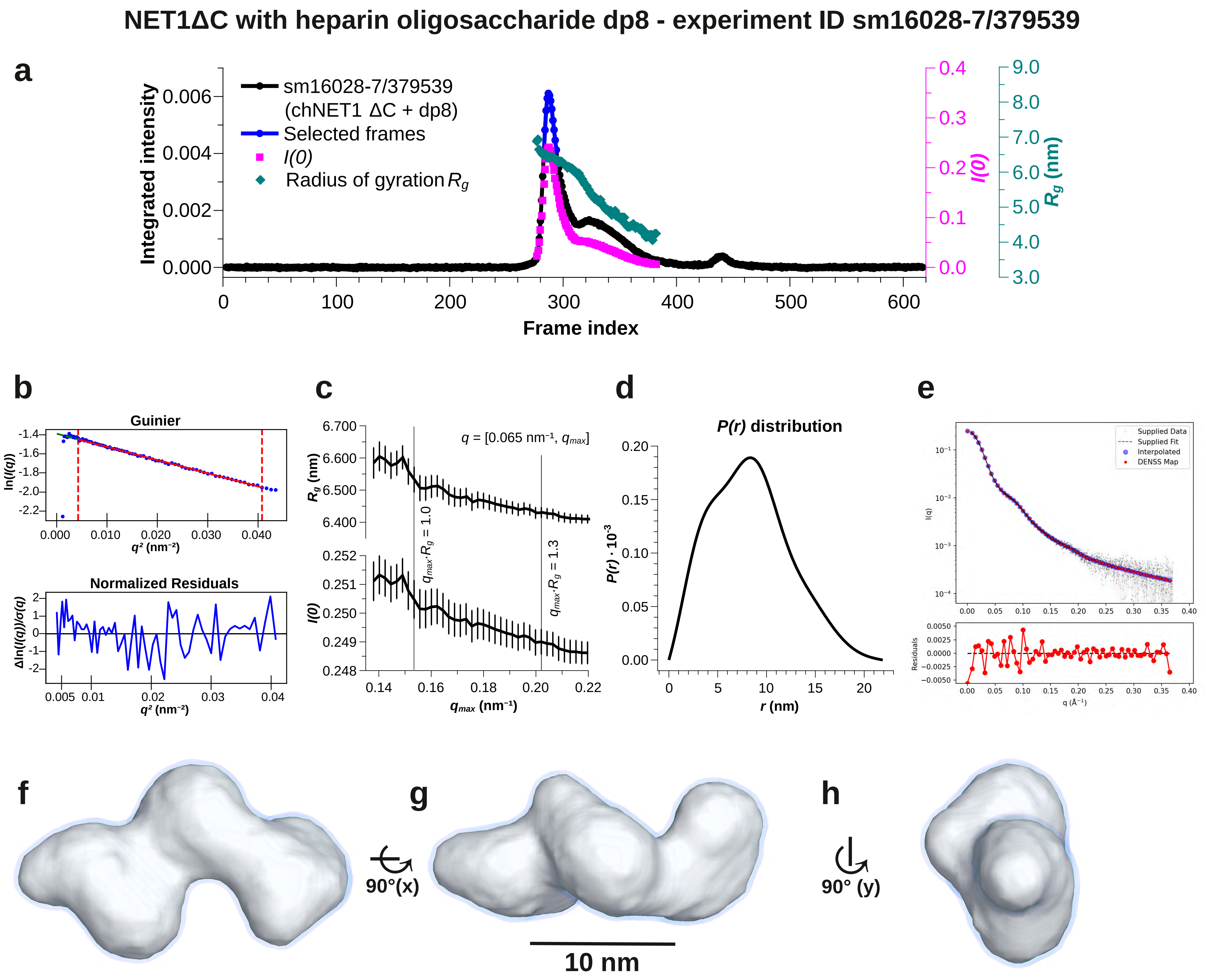

Supplementary Figure 17

Small angle X-ray data from data collection sm16028-7/379539 at beamline B21 (Diamond Light Source)[20] and the corresponding 3D reconstructions of NET1ΔC in presence of HO-dp8. (**a**) Integrated scattering signal of NET1ΔC eluting at 0.16 ml/min from the Shodex KW403-4F size exclusion column (black curve) as recorded by a sequence of frames taken every 3 seconds. The chosen frames for 3D reconstruction are highlighted in blue. The extrapolated intensity at 0 angle *I(0)* and the calculated radius of gyration R_g for the frames of the elution peaks are shown in magenta and teal, respectively. (**b**) Guinier fit (red line) to the data (blue dots) at low scattering angle *q*, yielding the scattering intensity at 0 angle *I(0)* by extrapolation (green line), and the radius of gyration R_g of the scattering particle (see Supplementary Table 5c). The residuals of the fit are shown in the bottom half of the panel. (**c**) Variation of *I(0)* and R_g obtained from iterative Guinier fits to data between a fixed q_{min} and an increasing q_{max} (abscissa). The scattering angles where $q_{max} \cdot R_g = 1.0$ and $q_{max} \cdot R_g = 1.3$ are marked by vertical lines. The Guinier approximation of a rod shaped particle would be linear up to the first vertical line and that of the globular particle up to the second vertical line. (**d**) Pair distance distribution *P(r)* obtained from the scattering profile by indirect Fourier transform (IFT). (**e**) The top half of the panel shows the experimental scattering profile (black dots), reverse IFT of the *P(r)* distribution (blue dots) and the scattering profile calculated from the ED map (red dots). The residuals from the scattering profile of the ED map to the experimental scattering profile are shown in the bottom half of the panel. (**f, g, h**): 3D-reconstruction of the ED map from the scattering data. The blue-shaded region encompasses the support volume reported by DENSS. The surface representation is rendered at an ED level that encloses the particle volume reported by DAMMIN (see Supplementary Table 5c). Models shown in panels **g** and **h** are rotated 90° around the horizontal axis (x) or the vertical axis (y), respectively, compared to the model in panel **f**. The scale bar indicates a length of 10 nm.

NET1ΔC with heparin oligosaccharide dp10 - experiment ID sm16028-7/379533

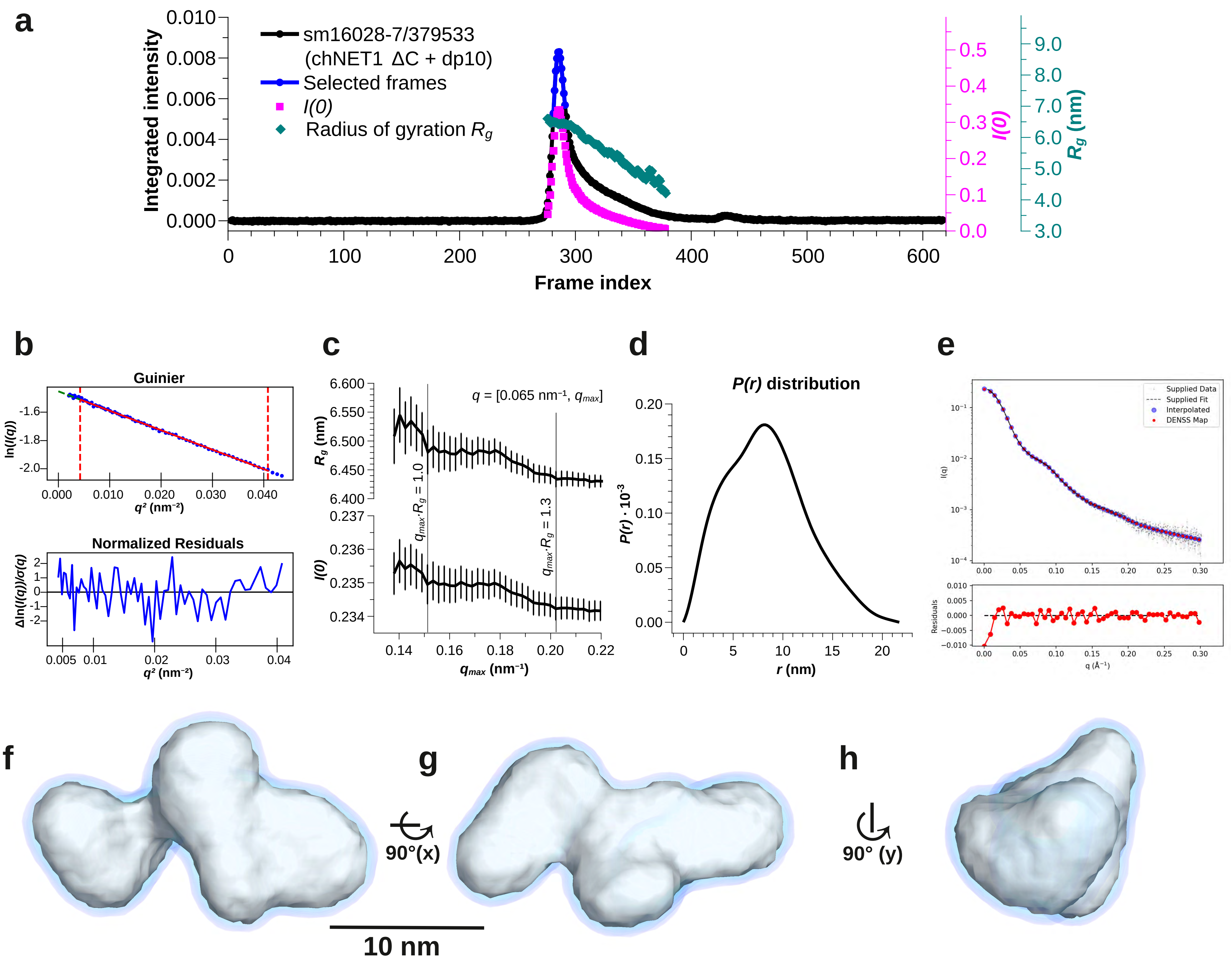

Supplementary Figure 18

Small angle X-ray data from data collection sm16028-7/379533 at beamline B21 (Diamond Light Source)[20] and the corresponding 3D reconstructions of NET1ΔC in presence of HO-dp10. (**a**) Integrated scattering signal of NET1ΔC eluting at 0.16 ml/min from the Shodex KW403-4F size exclusion column (black curve) as recorded by a sequence of frames taken every 3 seconds. The chosen frames for 3D reconstruction are highlighted in blue. The extrapolated intensity at 0 angle *I(0)* and the calculated radius of gyration R_g for the frames of the elution peaks are shown in magenta and teal, respectively. (**b**) Guinier fit (red line) to the data (blue dots) at low scattering angle *q*, yielding the scattering intensity at 0 angle *I(0)* by extrapolation (green line), and the radius of gyration R_g of the scattering particle (see Supplementary Table 5d). The residuals of the fit are shown in the bottom half of the panel. (**c**) Variation of *I(0)* and R_g obtained from iterative Guinier fits to data between a fixed q_{min} and an increasing q_{max} (abscissa). The scattering angles where $q_{max} \cdot R_g = 1.0$ and $q_{max} \cdot R_g = 1.3$ are marked by vertical lines. The Guinier approximation of a rod shaped particle would be linear up to the first vertical line and that of the globular particle up to the second vertical line. (**d**) Pair distance distribution *P(r)* obtained from the scattering profile by indirect Fourier transform (IFT). (**e**) The top half of the panel shows the experimental scattering profile (black dots), reverse IFT of the *P(r)* distribution (blue dots) and the scattering profile calculated from the ED map (red dots). The residuals from the scattering profile of the ED map to the experimental scattering profile are shown in the bottom half of the panel. (**f, g, h**): 3D-reconstruction of the ED map from the scattering data. The blue-shaded region encompasses the support volume reported by DENSS. The surface representation is rendered at an ED level that encloses the particle volume reported by DAMMIN (see Supplementary Table 5d). Models shown in panels **g** and **h** are rotated 90° around the horizontal axis (x) or the vertical axis (y), respectively, compared to the model in panel **f**. The scale bar indicates a length of 10 nm.

NET1ΔC with heparin oligosaccharide dp10 - experiment ID sm16028-7/379540

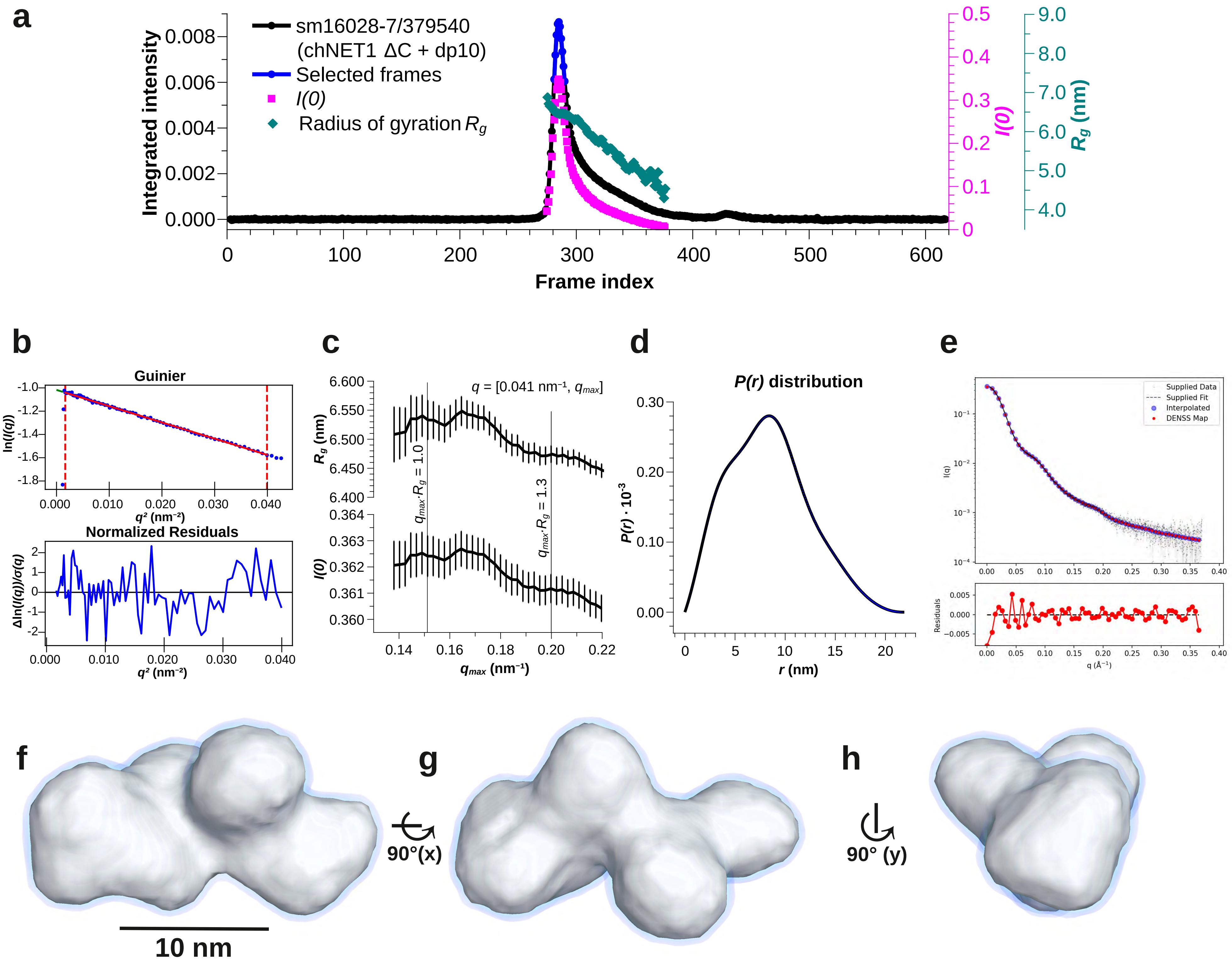

Supplementary Figure 19

Small angle X-ray data from data collection sm16028-7/379540 at beamline B21 (Diamond Light Source)[20] and the corresponding 3D reconstructions of NET1ΔC in presence of HO-dp10. (**a**) Integrated scattering signal of NET1ΔC eluting at 0.16 ml/min from the Shodex KW403-4F size exclusion column (black curve) as recorded by a sequence of frames taken every 3 seconds. The chosen frames for 3D reconstruction are highlighted in blue. The extrapolated intensity at 0 angle *I(0)* and the calculated radius of gyration R_g for the frames of the elution peaks are shown in magenta and teal, respectively. (**b**) Guinier fit (red line) to the data (blue dots) at low scattering angle q, yielding the scattering intensity at 0 angle *I(0)* by extrapolation (green line), and the radius of gyration R_g of the scattering particle (see Supplementary Table 5d). The residuals of the fit are shown in the bottom half of the panel. (**c**) Variation of *I(0)* and R_g obtained from iterative Guinier fits to data between a fixed q_{min} and an increasing q_{max} (abscissa). The scattering angles where $q_{max} \cdot R_g = 1.0$ and $q_{max} \cdot R_g = 1.3$ are marked by vertical lines. The Guinier approximation of a rod shaped particle would be linear up to the first vertical line and that of the globular particle up to the second vertical line. (**d**) Pair distance distribution *P(r)* obtained from the scattering profile by indirect Fourier transform (IFT). (**e**) The top half of the panel shows the experimental scattering profile (black dots), reverse IFT of the *P(r)* distribution (blue dots) and the scattering profile calculated from the ED map (red dots). The residuals from the scattering profile of the ED map to the experimental scattering profile are shown in the bottom half of the panel. (**f, g, h**): 3D-reconstruction of the ED map from the scattering data. The blue-shaded region encompasses the support volume reported by DENSS. The surface representation is rendered at an ED level that encloses the particle volume reported by DAMMIN (see Supplementary Table 5d). Models shown in panels **g** and **h** are rotated 90° around the horizontal axis (x) or the vertical axis (y), respectively, compared to the model in panel **f**. The scale bar indicates a length of 10 nm.

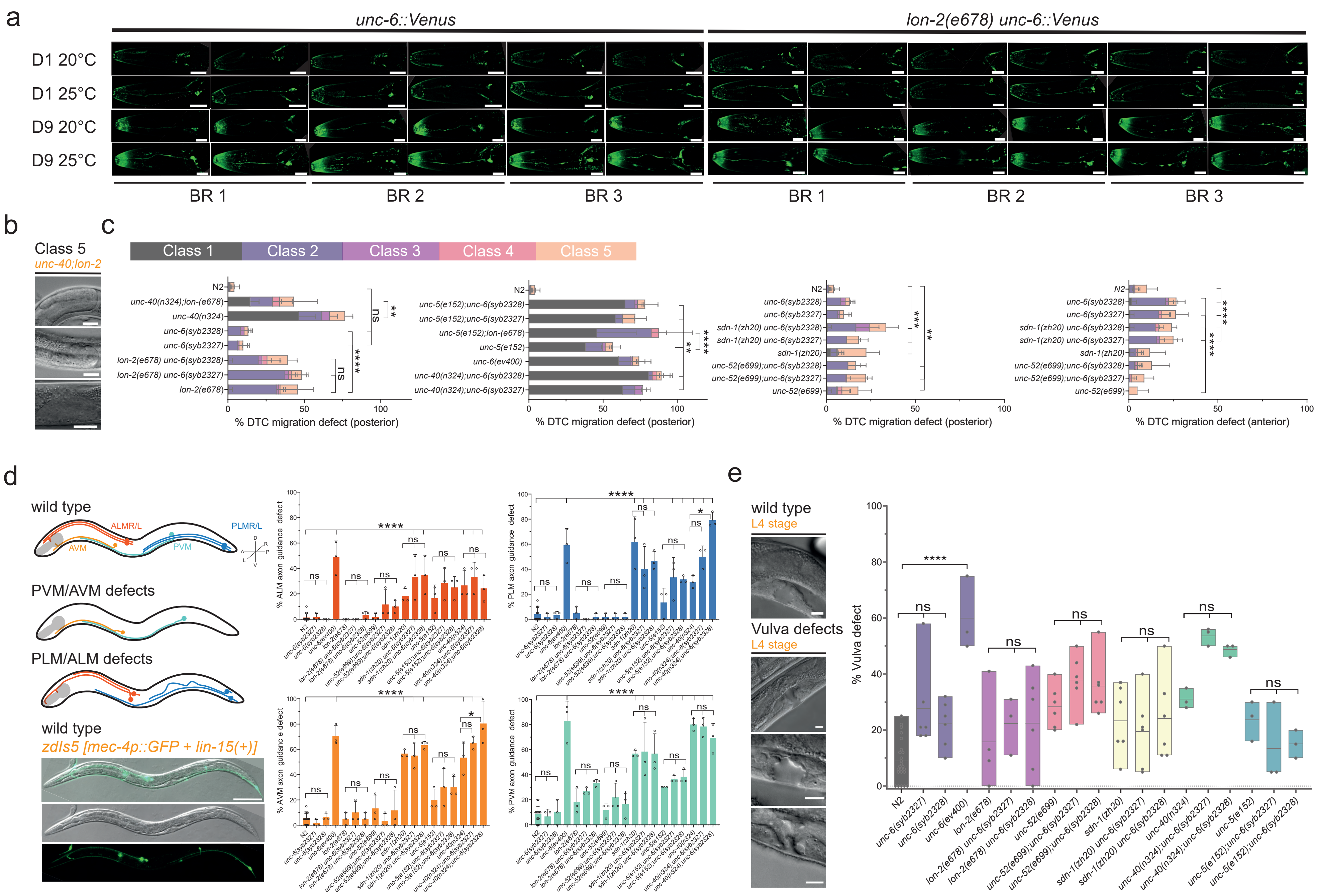

Supplementary Figure 20

(**a**) Panel of confocal fluorescent images of NET1/UNC-6 puncta in the head region of day 1 and day 9 adult worms grown at 20 °C or 25 °C. Two representative images for biological replicates (BR1-3) for each condition were chosen. Scale bar: 20 µm. (**b**) Representative Differential Interference Contrast (DIC) micrograph of frequently observed class 5 DTC defect in *unc-40*/DCC*;lon-2*/Glypican double mutants. L4 staged worms, lateral view, scale bar: 20 µm. (**c**) Quantification of DTC migration defects of posterior and anterior gonad arms of single and double mutants of two independently generated *unc-6 (loop cd)*/NET1 mutants (*(unc-6(syb2327)* & *unc-6(syb2328)*), with *lon-2*/glypican, *unc-52*/perlecan, *sdn-1*/syndecan, *unc-5*/UNC-5 and *unc-40*/DCC mutants. Error bars represent the mean (SD). Results for ordinary 1-way ANOVA Tukey multiple comparisons test are indicated: ****, $P < 0.0001$; ***, $P < 0.0008$; **, $P < 0.0072$; ns, not significant. Samples size for genotypes were $N = 60$ for crosses with *unc-40(n324)* and *unc-5(e152)*; $N = 120$ for crosses with HSPGs; N = >300 for wildtype N2. (**d**) Left: Schematic overview of sensory PVM/AVM, PLM/ALM neurons of wild type animals (top), summary of observed PVM/AVM defects (middle) and PLM/ALM defects (bottom). Fluorescence and DIC image of wild type worms shown in very bottom, scale bar 200 µm. Right: Quantification of PVM/AVM, PLM/ALM defects of single and double mutants. Error bars represent the mean (SD). Results for ordinary 2-way ANOVA Tukey multiple comparisons test are indicated: ****, $P < 0.0001$; *, $P < 0.0193$; ns, not significant. Samples size for all genotypes was $N = 60$. (**e**) Left: Representative Differential Interference Contrast (DIC) micrograph of L4 staged wild type hermaphrodite vulvas (top) and frequently observed vulva defects (bottom), lateral view, scale bar: 10 µm. Right: Quantification of vulva defects of single and double. Error bars represent the mean (SD). Results for ordinary one-way ANOVA Tukey multiple comparisons test are indicated: ****, $P < 0.0001$; ns, not significant. N = 60 - 110. Floating bars are representing the minima, maxima and the mean as center of at least three independent scorings.

Supplementary References

1. Tucker H*, et al.* Sedimentation Interpretation program. *University of New Hampshire*, https://bitcwiki.sr.unh.edu/index.php?title=Main_Page (2013).

2. Pavlov G, Finet S, Tatarenko K, Korneeva E, Ebel C. Conformation of heparin studied with macromolecular hydrodynamic methods and X-ray scattering. *Eur Biophys J* **32**, 437-449 (2003).

3. Grant TD. Ab initio electron density determination directly from solution scattering data. *Nat Methods* **15**, 191-193 (2018).

4. Svergun DI. Restoring low resolution structure of biological macromolecules from solution scattering using simulated annealing. *Biophys J* **76**, 2879-2886 (1999).

5. Garcia de la Torre J, Llorca O, Carrascosa JL, Valpuesta JM. HYDROMIC: prediction of hydrodynamic properties of rigid macromolecular structures obtained from electron microscopy images. *Eur Biophys J* **30**, 457-462 (2001).

6. Ortega A, Amoros D, Garcia de la Torre J. Prediction of hydrodynamic and other solution properties of rigid proteins from atomic- and residue-level models. *Biophys J* **101**, 892-898 (2011).

7. Hopkins JB, Gillilan RE, Skou S. BioXTAS RAW: improvements to a free open-source program for small-angle X-ray scattering data reduction and analysis. *J Appl Crystallogr* **50**, 1545-1553 (2017).

8. Franke D*, et al.* ATSAS 2.8: a comprehensive data analysis suite for small-angle scattering from macromolecular solutions. *J Appl Crystallogr* **50**, 1212-1225 (2017).

9. Piiadov V, Ares de Araujo E, Oliveira Neto M, Craievich AF, Polikarpov I. SAXSMoW 2.0: Online calculator of the molecular weight of proteins in dilute solution from experimental SAXS data measured on a relative scale. *Protein Sci* **28**, 454-463 (2019).

10. Hajizadeh NR, Franke D, Jeffries CM, Svergun DI. Consensus Bayesian assessment of protein molecular mass from solution X-ray scattering data. *Sci Rep* **8**, 7204 (2018).

11. Petoukhov MV*, et al.* New developments in the ATSAS program package for small-angle scattering data analysis. *J Appl Crystallogr* **45**, 342-350 (2012).

12. Schneider TD, Stephens RM. Sequence logos: a new way to display consensus sequences. *Nucleic Acids Res* **18**, 6097-6100 (1990).

13. Finci LI*, et al.* The crystal structure of netrin-1 in complex with DCC reveals the bifunctionality of netrin-1 as a guidance cue. *Neuron* **83**, 839-849 (2014).

14. Bunkoczi G, Echols N, McCoy AJ, Oeffner RD, Adams PD, Read RJ. Phaser.MRage: automated molecular replacement. *Acta Crystallogr D Biol Crystallogr* **69**, 2276-2286 (2013).

15. McCoy AJ, Grosse-Kunstleve RW, Adams PD, Winn MD, Storoni LC, Read RJ. Phaser crystallographic software. *J Appl Crystallogr* **40**, 658-674 (2007).

16. Schuck P*, et al.* SEDFIT-MSTAR: molecular weight and molecular weight distribution analysis of polymers by sedimentation equilibrium in the ultracentrifuge. *Analyst* **139**, 79-92 (2014).

17. Meisburger SP, Taylor AB, Khan CA, Zhang S, Fitzpatrick PF, Ando N. Domain Movements upon Activation of Phenylalanine Hydroxylase Characterized by Crystallography and Chromatography-Coupled Small-Angle X-ray Scattering. *J Am Chem Soc* **138**, 6506-6516 (2016).

18. Guinier A, Fournet G. Correction of measurements of low-angle X-ray scattering. *Nature* **160**, 501 (1947).

19. Xu K*, et al.* Neural migration. Structures of netrin-1 bound to two receptors provide insight into its axon guidance mechanism. *Science* **344**, 1275-1279 (2014).

20. Cowieson NP*, et al.* Beamline B21: high-throughput small-angle X-ray scattering at Diamond Light Source. *J Synchrotron Radiat* **27**, 1438-1446 (2020).
