# Supplementary material for: The dynamic nature of netrin-1 and the structural basis for glycosaminoglycan fragment-induced filament formation
Source: Nat Commun. 2023 Mar 3;14:1226. doi: 10.1038/s41467-023-36692-w (PMC9984387; doi:10.1038/s41467-023-36692-w)
Supplement: Supplementary file 2 — Description of Additional Supplementary Files [file 41467_2023_36692_MOESM2_ESM.pdf]

Description of Additional Supplementary Files

File name: Supplementary Data 1

Description: Gallery of SEC-SAXS 3D_electron_density_reconstructions.pdf: Gallery of DENSS 3D electron density reconstructions of monomeric NET1ΔC, dimeric NET1ΔC, NET1ΔC with HO-dp8 and NET1ΔC with HO-dp10. An averaged electron density model and a selection of refined models that fit the SEC-SAXS data are shown.

File name: Supplementary Data 2

Description: data_SEC-SAXS.zip: SEC-SAXS scattering data. Contains integrated frames, evolving factor analysis, scattering profiles, indirect Fourier transforms, Guinier analysis.

File name: Supplementary Data 3

Description: DNA_primers_list.xlsx: DNA primers used for cloning the protein constructs.

File name: Supplementary Movie 1

Description: MD simulation of netrin-1 with HO-dp10.mp4: A 100 ns molecular dynamics simulation of NET1ΔC with HO-dp10.

File name: Supplementary Movie 2

Description: MD simulation of netrin-1 with HO-dp24.mp4: A 100 ns molecular dynamics simulation of NET1ΔC with HO-dp24.

File name: Supplementary Movie 3

Description: SECSAXS 3D electron density reconstructions.webm: A video of DENSS 3D electron density reconstructions of monomeric NET1ΔC, dimeric NET1ΔC, NET1ΔC with HO-dp8 and NET1ΔC with HO-dp10.
