# Supplementary material for: The dynamic nature of netrin-1 and the structural basis for glycosaminoglycan fragment-induced filament formation
Source: Nat Commun. 2023 Mar 3;14:1226. doi: 10.1038/s41467-023-36692-w (PMC9984387; doi:10.1038/s41467-023-36692-w)

Gallery of 3D electron density reconstructions of Netrin-1 ΔC by SEC-SAXS

Authors

Markus Meier[1#], Monika Gupta[1#], Serife Akgül[2,3#], Matthew McDougall[1#], Thomas Imhof[2], Denise Nikodemus[4], Raphael Reuten[5,6], Aniel Moya-Torres[1], Vu To[1], Fraser Ferens[1], Fabian Heide[1], Gay Pauline Padilla-Meier[1], Philipp Kukura[7], Wenming Huang[3], Birgit Gerisch[3], Matthias Mörgelin[8], Kate Poole[9], Adam Antebi[3,10*], Manuel Koch[2,11,12*] & Jörg Stetefeld[1*]

[*]Corresponding authors: aantebi@age.mpg.de, manuel.koch@uni-koeln.de, jorg.stetefeld@umanitoba.ca
# These authors contributed equally

Affiliations

[1]Department of Chemistry, University of Manitoba, Canada
[2]Center for Biochemistry II, Faculty of Medicine and University Hospital Cologne, University of Cologne, 50931, Cologne, Germany.
[3]Max Planck Institute for Biology of Ageing, Cologne, Germany
[4]Faculty of Biology, Institute of Biology II, Albert Ludwigs University of Freiburg, Germany
[5]Institute of Experimental and Clinical Pharmacology and Toxicology, Medical Faculty, University of Freiburg, Freiburg, Germany.
[6]Department of Obsterics and Gynecology, Medical Center, University of Freiburg, Freiburg, Germany.
[7]Physical and Theoretical Chemistry Laboratory, Department of Chemistry, University of Oxford, South Parks Road, Oxford OX1 3QZ, UK.
[8]Colzyx AB, Lund, Sweden
[9]Max Delbrück Center for Molecular Medicine, Robert Roessle Str 10, Berlin-Buch, Germany
Current address: EMBL Australia Node in Single Molecule Science, School of Medical Sciences, Faculty of Medicine, University of New South Wales, Sydney, NSW, Australia
[10]Cologne Excellence Cluster on Cellular Stress Responses in Aging Associated Diseases, University of Cologne, Cologne 50931, Germany
[11]Institute for Dental Research and Oral Musculoskeletal Biology, Faculty of Medicine and University Hospital Cologne, University of Cologne, 50931, Cologne, Germany.
[12]Center for Molecular Medicine Cologne, Faculty of Medicine and University Hospital Cologne, University of Cologne, 50931, Cologne, Germany.

SEC-SAXS electron density reconstructions of deconvoluted NET1ΔC monomer

Experiment ID: sm22113-7/sample11

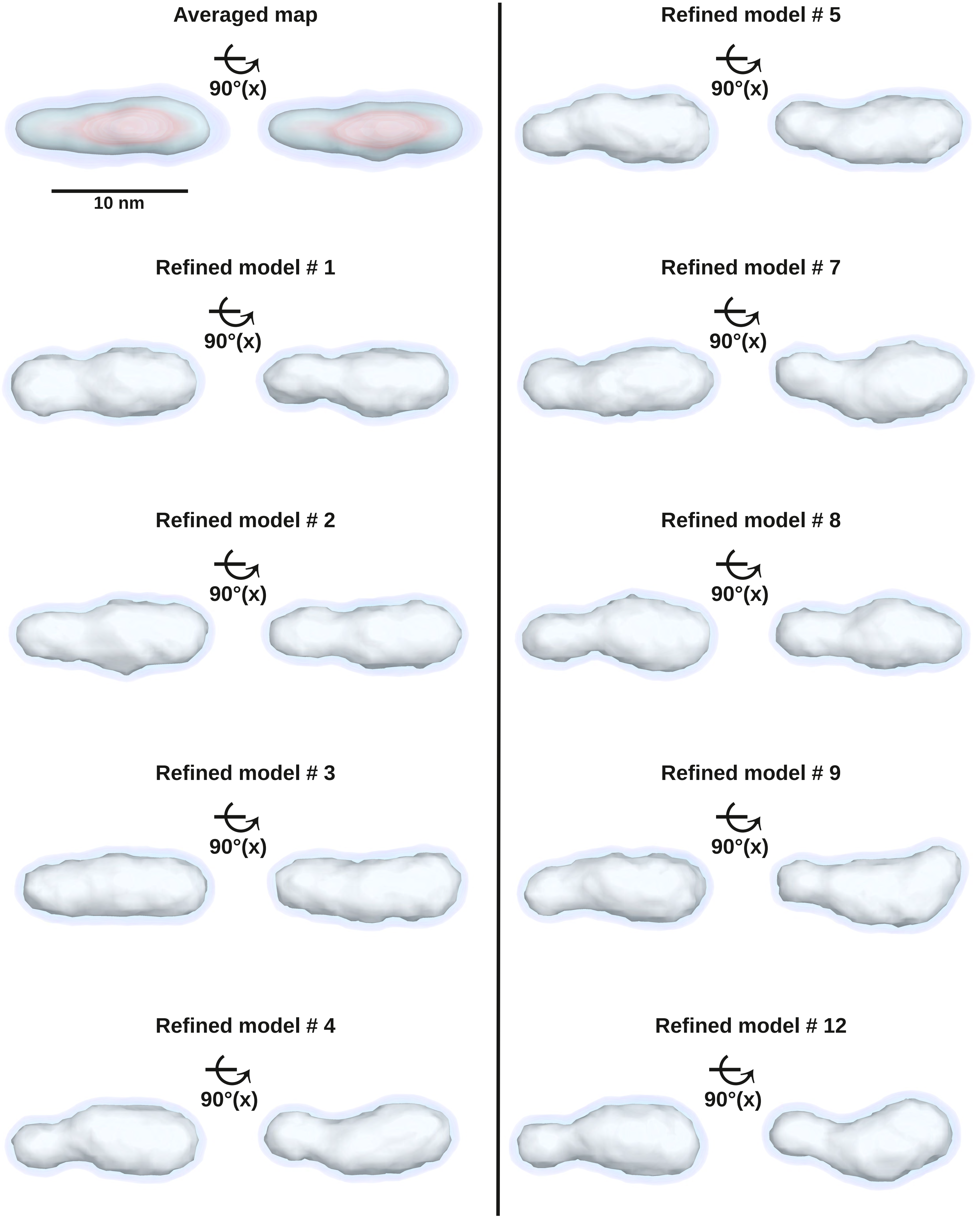

SEC-SAXS electron density reconstructions of deconvoluted NET1ΔC monomer

Experiment ID: sm22113-7/sample11

Refined model # 13

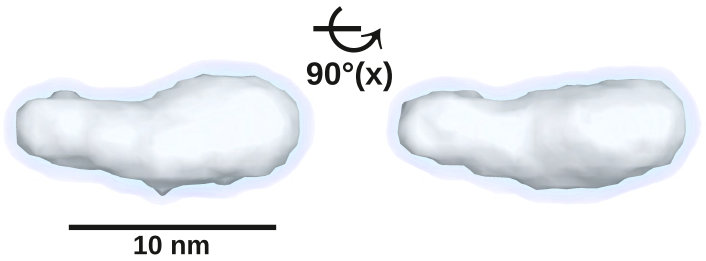

Refined model # 15

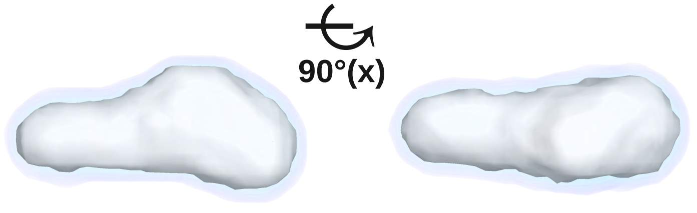

Refined model # 16

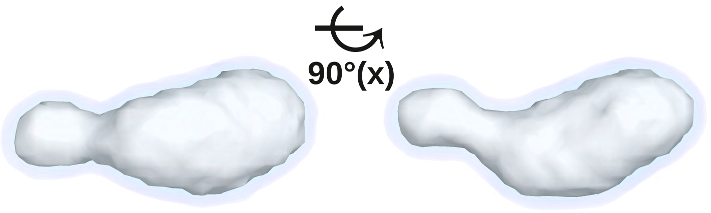

Refined model # 17

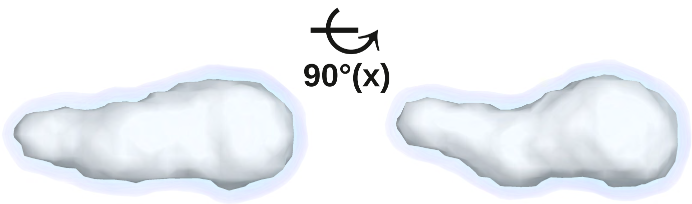

Refined model # 18

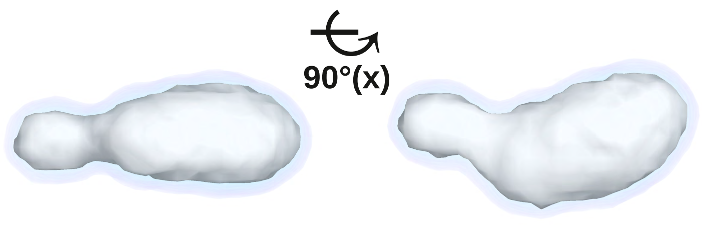

Refined model # 19

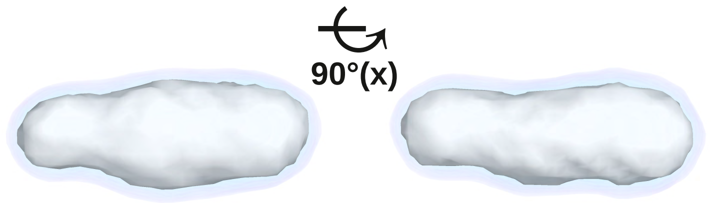

Refined model # 21

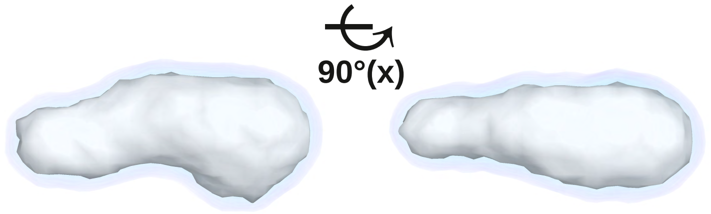

Refined model # 22

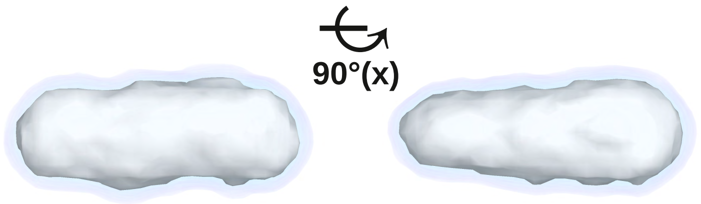

Refined model # 23

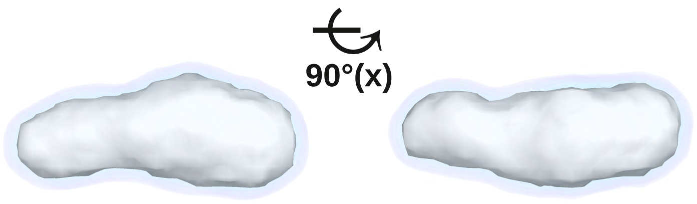

Refined model # 25

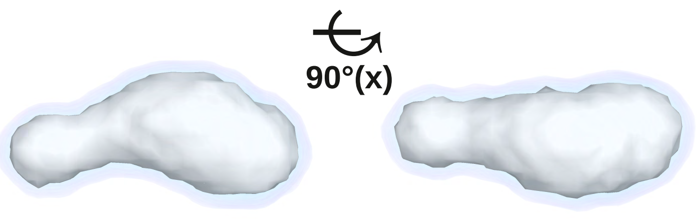

SEC-SAXS electron density reconstructions of deconvoluted NET1ΔC monomer

Experiment ID: sm22113-7/sample1

Averaged map

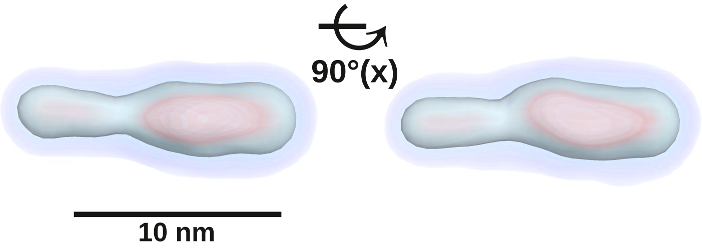

Refined model # 3

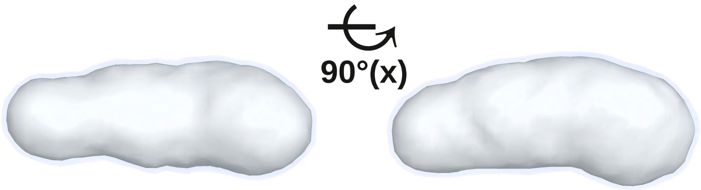

Refined model # 4

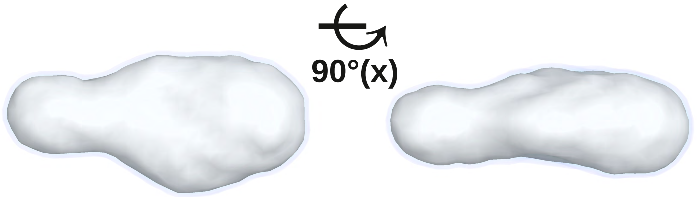

Refined model # 5

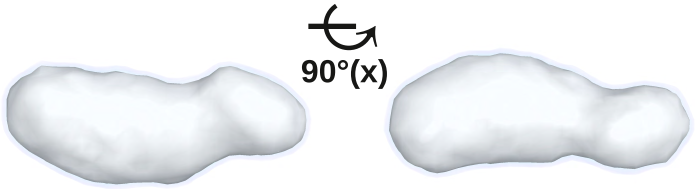

Refined model # 6

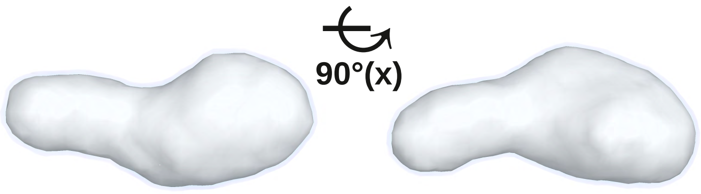

Refined model # 8

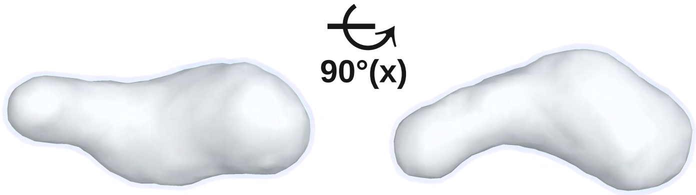

Refined model # 9

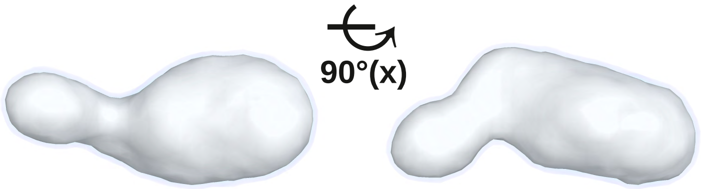

Refined model # 10

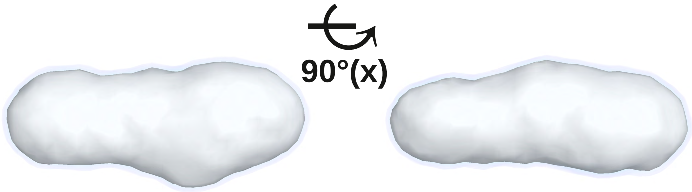

Refined model # 11

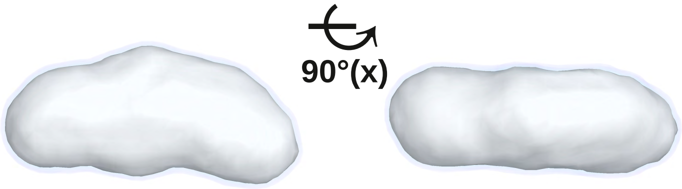

Refined model # 12

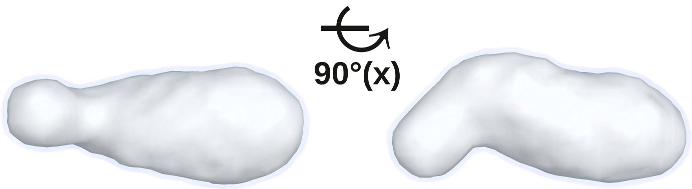

SEC-SAXS electron density reconstructions of deconvoluted NET1ΔC monomer

Experiment ID: sm22113-7/sample1

Refined model # 13

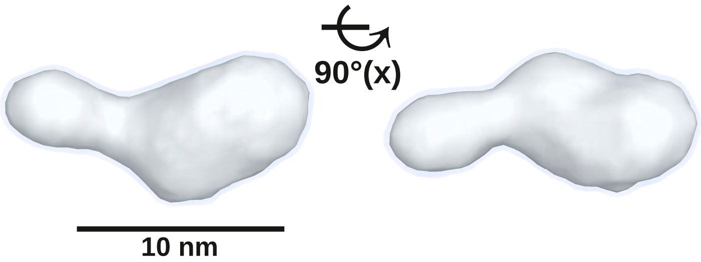

Refined model # 14

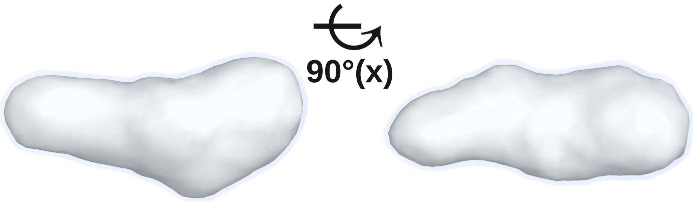

Refined model # 15

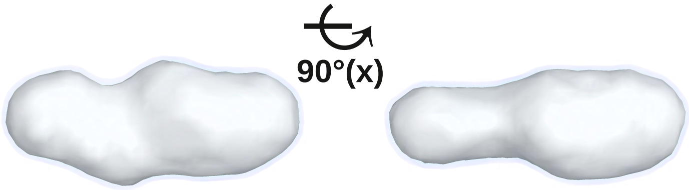

Refined model # 16

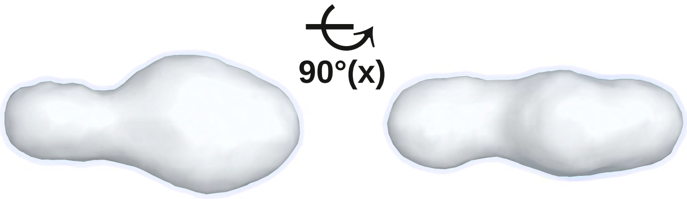

Refined model # 17

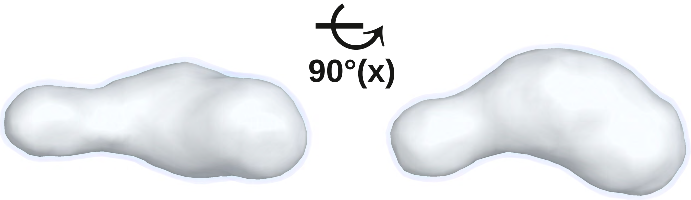

Refined model # 18

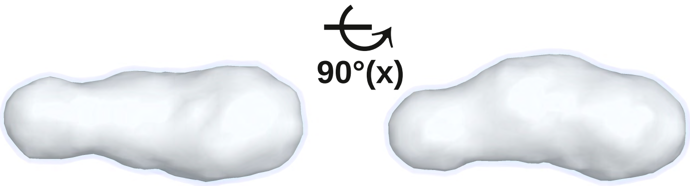

Refined model # 20

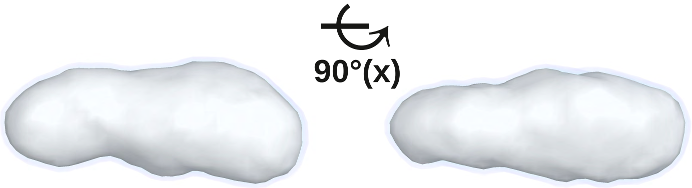

Refined model # 21

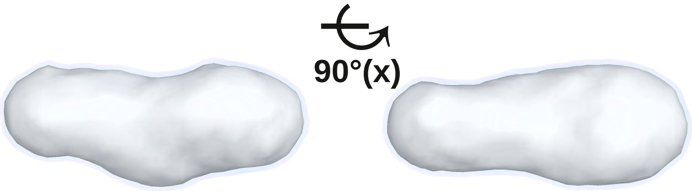

Refined model # 23

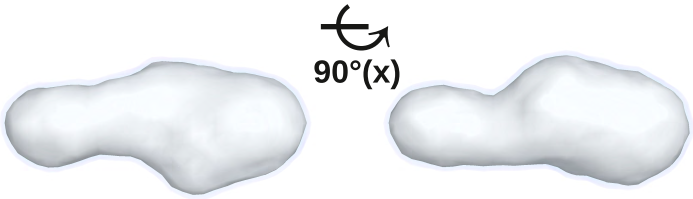

Refined model # 24

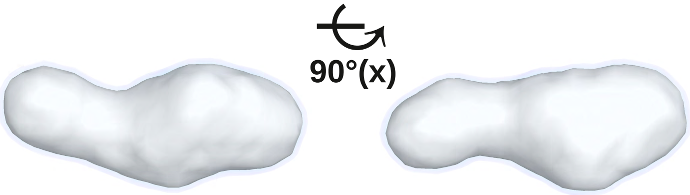

SEC-SAXS electron density reconstructions of deconvoluted NET1ΔC dimer

Experiment ID: sm22113-7/sample11

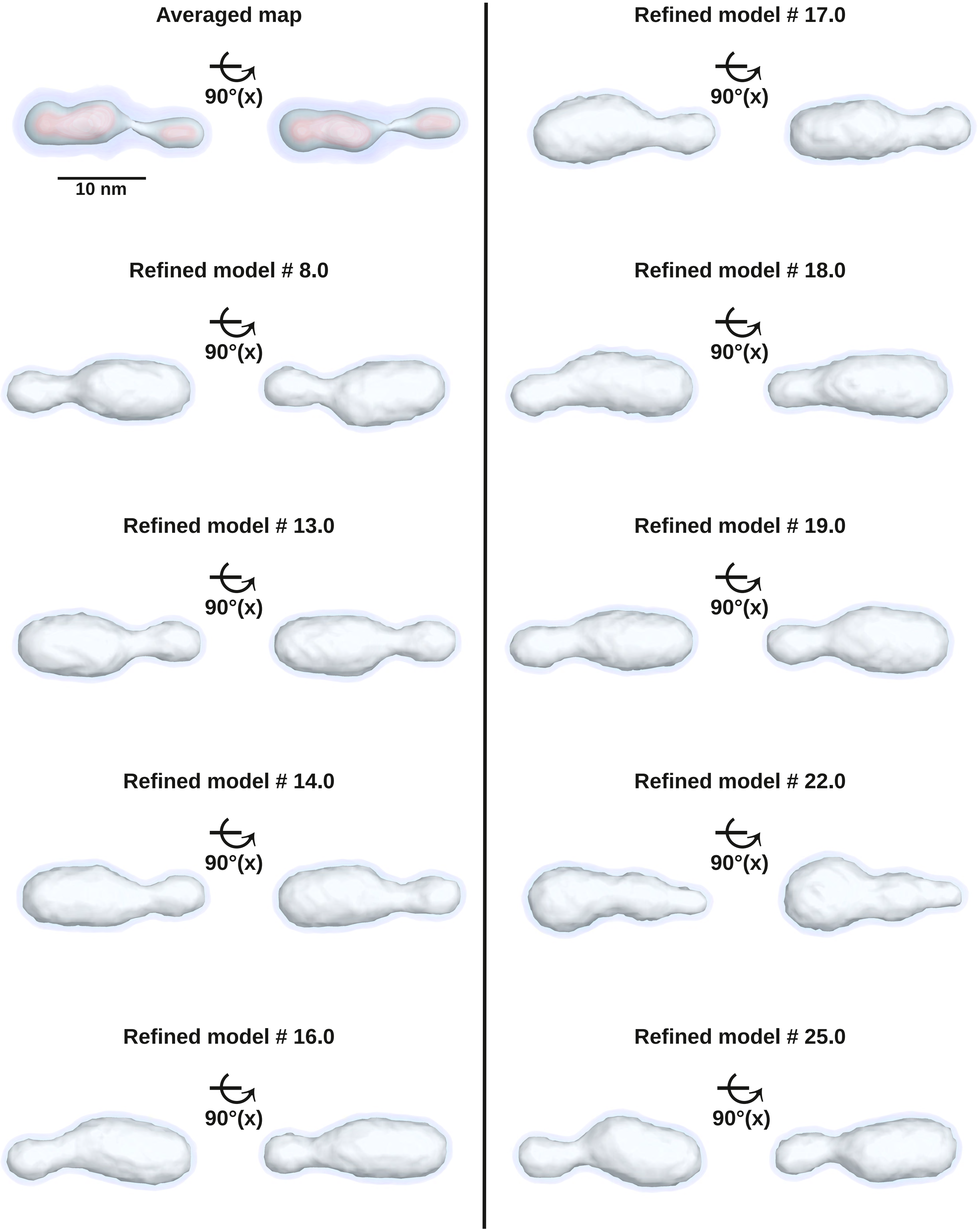

SEC-SAXS electron density reconstructions of deconvoluted NET1ΔC dimer

Experiment ID: sm22113-7/sample11

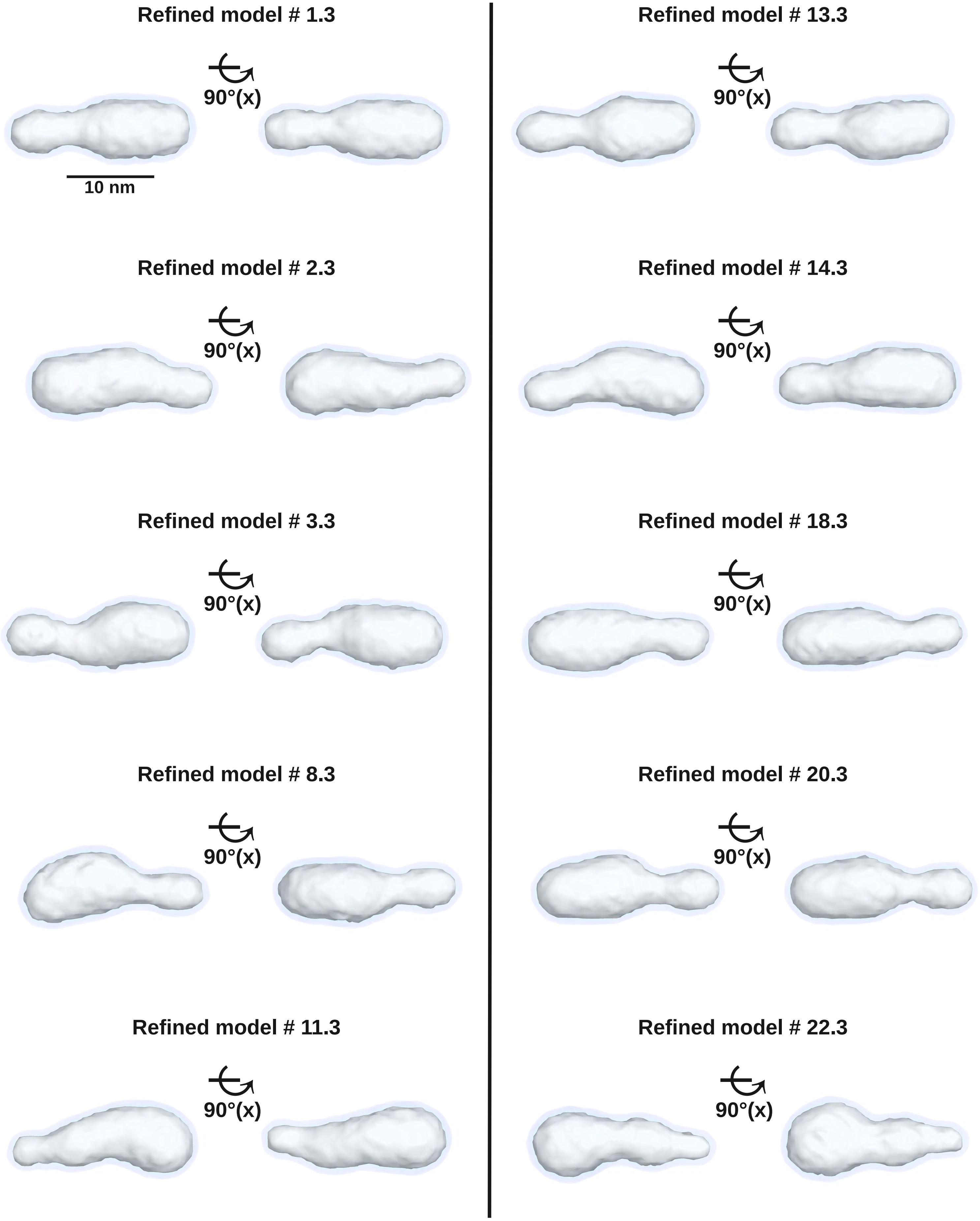

SEC-SAXS electron density reconstructions of deconvoluted NET1ΔC dimer

Experiment ID: sm22113-7/sample1

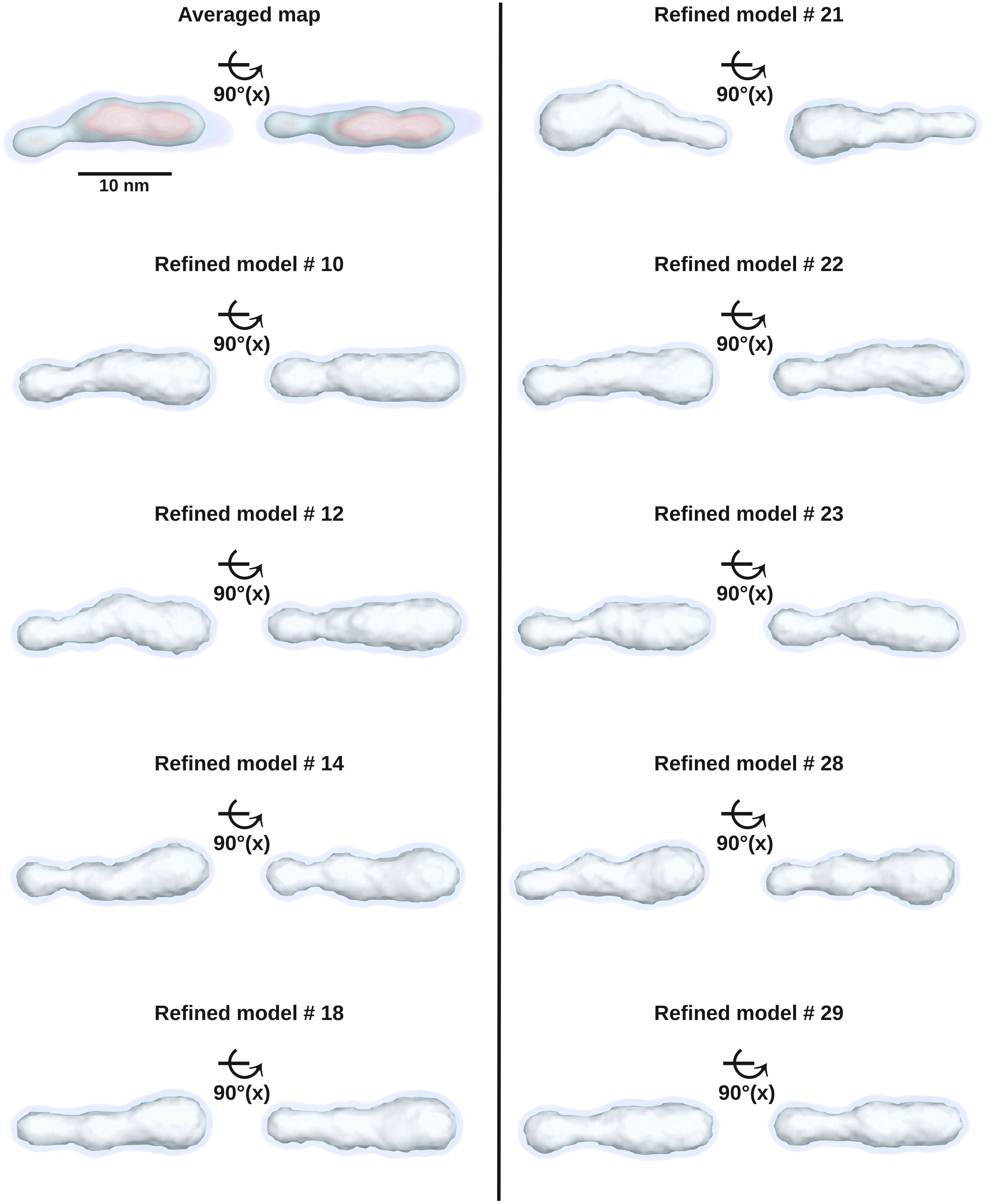

SEC-SAXS electron density reconstructions of deconvoluted NET1ΔC dimer

Experiment ID: sm22113-7/sample1

Refined model # 30

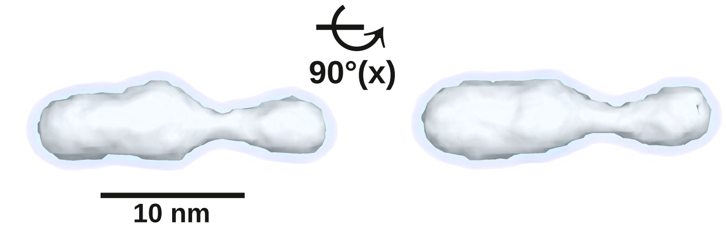

Refined model # 31

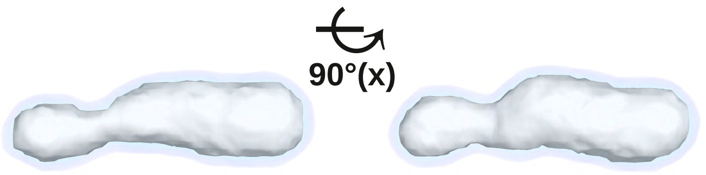

Refined model # 32

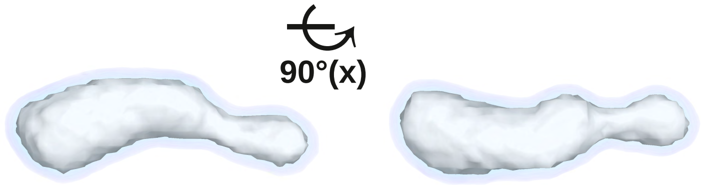

Refined model # 33

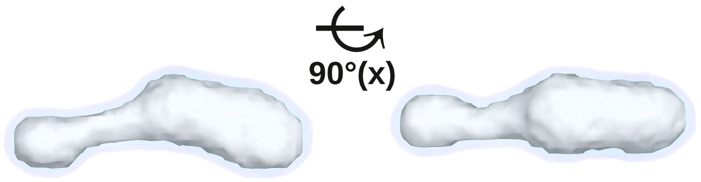

Refined model # 36

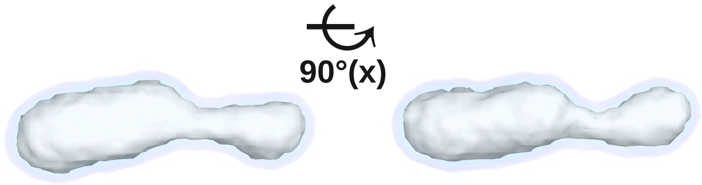

Refined model # 38

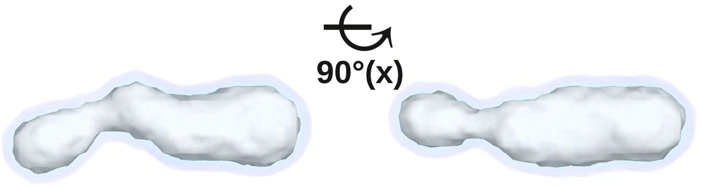

Refined model # 39

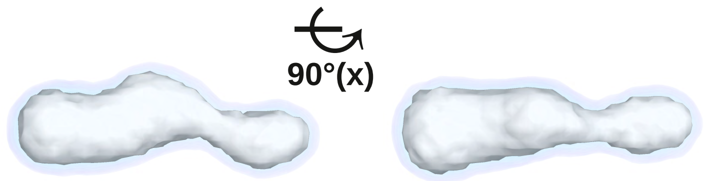

Refined model # 40

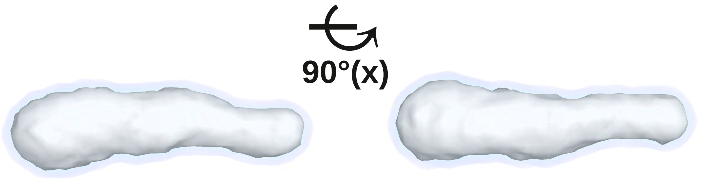

Refined model # 48

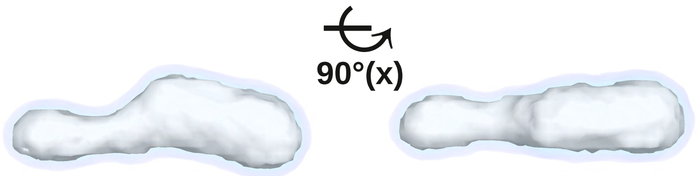

Refined model # 49

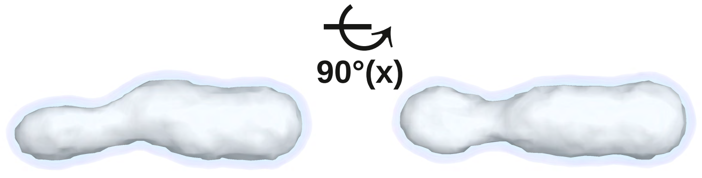

SEC-SAXS electron density reconstructions of NET1ΔC with heparin oligosaccharide dp8

Experiment ID: sm16028-7/379532

Averaged map

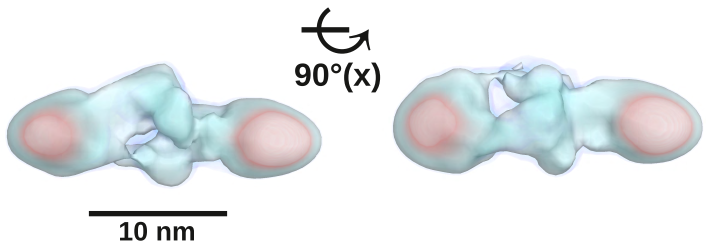

Refined model # 1

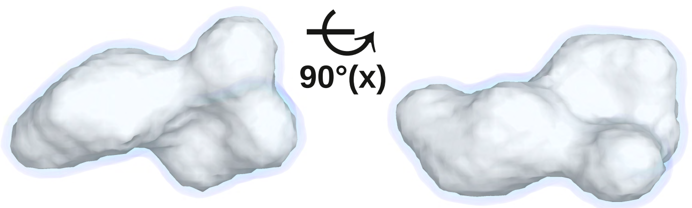

Refined model # 3

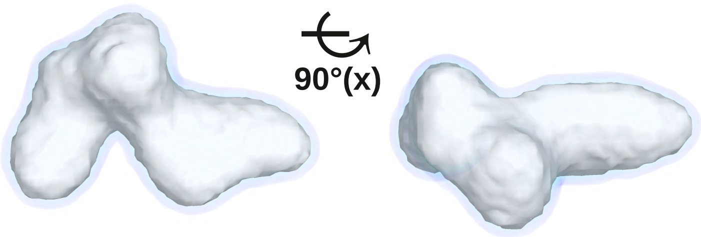

Refined model # 4

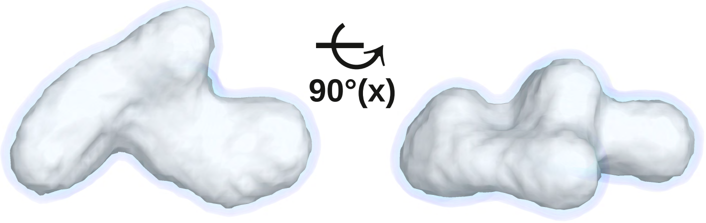

Refined model # 5

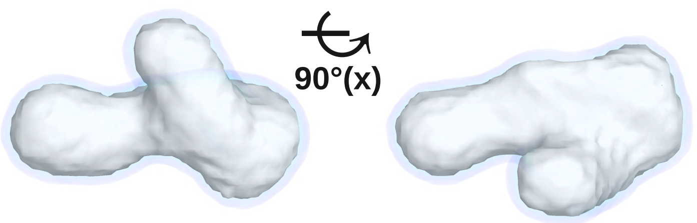

Refined model # 6

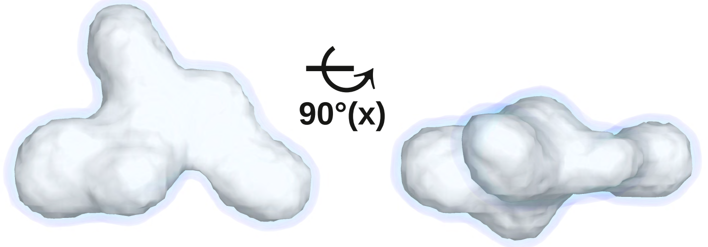

Refined model # 7

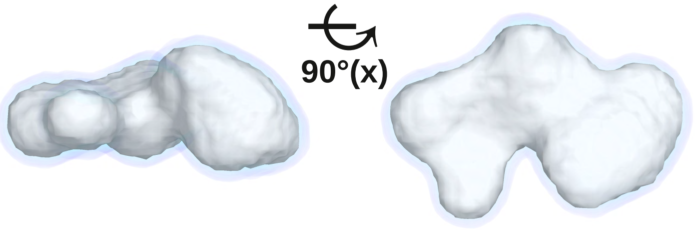

Refined model # 9

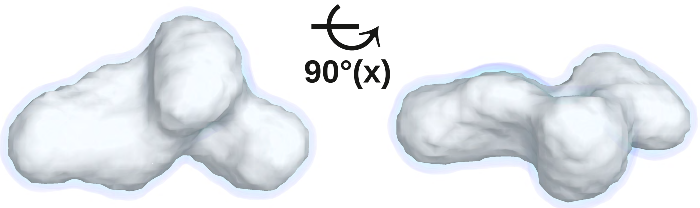

Refined model # 13

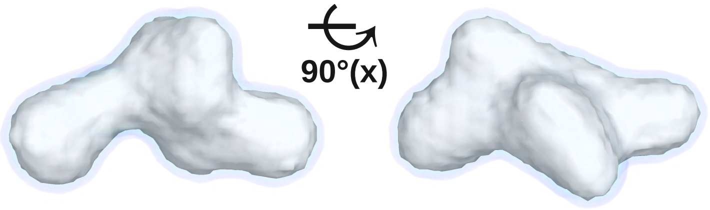

Refined model # 14

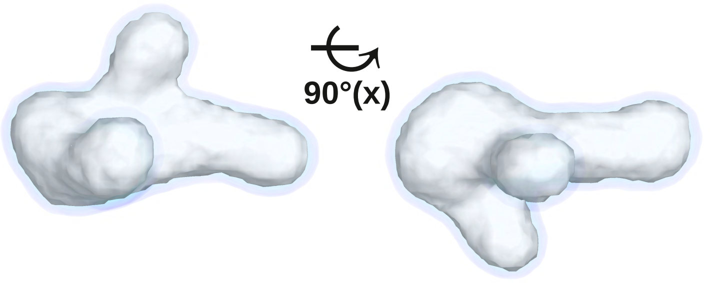

SEC-SAXS electron density reconstructions of NET1ΔC with heparin oligosaccharide dp8

Experiment ID: sm16028-7/379532

Refined model # 15

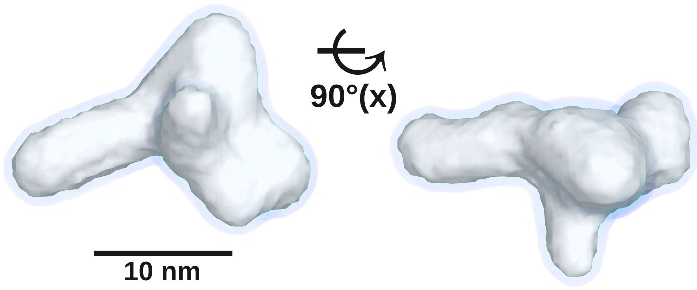

Refined model # 16

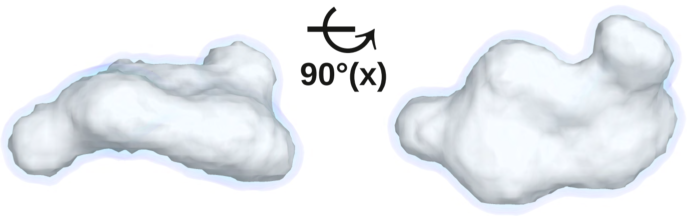

Refined model # 17

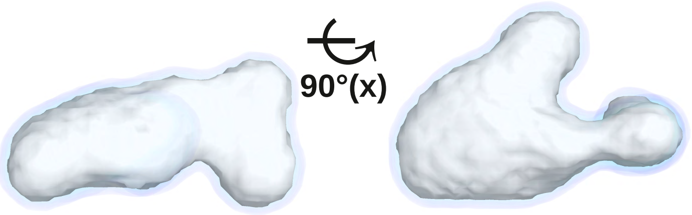

Refined model # 18

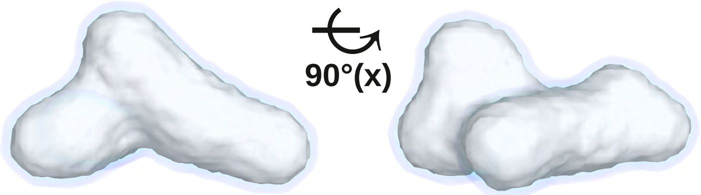

Refined model # 19

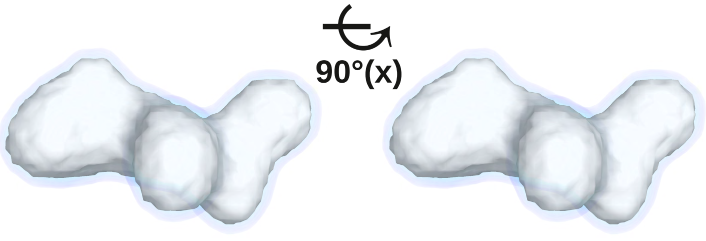

Refined model # 20

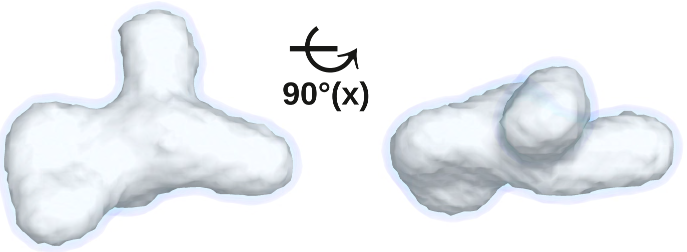

Refined model # 21

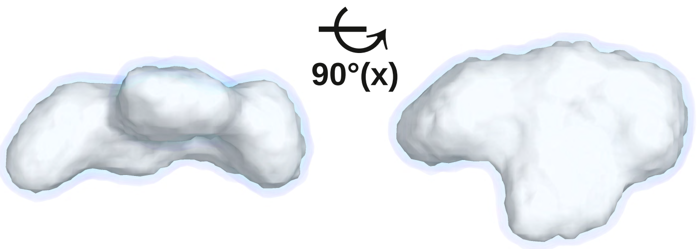

Refined model # 22

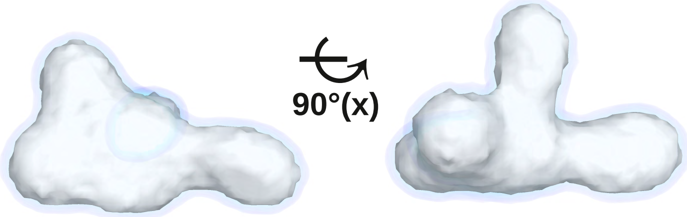

Refined model # 23

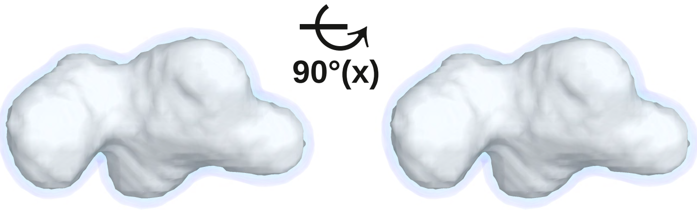

Refined model # 24

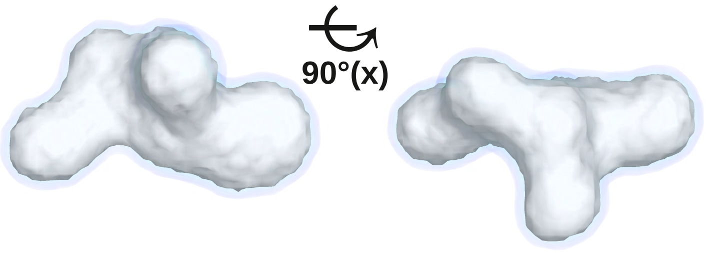

SEC-SAXS electron density reconstructions of NET1ΔC with heparin oligosaccharide dp8

Experiment ID: sm16028-7/379539

Averaged map

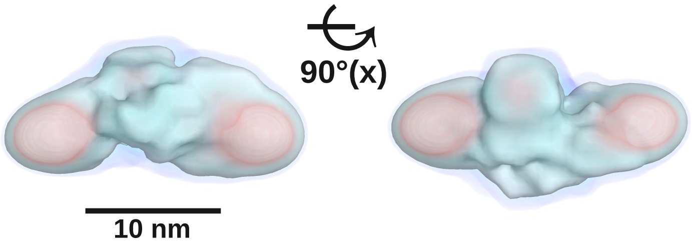

Refined model # 1

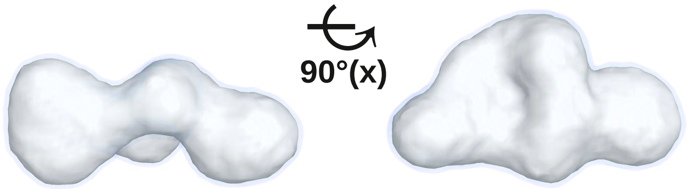

Refined model # 2

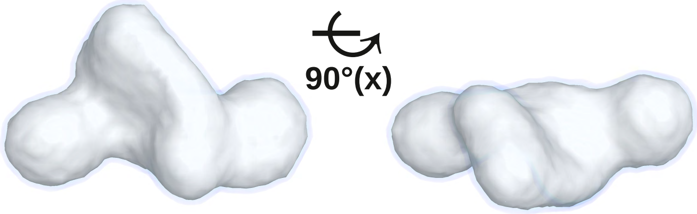

Refined model # 3

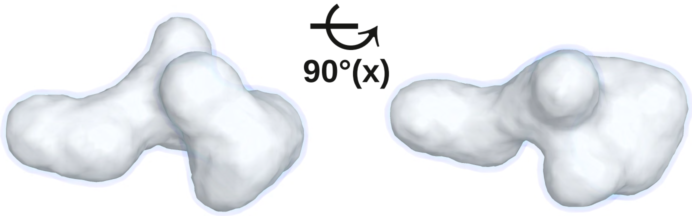

Refined model # 4

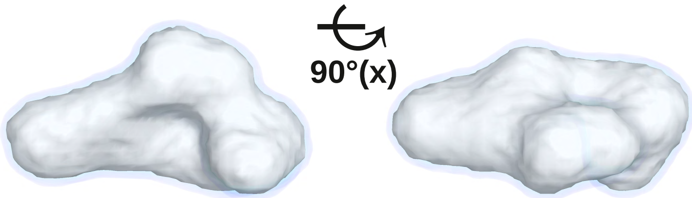

Refined model # 5

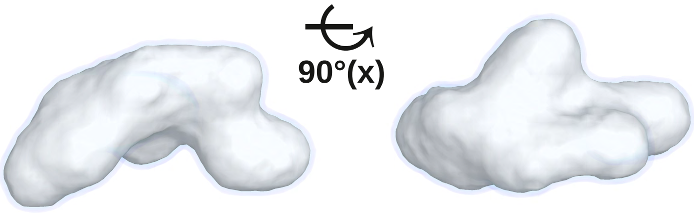

Refined model # 6

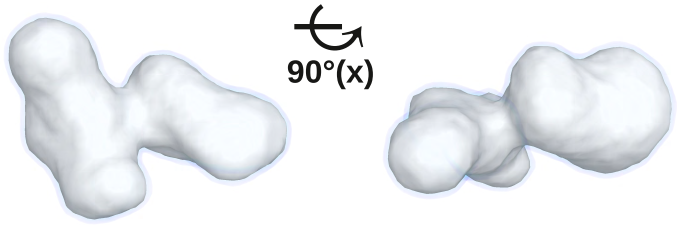

Refined model # 7

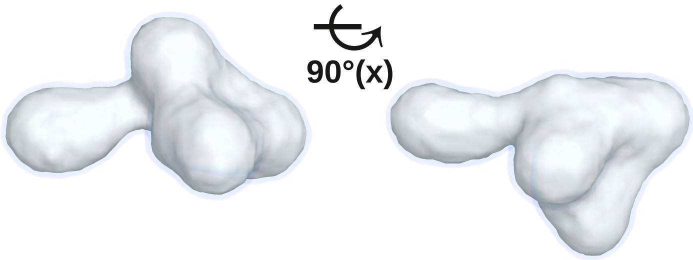

Refined model # 8

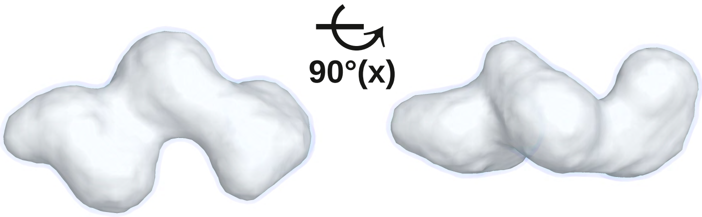

Refined model # 10

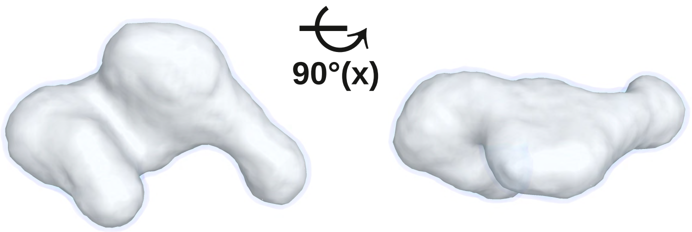

SEC-SAXS electron density reconstructions of NET1ΔC with heparin oligosaccharide dp8

Experiment ID: sm16028-7/379539

Refined model # 11

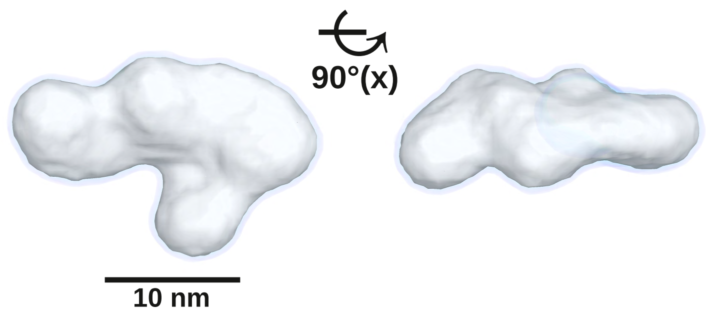

Refined model # 12

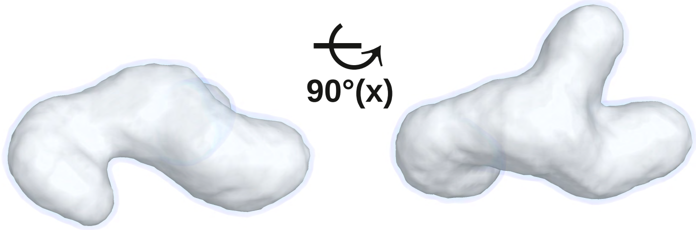

Refined model # 13

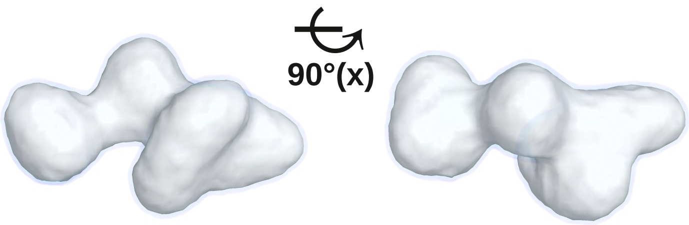

Refined model # 15

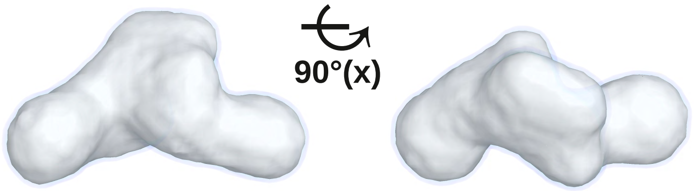

Refined model # 17

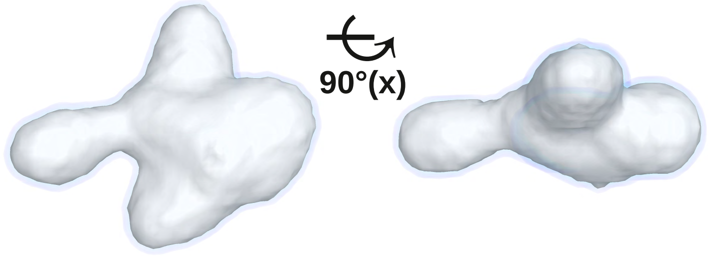

Refined model # 19

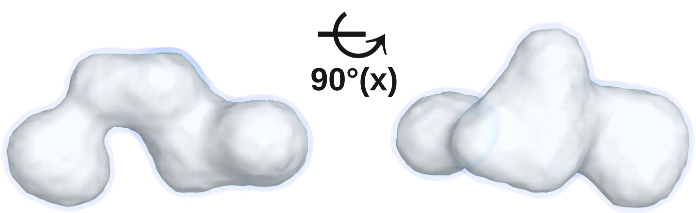

Refined model # 20

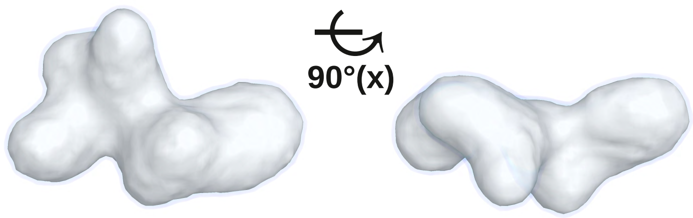

Refined model # 21

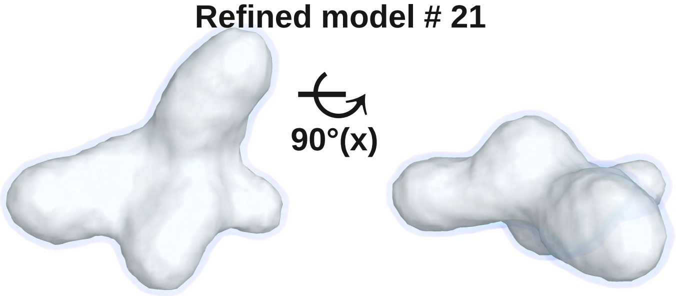

Refined model # 23

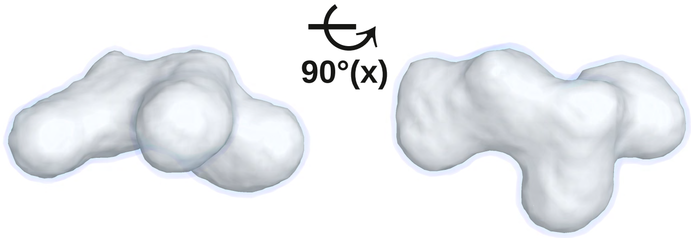

Refined model # 25

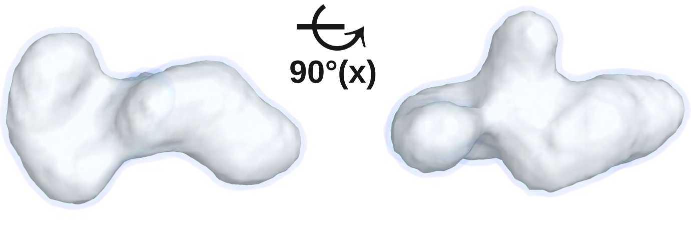

SEC-SAXS electron density reconstructions of NET1ΔC with heparin oligosaccharide dp10

Experiment ID: sm16028-7/379533

Averaged map

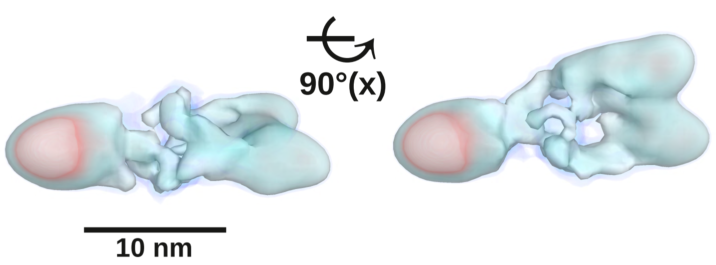

Refined model # 1

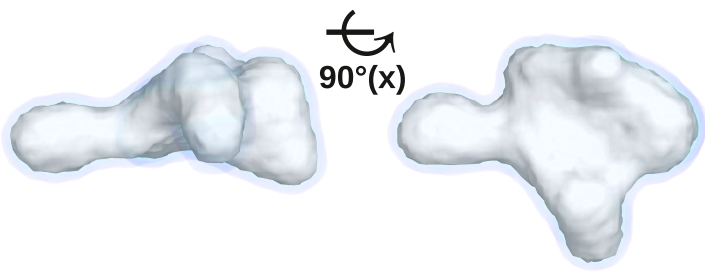

Refined model # 2

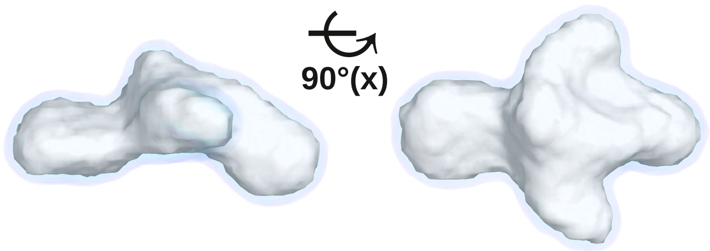

Refined model # 4

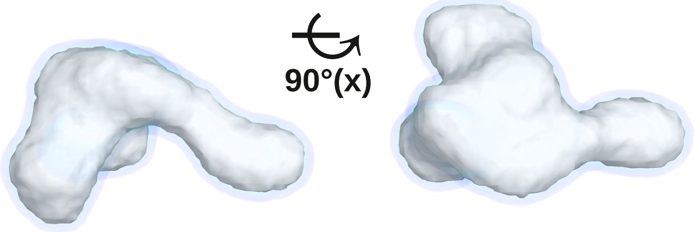

Refined model # 5

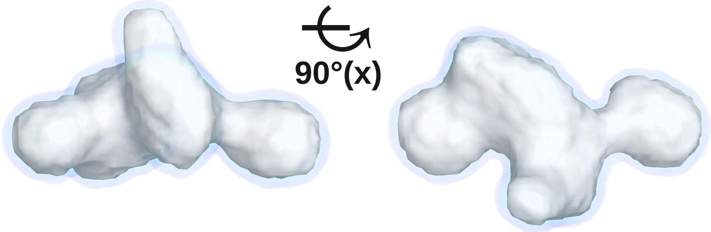

Refined model # 6

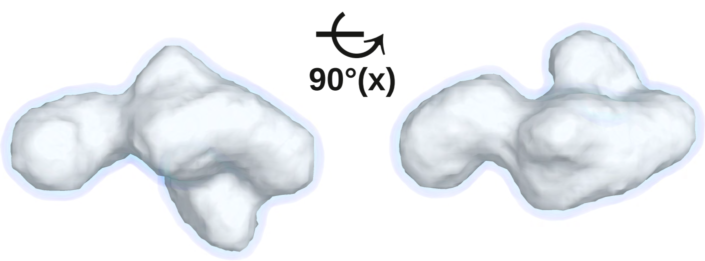

Refined model # 7

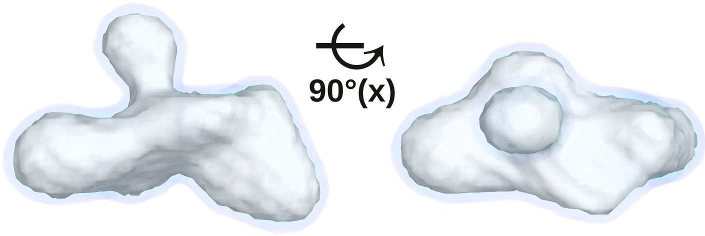

Refined model # 8

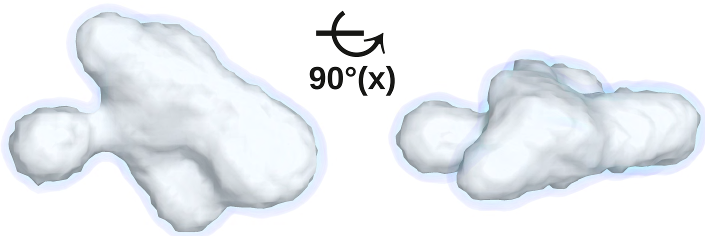

Refined model # 9

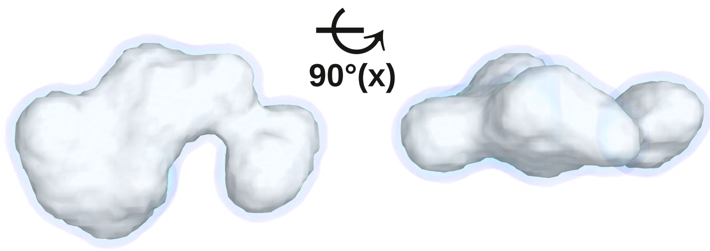

Refined model # 10

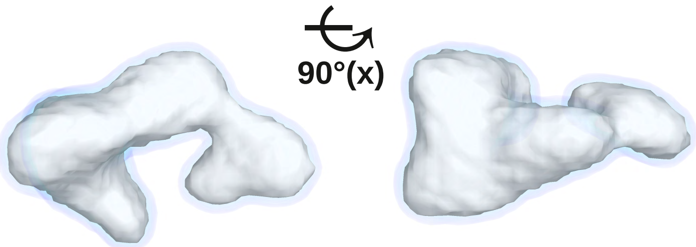

SEC-SAXS electron density reconstructions of NET1ΔC with heparin oligosaccharide dp10

Experiment ID: sm16028-7/379533

Refined model # 13

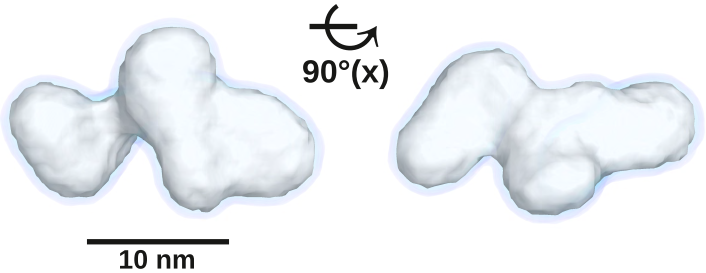

Refined model # 14

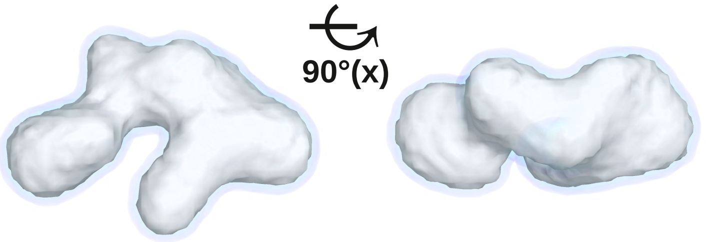

Refined model # 16

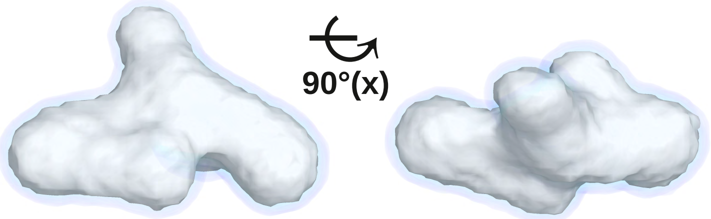

Refined model # 17

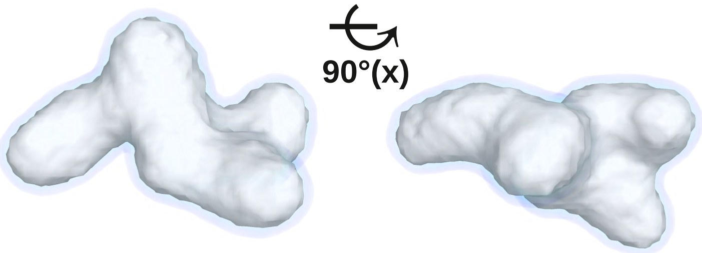

Refined model # 20

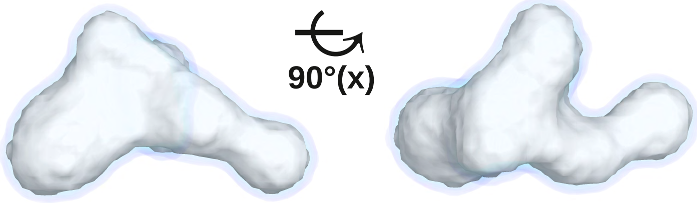

Refined model # 21

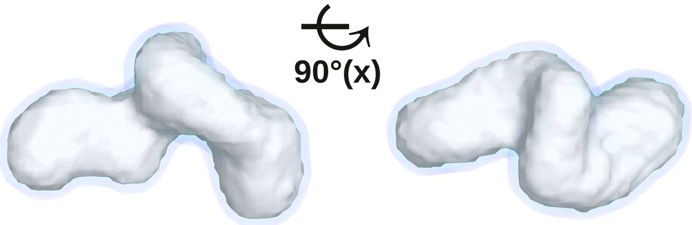

Refined model # 22

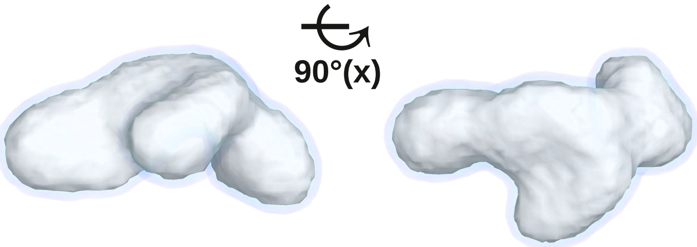

Refined model # 23

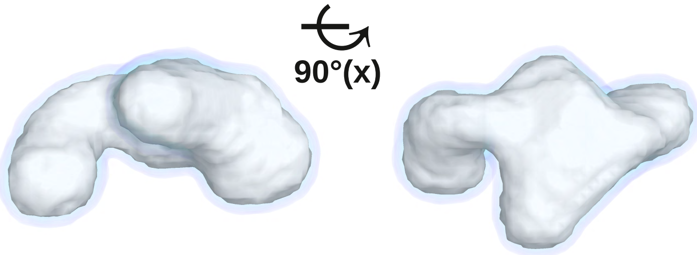

Refined model # 24

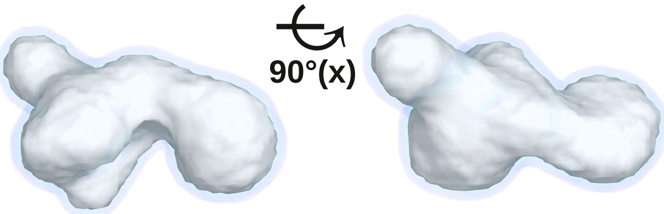

Refined model # 25

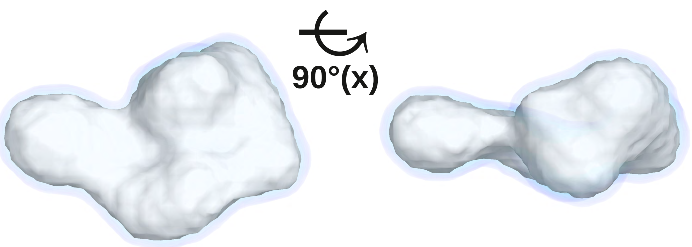

SEC-SAXS electron density reconstructions of NET1ΔC with heparin oligosaccharide dp10

Experiment ID: sm16028-7/379540

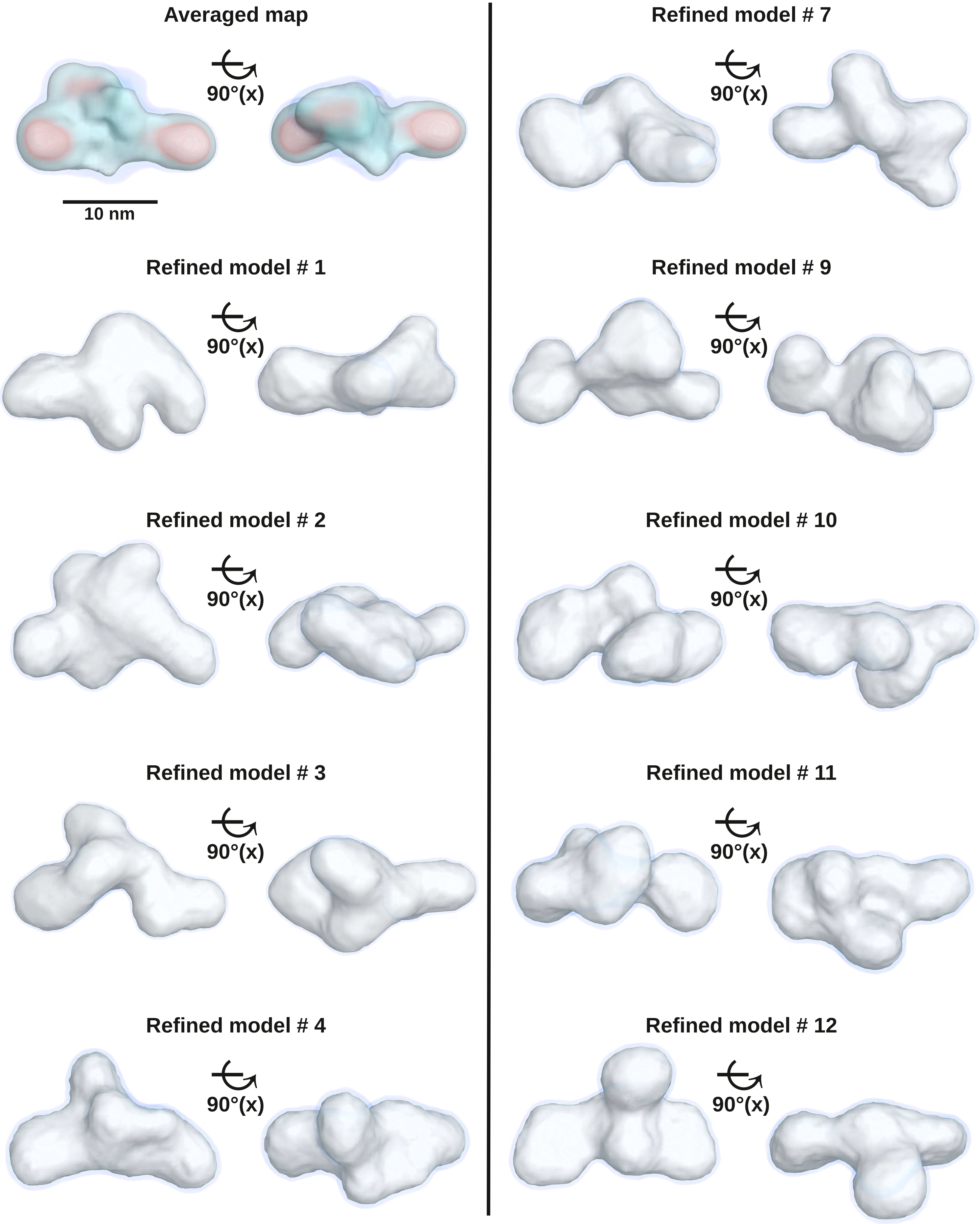

SEC-SAXS electron density reconstructions of NET1ΔC with heparin oligosaccharide dp10

Experiment ID: sm16028-7/379540

Refined model # 13

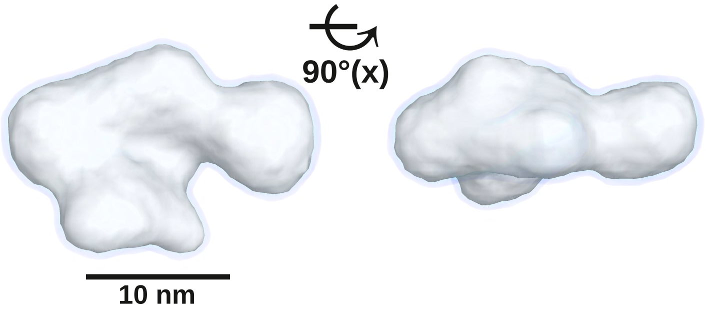

Refined model # 14

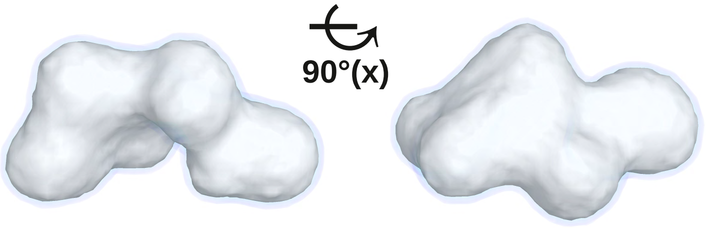

Refined model # 15

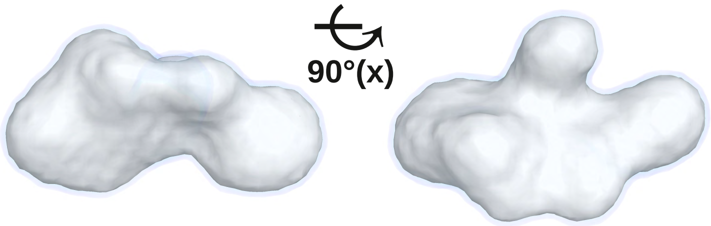

Refined model # 16

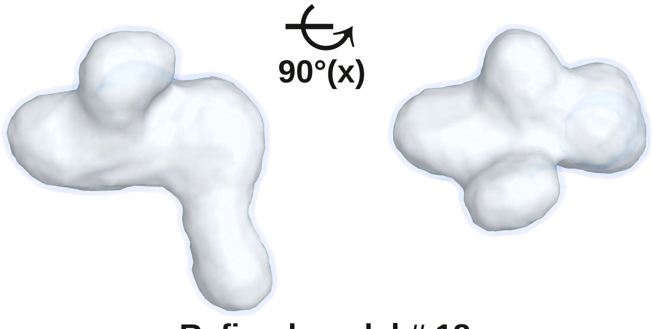

Refined model # 18

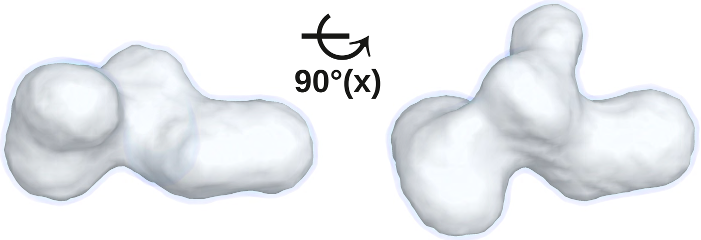

Refined model # 19

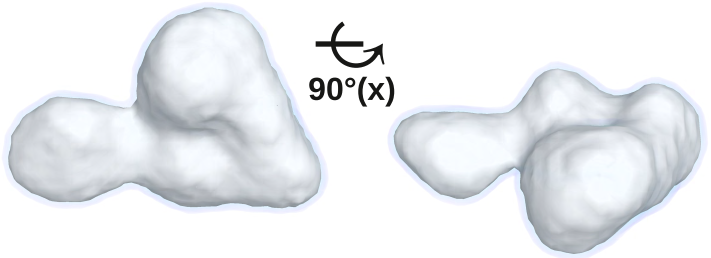

Refined model # 20

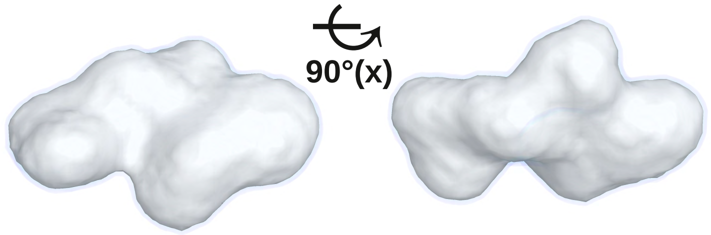

Refined model # 22

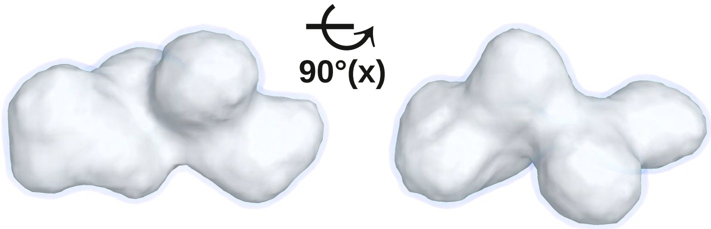

Refined model # 23

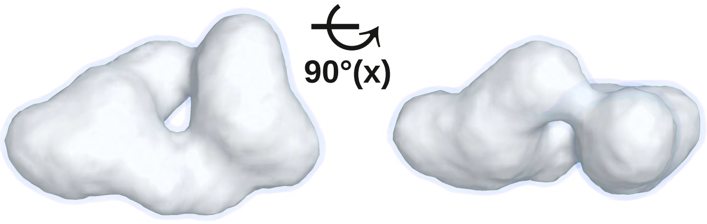

Refined model # 24

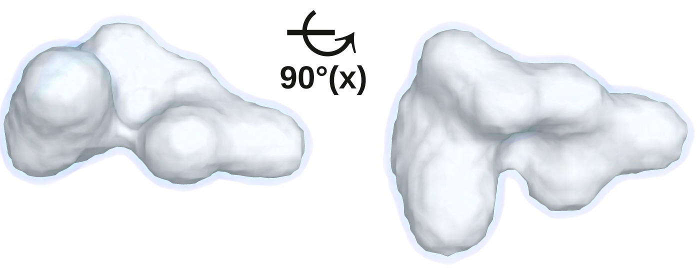

Supplement: Supplementary file 5 — Supplementary Data 3 [file 41467_2023_36692_MOESM5_ESM.pdf]
