# Supplementary material for: The dynamic nature of netrin-1 and the structural basis for glycosaminoglycan fragment-induced filament formation
Source: Nat Commun. 2023 Mar 3;14:1226. doi: 10.1038/s41467-023-36692-w (PMC9984387; doi:10.1038/s41467-023-36692-w)
Supplement: Supplementary file 9 — Reporting Summary [file 41467_2023_36692_MOESM9_ESM.pdf]

nature portfolio

Corresponding author(s): Jörg Stetefeld

Last updated by author(s): Feb 9, 2023

Reporting Summary

Nature Portfolio wishes to improve the reproducibility of the work that we publish. This form provides structure for consistency and transparency in reporting. For further information on Nature Portfolio policies, see our Editorial Policies and the Editorial Policy Checklist.

Statistics

For all statistical analyses, confirm that the following items are present in the figure legend, table legend, main text, or Methods section.

n/a	Confirmed	
☐	☒	The exact sample size (*n*) for each experimental group/condition, given as a discrete number and unit of measurement
☐	☒	A statement on whether measurements were taken from distinct samples or whether the same sample was measured repeatedly
☐	☒	The statistical test(s) used AND whether they are one- or two-sided<br>*Only common tests should be described solely by name; describe more complex techniques in the Methods section.*
☐	☒	A description of all covariates tested
☐	☒	A description of any assumptions or corrections, such as tests of normality and adjustment for multiple comparisons
☐	☒	A full description of the statistical parameters including central tendency (e.g. means) or other basic estimates (e.g. regression coefficient) AND variation (e.g. standard deviation) or associated estimates of uncertainty (e.g. confidence intervals)
☐	☒	For null hypothesis testing, the test statistic (e.g. *F*, *t*, *r*) with confidence intervals, effect sizes, degrees of freedom and *P* value noted<br>*Give P values as exact values whenever suitable.*
☒	☐	For Bayesian analysis, information on the choice of priors and Markov chain Monte Carlo settings
☒	☐	For hierarchical and complex designs, identification of the appropriate level for tests and full reporting of outcomes
☒	☐	Estimates of effect sizes (e.g. Cohen's *d*, Pearson's *r*), indicating how they were calculated

Our web collection on statistics for biologists contains articles on many of the points above.

Software and code

Policy information about availability of computer code

Data collection	X-ray crystallography: HKL3000 721.3 (HKL Research Inc.), MxDC (08B1-1 @ Canadian Light Source)<br>SEC-SAXS: bioSAXS instrument software (B21 @ Diamond Light Source Ltd.), ChemStation 2.169.0.0 (Agilent)<br>SEC-MALS: ASTRA 7.3.2.21 (Wyatt Technology), UNICORN 7.3 (Cytiva)<br>Sedimentation velocity: ProteomeLab 6.2 (Beckman-Coulter)<br>Mass photometry: AcquireMP 2.4.2 (Refeyn)<br>Molecular Dynamics Simulation: GROMACS 2021.3 (Free Software, LGPL v. 2.1)<br>Negative stain: iTEM™ (Olympus)<br><br>Commercial software packages from other developers used for the C.elegans experiments in this study are:<br>Confocal microscopy: Leica Application Suite X 3.5.7.23225 (LASER-SCANNING CONFOCAL SP8-DLS);<br>Zeiss microscopy: Axio Vision SE64Rel.4.9.1
Data analysis	Sequence conservation analysis: MultAlin 5.4.2 (Florence Corpet, National Research Institute for Agriculture, Food and Environment), ESPript 3.0 (Patrice Gouet & Xavier Robert, French National Centre for Scientific Research)<br>X-ray crystallography: PHENIX 1.19.2-4158 (Phenix development team), CCP4 7.1 (Collaborative Computational Project No. 4), Coot 0.9.6 (Paul Emsley, MRC Laboratory of Molecular Biology), PyMOL 2.4.1 (Schrödinger LLC), ChimeraX 1.2.5 (UCSF Resource for Biocomputing, Visualization, and Informatics, University of California San Francisco), PDBePISA 1.5.2 (Protein Data Bank in Europe), APBS GIT commit 86adcf1 (Pacific Northwest National Laboratory), Phaser 2.8.3 (Cambridge Institute for Medical Research), SWISS-MODEL (Swiss Institute of Bioinformatics) , Procheck (CCP4), XDS 202231 (Wolfgang Kabsch,

Max Planck Institute for Medical Research), Aimless 0.7.7 (CCP4), FastContact 2.0 (C. J. Camacho, C. Zhang & P. C. Champ, University of Pittsburgh)
SEC-SAXS: ATSAS 3.0.4 (EMBL), SCÅTTER 3.2d (Robert Rambo, Diamond Light Source Ltd.), DENSS GIT commit c03ef8b (Thomas Grant, SUNY University at Buffalo), QtiPlot 1.0.0-rc15 (IONDEV SRL), Hydro Suite (Jose García de la Torre, Universidad de Murcia), GMFIT 20190921 (Takeshi Kawabata, Osaka University), Chimera 1.14.0 (UCSF Resource for Biocomputing, Visualization, and Informatics, University of California San Francisco)
SEC-MALS: ASTRA 7.3.2.21 (Wyatt Technology), UNICORN 7.3 (Cytiva), R 4.0.5 (The R Foundation of Statistical Computing), Matplotlib 3.4.2 (Matplotlib development team, open source license)
Sedimentation velocity: Sedfit 16.1c, Sedphat 15.2b (Peter Schuck, National Institutes of Health), GUSSI 1.4.2 (Chad A. Brautigam, University of Texas Southwestern)
Mass photometry: Discover MP 2.4.2 (Refeyn)
Molecular Dynamics Simulation: GROMACS 2021.3 (Free Software, LGPL v. 2.1), Modeller 10.1 (University of California San Francisco), GLYCAM (Robert J. Woods, University of Georgia), VMD 1.9.4a51 (NIH Center for Macromolecular Modeling & Bioinformatics), ACPYPE (ACPYPE development team, Bio2Byte Group)
Negative stain 2D-aligment and averaging: EMAN 2.3.1 (Baylor College of Medicine), Fiji ImageJ 1.53f51 (Wayne Rasband and contributors, public domain)

Commercial software packages from other developers used for the C.elegans experimentes in this study are:
Statistical analysis and graph preparation: GraphPad Prism 9.0.0 (86); https://www.graphpad.com/
Quantification of puncta size and number: IMARIS (Imaris v.8.2.1)

For manuscripts utilizing custom algorithms or software that are central to the research but not yet described in published literature, software must be made available to editors and reviewers. We strongly encourage code deposition in a community repository (e.g. GitHub). See the Nature Portfolio guidelines for submitting code & software for further information.

Data

Policy information about availability of data

All manuscripts must include a data availability statement. This statement should provide the following information, where applicable:
- Accession codes, unique identifiers, or web links for publicly available datasets
- A description of any restrictions on data availability
- For clinical datasets or third party data, please ensure that the statement adheres to our policy

The X-ray crystallography datasets and structural models from this study have been deposited in the Protein Data Bank [https://www.rcsb.org] under accession codes 7LRF [https://dx.doi.org/10.2210/pdb7LRF/pdb] and 7LER [https://dx.doi.org/10.2210/pdb7LER/pdb]. Other coordinates and structure factors used as a reference in this study are available in the Protein Data Bank [https://www.rcsb.org] under accession codes 4OVE [https://dx.doi.org/10.2210/pdb4OVE/pdb] and 3IRJ [https://dx.doi.org/10.2210/pdb3IRJ/pdb]. The DENSS 3D electron density reconstructions from SEC-SAXS were deposited to the Small Angle Scattering Biological Data Bank (SASBDB) under accession code SASDRJ2 [https://www.sasbdb.org/data/SASDRJ2], SASDRK2 [https://www.sasbdb.org/data/SASDRK2], SASDRL2 [https://www.sasbdb.org/data/SASDRL2], SASDRM2 [https://www.sasbdb.org/data/SASDRM2], SASDRN2 [https://www.sasbdb.org/data/SASDRN2], SASDRP2 [https://www.sasbdb.org/data/SASDRP2], SASDRQ2 [https://www.sasbdb.org/data/SASDRQ2], SASDRR2 [https://www.sasbdb.org/data/SASDRR2] and additional scattering data are included with the supplement. Protein-ligand model and simulation trajectory files of the molecular dynamics simulation are available on the Center for Open Science (OSF) database [https://dx.doi.org/10.17605/osf.io/kn4q2]. SEC-MALS and sedimentation velocity raw data sets are available from the authors on request. The python scripts we wrote for electron density volume calculations are available from the authors on request. All other data generated in this study are included in the published article and supplement. Source data are provided with this paper.

Human research participants

Policy information about studies involving human research participants and Sex and Gender in Research.

Reporting on sex and gender	Not applicable to this study
Population characteristics	Not applicable to this study
Recruitment	Not applicable to this study
Ethics oversight	Not applicable to this study

Note that full information on the approval of the study protocol must also be provided in the manuscript.

Field-specific reporting

Please select the one below that is the best fit for your research. If you are not sure, read the appropriate sections before making your selection.

☒ Life sciences ☐ Behavioural & social sciences ☐ Ecological, evolutionary & environmental sciences

For a reference copy of the document with all sections, see nature.com/documents/nr-reporting-summary-flat.pdf

Life sciences study design

All studies must disclose on these points even when the disclosure is negative.

Sample size	No statistical methods were used for sample size determination. Exact sample sizes are indicated in the corresponding figure legends and supplementary tables.<br>Sample size for experiments performed on C. elegans DTC, Vulva and AVM/PVM neurons were chosen based on literature in which similar experiments on same genes were performed. If smaller samples sized were choosen, these were justified by obtaining same/similar results compared to the used literature (Blanchette et al.,PLoS Biol. 2015; MacNeil et al.,Nature Neuroscience 2009; Suzuki et al., Dev. Biol. 2006; Estes et al., Dev. Biol. 2009).<br>Sample sizes for quantification of fluorescences puncta/spots in C. elegans were determined according to our laboratory experience and other studies using these assays (Goya et al.,Cell Reports 2020; Schiffer et al., elife 2021).
Data exclusions	Independent scorings performed on C elegans with less than 10 animals were disregarded. This happened for the Vulva scoring of two genotypes: lon-2(e678) & lon-2(e678);unc-6(syb2327). The criteria were established before conducting the experiments, 10 worms per biological repeat is the minimum ''technical'' sample size necessary for reliable statistics.<br>For the neurite outgrowth assay, an exclusion criteria was established that any preparations in which no neurite outgrowth was observed would be removed from analysis (however, this situation did not arise in the course of the study).
Replication	For C. elegans studies of DTC, Vulva and PVM/AVM neurons a minimum of three biological replicates analyzing 20 or 30 worms were performed to verify the reproducibility of the findings. Note: For the wildtype genotype N2, more than 10 biological replicates were performed for all experiments.<br>For the heparin binding deficient mutants unc-6(C,D) the DTC experiment was independently performed with six biological replicates. To emphasize the variety of observed phenotypes, DTC defect classes were introduced. Since this classification was not considered for the initial aforementioned experiments the data were not included to the statistical analysis of the most recent data used in this manuscript. However, both experiments verified the observed phenotype for the heparin binding deficient mutants unc-6(C,D).<br>For the quantification of fluorescence puncta/spots in worms a minimum of three biological replicates analyzing 11 worms performed to verify the reproducibility of the findings.<br>For the neurite outgrowth assay, measurements were obtained from cultures derived from at least 4 mice and at least 5 printed coverslips.
Randomization	For all C. elegans experiments (DTC, Vulva, Neuron, puncta quantification) young adult stage hermaphrodites were randomly picked from our maintenance plates and used for eggs lays.<br>When the progeny reach the desired developmental stage for the performed experiment, they were randomly picked and analyzed. Different genotypes and conditions were assessed in random order.<br>Regarding the neurite outgrowth assay, the dorsal root ganglia were separated into parallel samples applied across all test substrates, thus no randomisation into separate sample groups was required.
Blinding	The DTC, Vulva and Neuron experiments in C. elegans, for all the genotypes were analyzed blinded. Genotypes with obvious phenotypes were group in a way that ensured blinding. Therefore, it was possible to perform all experiments blinded.<br>The fluorescence puncta/spot experiment in C . elegans were not blinded. However, worms were randomly picked and assigned to the different conditions and the different conditions were assessed in random order. Further, the experiment was designed to use computational analysis to ensure an non-biased outcome. Moreover, all the critical experiments were repeated independently by at least three times.

Reporting for specific materials, systems and methods

We require information from authors about some types of materials, experimental systems and methods used in many studies. Here, indicate whether each material, system or method listed is relevant to your study. If you are not sure if a list item applies to your research, read the appropriate section before selecting a response.

Materials & experimental systems

n/a	Involved in the study	
☐	☒	Antibodies
☐	☒	Eukaryotic cell lines
☒	☐	Palaeontology and archaeology
☐	☒	Animals and other organisms
☒	☐	Clinical data
☒	☐	Dual use research of concern

Methods

n/a	Involved in the study	
☒	☐	ChIP-seq
☒	☐	Flow cytometry
☒	☐	MRI-based neuroimaging

Antibodies

Antibodies used	NET1 polyclonal rabbit antibody, lab-made, affinity purified (1:500)(ELISA)

	DCC polyclonal rabbit antibody, lab-made, affinity purified (1:2000)(ELISA)<br>Neogenin polyclonal rabbit antibody, lab-made, affinity purified (1:500)(ELISA)<br>Unc5b polyclonal rabbit antiobady, lab-made, affinity purified (1:3000)(ELISA)<br>Biotin-HRP conjugated antibody, catalogue # OAIA00064 (Aviva Systems Biology) (1:4000)(ELISA)<br>Swine Anti-Rabbit Immunoglobulins/HRP, catalogue # P039901-2 (Agilent Dako) (1:3000)(ELISA)<br>Strep-Tactin® HRP conjugate, catalogue # 2-1502-001 (IBA Lifesciences) (1:4000)(ELISA)
Validation	Lab-made antibodies: Validation is demonstrated in this manuscript<br>Validation of antibodies was done via western blot studies comparing supernatants of transfected HEK293 cells with an expression constructs for the targeted protein and the empty vector. Cross reactivity of lab-made antibodies to relevant other protein targets used in this study were tested via western blot comparing supernatants of transfected HEK293 cells with expression constructs for the targeted proteins.<br><br>Aviva catalogue # OAIA00064 product data sheet: https://www.avivasysbio.com/sd/tds/html_datasheet.php?sku=OAIA00064<br>Agilent Dako # P039901-2 product data sheet: https://www.agilent.com/cs/library/msds/SDS445_EUGerman.pdf<br>IBA Lifesciences catalogue # 2-1502-001 product data sheet:https://www.iba-lifesciences.com/media/17/bf/a6/1657539201/DS_2-1502_ST-HRP-conj.pdf

Eukaryotic cell lines

Policy information about cell lines and Sex and Gender in Research

Cell line source(s)	Human embryonic kidney (HEK) 293 EBNA cells, Invitrogen Catalog# R620907 (for protein expression)<br>Dorsal root ganglia from Mus musculus, dissected from mice, aged 4-6 weeks
Authentication	Cell lines were not authenticated.
Mycoplasma contamination	Cell lines were regularly tested for Mycoplasma contamination using a PCR-based approach and were confirmed to be Mycoplasma-free.
Commonly misidentified lines<br>(See ICLAC register)	No misidentified cell lines were used.

Animals and other research organisms

Policy information about studies involving animals; ARRIVE guidelines recommended for reporting animal research, and Sex and Gender in Research

Laboratory animals	Caenorhabditis elegans wild-type (N2 Bristol strain).<br>Two independent Crispr/Cas Caenorhabditis elegans strains were generated for the unc-6 heparin binding deficient mutants unc-6(27) & unc-6(28) (B,C: NP_509165: B: R378, H379A and C: H403A, R404A, K405A; obtained by Suny biotech).<br>Listed Caenorhabditis elegans mutants, obtained from Caenorhabditis elegans Genetics Center and outcrossed to wild type (N2 Bristol strain).<br>The late L4/young adult stage hermaphrodites of following strains were used in DTC, Vulva and Neuron experiments.<br>AA5202 unc-6(syb2327)<br>AA5203 unc-6(syb2328)<br>NW434 unc-6(ev400) X<br>AA5349 N2;unc-52(e669)<br>AA1495 N2;lon-2(e678)<br>AH205 N2;sdn-1(zh20) X.<br>AA5350 N2; unc-5(e152)<br>AA5351 N2; unc-40(n342)<br>AA5427 unc-6(syb2327) X; unc-40(n342)<br>AA5426 unc-6(syb2328) X; unc-40(n342)<br>AA5424 unc-6(syb2328) X; unc-5(e152)<br>AA5425 unc-6(syb2327) X; unc-5(e152)<br>AA5420 unc-6(syb2328) X; lon-2(e678)<br>AA5421 unc-6(syb2327) X; lon-2(e678)<br>AA5422 unc-6(syb2328) X; sdn-1(zh20)<br>AA5423 unc-6(syb2327) X; sdn-1(zh20)<br>AA5418 unc-6(syb2328) X; unc-52(e669)<br>AA5419 unc-6(syb2327) X; unc-52(e669)<br>AA5495 lon-2(e678);unc-5(e152) IV<br>AA5496 lon-2(e678);unc-40(n324) I.<br>AA5362 N2; zdIs5 [mec-4p::GFP + lin-15(+)]<br>AA5440 sdn-1; zdIs5 [mec-4p::GFP + lin-15(+)]<br>AA5441 unc-52;zdIs5 [mec-4p::GFP + lin-15(+)]<br>AA5361 lon-2(e678); zdIs5 [mec-4p::GFP + lin-15(+)]<br>AA5438 unc-40; zdIs5 [mec-4p::GFP + lin-15(+)]

AA5439 unc-5; zdIs5 [mec-4p::GFP + lin-15(+)]
AA5366 unc-6(syb2327); zdIs5 [mec-4p::GFP + lin-15(+)]
AA5365 unc-6(syb2328); zdIs5 [mec-4p::GFP + lin-15(+)]
AA5364 unc-6(ev400); zdIs5 [mec-4p::GFP + lin-15(+)]
AA5437 unc-6(27);unc-40(n342); zdIs5 [mec-4p::GFP + lin-15(+)]
AA5436 unc-6(28);unc-40(n342); zdIs5 [mec-4p::GFP + lin-15(+)]
AA5434 unc-6(28);unc-5(e152); zdIs5 [mec-4p::GFP + lin-15(+)]
AA5435 unc-6(27);unc-5(e152) ; zdIs5 [mec-4p::GFP + lin-15(+)]
AA5431 unc-6(27);lon-2(e678); zdIs5 [mec-4p::GFP + lin-15(+)]
AA5430 unc-6(28);lon-2(e678);zdIs5 [mec-4p::GFP + lin-15(+)]
AA5432 unc-6(28);sdn-1(zh20); zdIs5 [mec-4p::GFP + lin-15(+)]
AA5433 unc-6(27);sdn-1(zh20); zdIs5 [mec-4p::GFP + lin-15(+)]
AA5428 unc-6(28);unc-52(e669); zdIs5 [mec-4p::GFP + lin-15(+)]
AA5429 unc-6(27);unc-52(e669);zdIs5 [mec-4p::GFP + lin-15(+)]
Day 1 and Day 9 adult stage hermaphrodites of following strains were used for puncta/spot counting experiments.
YC42 ghIs9(unc-6p::venus::unc-6) IV.
AA5468 lon-2(e678);ghIs9(unc-6p::venus::unc-6) IV.
Wild type C57Bl/6 mice

Wild animals	No wild animals were used in this study.
Reporting on sex	Sex was not considered in the study design. All the C. elegans strains used were hermaphrodite. Male mice were used to prepare the dorsal root ganglia primary cell lines.
Field-collected samples	No samples were collected in the field.
Ethics oversight	No ethical approval was required for the C. elegans study. No special license was required to work with mice as no experiments were performed on living mice. All mice were housed in a licensed animal housing facility at the Max Delbrück Center, Germany and were euthanized before isolating the primary cells in strict compliance with protocols approved by the German authorities (specifically: Landesamt für Gesundheit und Soziales, State of Berlin animal experimentation committee).

Note that full information on the approval of the study protocol must also be provided in the manuscript.
